# MicroRNA miR-29 controls a compensatory response to limit neuronal iron accumulation during adult life and aging

**DOI:** 10.1186/s12915-017-0354-x

**Published:** 2017-02-13

**Authors:** Roberto Ripa, Luca Dolfi, Marco Terrigno, Luca Pandolfini, Aurora Savino, Valeria Arcucci, Marco Groth, Eva Terzibasi Tozzini, Mario Baumgart, Alessandro Cellerino

**Affiliations:** 1 Scuola Normale Superiore, Laboratory of Biology (Bio@SNS), c/o Istituto di Biofisica del CNR, via 17 Moruzzi 1, 56124 Pisa, Italy; 2 Leibniz Institute on Aging – Fritz Lipmann Institute (FLI), Beutenbergstr. 11, 07745 Jena, Germany; 3 Wellcome Trust/Cancer Research UK Gurdon Institute, Tennis Court Road, Cambridge, CB2 1QN UK; 4 Babraham Research Campus, CB22 3AT Cambridge, UK

## Abstract

**Background:**

A widespread modulation of gene expression occurs in the aging brain, but little is known as to the upstream drivers of these changes. MicroRNAs emerged as fine regulators of gene expression in many biological contexts and they are modulated by age. MicroRNAs may therefore be part of the upstream drivers of the global gene expression modulation correlated with aging and aging-related phenotypes.

**Results:**

Here, we show that microRNA-29 (miR-29) is induced during aging in short-lived turquoise killifish brain and genetic antagonism of its function induces a gene-expression signature typical of aging. Mechanicistically, we identified *Ireb2* (a master gene for intracellular iron delivery that encodes for IRP2 protein), as a novel miR-29 target. MiR-29 is induced by iron loading and, in turn, it﻿﻿ reduces IRP2 expression in vivo, therefore limiting intracellular iron delivery in neurons. Genetically modified fish with neuro-specific miR-29 deficiency exhibit increased levels of IRP2 and transferrin receptor, increased iron content, and oxidative stress.

**Conclusions:**

Our results demonstrate that age-dependent miR-29 upregulation is an adaptive mechanism that counteracts the expression of some aging-related phenotypes and its anti-aging activity is primarily exerted by regulating intracellular iron homeostasis limiting excessive iron-exposure in neurons.

**Electronic supplementary material:**

The online version of this article (doi:10.1186/s12915-017-0354-x) contains supplementary material, which is available to authorized users.

## Background

MicroRNAs (miRNAs) are a class of small non-coding RNAs frequently present as small clusters spanning some kilobases in the genome, that negatively regulate gene expression by targeting mRNAs due to sequence complementarity, thereby inducing transcript degradation and/or translational inhibition [1]. A single miRNA shows complementarity to hundreds or thousands of different transcripts, an ability which gives miRNAs the potential to act as central regulatory nodes in gene co-expression networks [2], thereby modulating and providing stability to complex biological processes involving several interconnected signaling pathways such as aging. In recent years, specific miRNAs that regulate different aspects of the aging process were identified [3]. Several miRNAs were described to be significantly up- or down-regulated with age, both in invertebrate and vertebrate species [4, 5], some microRNAs influence lifespan and/or predict longevity in invertebrate models [6–9] and one of these (miR-34) regulates functional heart aging in mammals [10]. MicroRNA-29 family members (miR-29a, miR-29b and miR-29c) were shown to be upregulated during normal aging in the central nervous system (CNS) of different vertebrate species such as fish [11], mice [12–14], monkeys [5] and humans [5, 15]. Moreover, miR-29 appears upregulated in klotho-deficient mice [12], a model of premature aging, and progeroid mouse model *Zmpste24* ^–/–^ [14].

In mammals, the miR-29 family is present as two distinct genomic clusters that are both ubiquitously expressed and are particularly enriched in the CNS [13], specifically in mature neurons [16]. Mutant mice lacking both clusters do not present evident abnormalities at birth, but they rapidly accumulate defects during post-natal development and die within 6 weeks [17]. Mice lacking only one cluster (the miR-29b/a-1 locus), thus retaining residual miR-29 activity, reach adulthood, display an ataxic phenotype, a mild loss of Purkinje cells in the cerebellum, and die around 9 months of age [18]. A similar cerebellar phenotype is induced by knock-down of miR-29 in adult rodents [19]. On the other hand, up-regulation of miR-29 protects neurons against apoptosis during neuronal maturation, forebrain cerebral ischemia and stroke by targeting pro-apoptotic members of the BCL-2 family [20–22]. Interestingly, the miR-29 family was found to target Beta-Site APP-Cleaving Enzyme (BACE1) mRNA and to be downregulated in sporadic Alzheimer’s disease [23]. All these data indicate that miR-29 plays a protective role in neurons and reduced miR-29 activity in adult life has detrimental effects.

Iron is an element required for multiple fundamental biological processes such as mitochondrial ATP generation, DNA replication and synthesis of heme, myelin and dopamine [24]. While iron is an essential micronutrient, it also represents one of the most powerful sources of oxidative damage. Indeed, intermediates of oxygen reduction, including H_2_O_2_ and superoxide, are potent oxidants in the presence of Fe^2+^ and their reactions with Fe^2+^ form hydroxyl radicals, which cause cytotoxicity primarily due to lipid peroxidation [25]. This, along with the evidence that iron accumulates in several tissues with age [26–29], supports the prevailing opinion that age-dependent iron accumulation contributes to the aging process by increasing oxygen reactive species production, which over time causes extensive mitochondrial and cellular dysfunction [30]. Consistently, dietary iron supplementation reduces lifespan and accelerates age-dependent protein aggregation in *C. elegans* [31]. Instead, reduced iron absorption increases longevity in flies [32] and iron chelant therapy reduces oxidative stress in worms [33].

Iron responsive proteins (IRP1 and IRP2) and their binding sites in mRNAs (iron responsive elements, IREs) constitute an iron sensing system that tightly regulates intracellular iron homeostasis by post-transcriptional regulation of key genes involved in iron uptake, storage, export and utilization [34], thus strongly influencing the energy production and redox status of cells. IRP2 is particularly enriched in the CNS and it appears to play a preeminent role in the regulation of intracellular iron homeostasis within the CNS [35]. Indeed, alteration of IRP2 alone, but not of IRP1 alone, induces iron imbalance and iron-mediated toxicity in neurons [35, 36]. Despite the central role of IRPs in regulating iron homeostasis, little is known as to their regulation during aging and whether their altered expression contributes to the age-related decline. It is known, however, that downregulation of transferring receptor, a direct target of IRPs, is among the most robust transcriptional markers of aging [37] (and personal unpublished observations).

Here, we investigated the role of miR-29 during adult life in the short-lived turquoise killifish *Nothobranchius furzeri﻿﻿ (turquoise killifish﻿﻿*). This is the shortest-lived vertebrate that can be cultured in captivity and has emerged as model organism to study vertebrate aging. Its aging is characterized by an exceptionally short lifespan of 4–12 months (depending on genetic background), age-dependent cognitive decline, and expression of age-related phenotypes at the molecular, cellular, and integrated level [38–44] (for a review see [45]). In particular, genome-wide transcript regulation patterns during aging are similar in *N. furzeri* and humans [46] and miR-29 is upregulated during *N. furzeri* aging [11]. It therefore represents an ideal model to investigate how a perturbation of miR-29 expression affects aging. We generated a transgenic line with neuronal specific mir-29 loss of function and analyzed genome-wide transcript regulation by RNA-seq to compare the perturbations induced by miR-29 loss with those induced by aging.

## Results

### MiR-29 is up-regulated with age in neurons and is negatively-correlated with expression of its targets

**Fig. 1:**
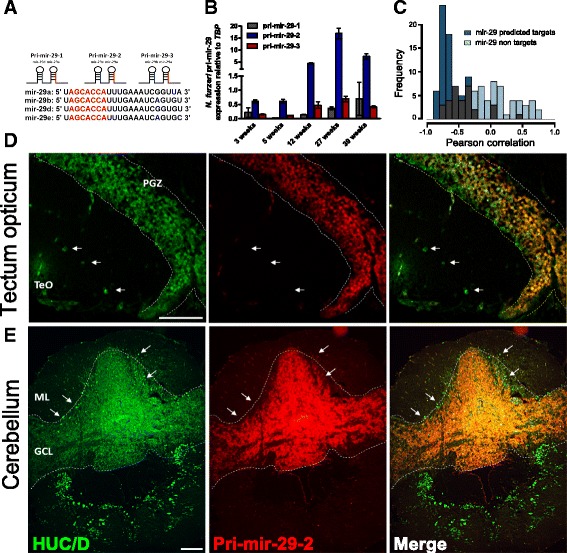
MiR-29 is up-regulated with age in neurons. **a** Genomic organization of miR-29 family in *N. furzeri*. Three different clusters were isolated (pri-mir-29 1, 2, 3) encoding four different mature members miR-29a, b, d, e. In red, seed sequence is reported, single nucleotide differences in blue. **b** Age-dependent expression of miR-29 primary transcripts (Pri-miR-29-1, 2, 3) in the brain of *N. furzeri*. The relative expression was evaluated by RT-qPCR, data were normalized on TATA binding protein (TBP), pri-miR-29-2 results much more expressed than the other clusters and shows a clear age-dependent up-regulation (1 way ANOVA with post-test for trend: R = 0.5285 *P* < 0.0001, n = 4 animals for age group). **c** Correlation of miR-29 with its predicted targets. Blue bars show the distribution of Pearson’s correlation coefficients between miR-29a and its predicted target. Light-blue bars show the distribution of correlation values extracted from a bootstrap (*P* = 10^–14^, Kolmogorov–Smirnoff). **d**, **e** Pri-miR-29-2 expression pattern in *N. furzeri* brain. **d** Pri-miR-29-2 signal (red) and HuC/D expression (green) in the optic tectum (TeO). Pri-miR-29-2 shows a nuclear staining and a co-localization with neuronal marker HuC/D along the periventricular gray zone (PGZ), white arrows show neurons in the optic tectum (TeO) negative for pri-miR-29-2. Scale bar = 50 μm. Cerebellum overview picture (**e**) shows a clear and strong expression of pri-miR-29-2 just in the granular cell layer (GCL), it is instead absent in the Purkinje cell (white arrow) and molecular layer (ML). Scale bar 100 μm

We searched the killifish genome [47] for miR-29 members and found three distinct mir-29 genomic clusters referred to as pri-mir-29 1, 2, 3 (Fig. 1a). Each cluster contains two miR-29 mature members, miR-29a is contained in all clusters paired with miR-29b or either of two novel miR-29 family members that we provisionally refer to as miR-29d and miR-29e (Fig. 1a). All of these miRNAs share the same seed sequence, but differ in their 3′ ends. Thus, we tested whether all three clusters exhibit an age-dependent transcriptional regulation by RT-qPCR. We found that all clusters are actively transcribed; however, one is expressed at much higher level (pri-mir-29-2) and also shows the largest age-dependent up-regulation, increasing its expression more than 15 times between 3 and 27 weeks of age (Fig. 1b). To assess the impact of miR-29 on global age-dependent gene expression, we took advantage of combined RNA-seq and miRNA-seq data from *N. furzeri* brain at five ages [46] and considered all protein-coding genes with rapid age-dependent decay in expression (31% of all differentially expressed genes, see [46]). This gene set was enriched for genes containing a miR-29 binding site as predicted by MiRanda (false discovery rate (FDR) < 0.05, Fisher’s exact test). In addition, miR-29 targets showed an excess of negative correlation with miR-29 expression as opposed to non-targets (*P* < 10^–13^, Kolmogorov–Smirnoff, Fig. 1c). These data suggest that miR-29 is a regulator of age-dependent gene expression, as previously reported in the prefrontal cortex of humans and macaques using similar methods [5]. Since one mir-29 primary transcript shows much higher expression than the other two, we focused our investigation on this cluster and used in situ hybridization to define its expression domains in the adult *N. furzeri* brain. It is intensely expressed in the periventricular gray zone of the optic tectum (Fig. 1d) and in the granular cell layer of cerebellum (Fig. 1e), where it is co-localized with the neuronal marker HuC/D, thereby confirming the neuronal expression of miR-29 originally reported in the mouse [23].

### Genetic repression of mir-29

**Fig. 2:**
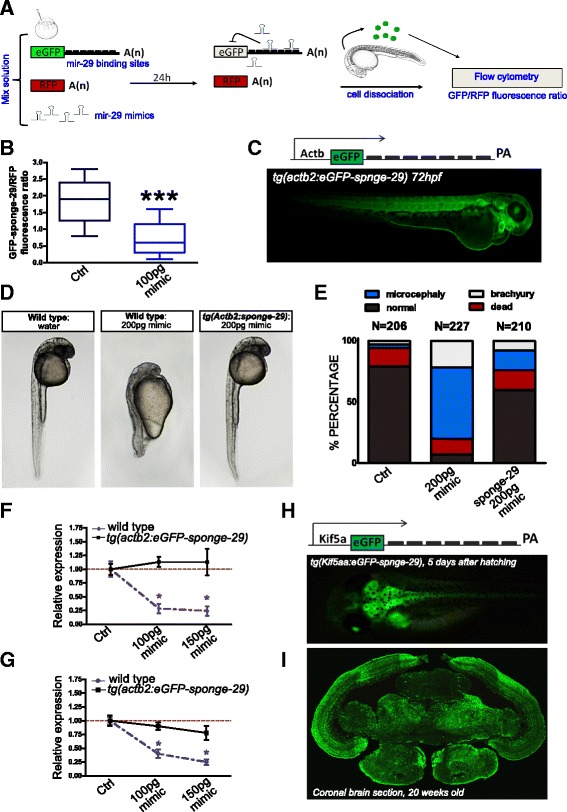
Generation of mir-29 loss of function *N. furzeri*. **a** Scheme of the reporter assay for miR-29 activity. Green fluorescent protein (GFP) mRNA carrying multiple miR-29 binding sites is injected in one-cell stage zebrafish embryos along with red fluorescent protein (RFP) mRNA, as a loading control, and miR-29 mimics. Control embryos are injected with same mix without miR-29 mimics. Binding of miR-29 mimics to reporter mRNA causes repression of GFP signal. At 24 hours post injection embryos are dissociated (n = 30–40) and cells relative fluorescence is read by flow cytofluorescence. **b** Fluorescence cell analysis. MiR-29 mimics strongly reduced eGFP-sponge signal (***P < 0.001, T-test). Whisker plots indicate the 10%, 25%, median, 75%, and 90% ranges. **c** On the top the expression cassette, consisting of 5.3 kb of zebrafish actb2 promoter, eGFP fused to a synthetic 3′-UTR containing seven repetitions of miR-29 binding site, and a SV40 late poly-A tail is reported. A *tg(actb2:eGFP-sponge-29)* F1 embryo generated by Tol2-mediated transgenesis is shown at the bottom. **d**, **e** Wild-type and F1 transgenic zebrafish were injected with 200 pg of miR-29a or miR-29b mimics at the one cell stage, control embryos were injected with water and red phenol only. Picture (**d**) shows representative control embryos, wild-type embryos +200 pg mimics, transgenic embryos + 200 pg mimics at 24 hpf. Stacked bar chart (**e**) represent the percentages of embryos with different phenotypes (normal, death, microcephaly, and brachyury) in the three conditions. **f**, **g** Expression level at 24 hpf of *Col11a1*, *Elna*, *Ireb2*, and *Tfr1a* upon miR-29 mimic injections. One-stage wild-type and sponge-29 line were injected with different doses (100 and 150 pg) of microRNA mimics and the expression level determined by RT-qPCR. Statistical significance was assessed by one-way ANOVA with post-hoc Tukey’s test (*P < 0.05), the analysis was performed on total RNA extraction from 30–40 embryos for each condition. Error bars indicate standard errors of means. **h** Schematic representation of sponge expression cassette driven by 3.1 kb of kif5aa promoter and the *tg(kif5aa:eGFP-sponge-29)* f1 killifish embryo, 5 days after hatching. **i** eGFP immunodetection on *tg(kif5aa:Egfp-sponge-29)* 20-week-old killifish brain section

We generated transgenic zebrafish and *N. furzeri* overexpressing a competitive miR-29 inhibitor and evaluated the global effects of miR-29 antagonism on gene expression in adult life. We used a well-established strategy by fusing to the eGFP CDS a 3′-UTR bearing seven repetitions of a miR-29 binding site to generate a transcript that scavenges endogenous miR-29 (miR-29-sponge). As a first step, we tested whether miR-29-sponge mRNA was directly targeted by miR-29 family members by co-injecting its mRNA along with RFP control mRNA and miR-29 mimics into zebrafish embryos. At 24 hours post-fertilization (hpf), injected embryos were dissociated and the relative eGFP/RFP fluorescent ratio in dissociated cells was measured by fluorocytometry (according to [48]) (Fig. 2a). A strong repression of eGFP signal was induced by miR-29 mimics (Fig. 2b). We further generated a transgenic zebrafish line where miR-29-sponge expression was controlled by 5.3 kb of *D. rerio* actin beta 2 promoter *tg(actb2:eGFP-sponge-29)* (Fig. 2c) to evaluate the biological activity of sponge-29 in vivo. For this purpose, we took advantage of the extremely low expression of miR-29 during the first 2 days of embryonic development [49] and injected increasing doses of miR-29a or miR-29b mimics (~100, ~150, and ~200 pg) both in wild-type and in *tg(actb2:eGFP-sponge-29)* zebrafish embryos. Both mimics induced similar effects in wild-type embryos and 200 pg of mimic were sufficient to induce overt morphological defects like brachyury, microcephaly, and microphthalmia. The percentage of defective embryos induced by miR-29 mimics was much lower in the *tg(actb2:eGFP-sponge-29)* line (Fig. 2d, e). To assess the effects of sponge-29 on gene regulation, we injected doses that were not inducing morphological defects (100 and 150 pg of mimics) and analyzed two well-validated miR-29 targets: *Elna1* (elastin) and *Col11a1a* (collagen type XI, alpha 1a), both highly expressed in embryos and bearing in their 3′-UTR four and two predicted target binding sites, respectively (Additional file 1). We observed a dose-dependent down-regulation of these transcripts in wild-type, but not in *tg(actb2:eGFP-sponge-29)* injected embryos (Fig. 2f, g). All these data support the notion that genetically modified fish with stable sponge-29 overexpression counteract miR-29-induced gene regulation in vivo. Since pri-miR-29-2 is mainly expressed in neurons, we decided to antagonize miR-29 activity specifically in neuronal cells and evaluate the effects on global gene expression. To this end, we isolated from zebrafish genomic DNA a 3.1 kb fragment of the neuronal specific promoter Dre-kif5aa (kinesin 5aa), thus generating the transgenic line *tg(kif5aa:eGFP-sponge-29)*. This kif5aa promoter fragment was sufficient to maintain the expression of eGFP-sponge-29 throughout life both in zebrafish and *N. furzeri*. Since zebrafish life expectancy is about 5 years [50], we decided to generate a stable line in the short-lived turquoise killifish in order to more rapidly investigate the consequences of miR-29 depletion during adult life. To this end, we used the MZM-0410 strain with a median lifespan of approximately 7 months [51]. *N. furzeri* F1 fish exhibited a stable expression over time (Fig. 2h, i) and a double labeling with eGFP and HuC/D proved the neuronal specificity of the expression pattern (Additional file 2). We did not observe any macroscopic defect, but did observe a reduced fertility and reduced post hatch survival, which limited the number of animals that could be analyzed. It should be noted that a full knock-out of miR-29 is postnatally lethal in the mouse [17], so reduced viability is expected and the fact that some transgenic fish reached adulthood could be due to the incomplete antagonism of miR-29 by sponge-29.

### MiR-29 antagonism mimics aging-induced gene regulation in *N. furzeri* brain

**Fig. 3:**
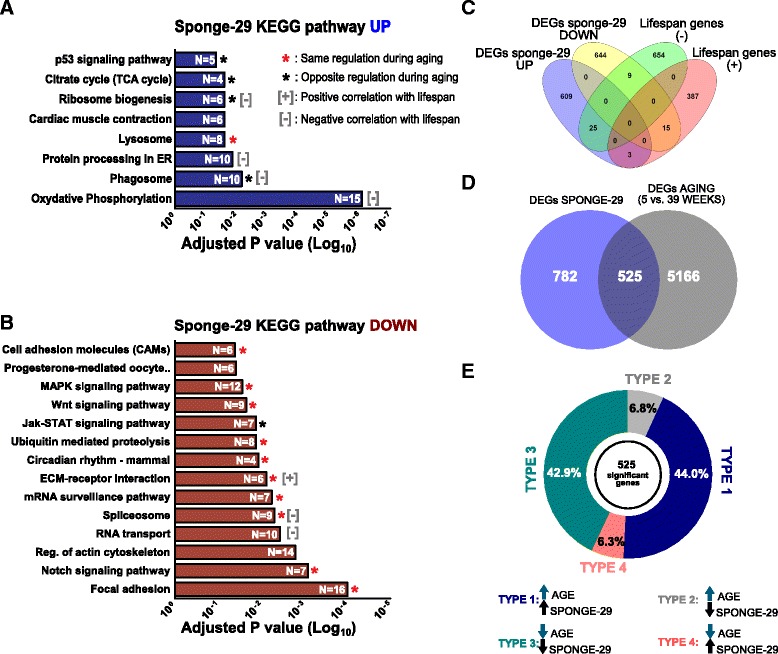
MiR-29 antagonism partially recapitulates aging. **a, b** KEGG pathway overrepresentation analysis on DEGs (FDR ≤ 0.05). The red and black asterisks indicate categories of DEGs regulated with age in the brain (from [47]). Red colored asterisk indicates categories that exhibit the same direction of regulation in both aging and *tg(kif5aa:eGFP-sponge-29)*. Black colored asterisk indicates those with opposite regulation. Gray plus and minus symbols represent categories that positively and negatively correlates with lifespan respectively (from [52]) **c** Venn diagram illustrating the intersection between up and downregulated DEGs in sponge-29 and genes positively and negatively correlated with lifespan, respectively (*P* = 0.0007 and *P* = 0.0049, Fisher exact test). **d** Venn diagram illustrating the intersection between DEGs during aging and DEGs in sponge-29. **e** Correlation analysis of fold-changes of genes in the intersection shown in (**d**). Pie chart represents the fraction of genes coherently or incoherently regulated by aging and sponge-29

We analyzed the global gene expression profile in F1 sponge-29 transgenic fish through RNA-seq. Our previous RNA-seq analysis of transcriptome regulation during aging in the turquoise killifish brain showed that the most relevant change in global gene expression occurs between 5 and 12 weeks [46]. Here, we decided to compare the expression profile of 12-week-old transgenic fish with 12-week-old wild-type fish, using four biological replicates for the experimental group. We detected 1305 differentially-expressed genes (DEGs, FDR < 0.05, edgeR, Additional file 3). KEGG pathway analysis revealed an over-representation among up-regulated genes of terms involved in energy production processes such as oxidative phosphorylation and TCA cycle as well as ribosome biogenesis, lysosome, phagosome, and endoplasmic reticulum functions and downstream p53 effectors (Fig. 3a) and an overrepresentation among down-regulated DEGs for terms involved in biosynthesis and catabolism of biomolecules, extracellular remodeling, circadian rhythm and the following pathways: JAK-STAT, MAPK, WNT, and Notch (Fig. 3b, complete KEGG pathway analysis is reported in Additional file 4). Several of these pathways were previously reported to be regulated in the *N. furzeri* brain during aging (see [46, 47]). Remarkably, the majority of these pathways showed the same direction of regulation during aging and in response to miR-29 antagonism (red asterisk, Fig. 3a, b) and only a minority showed opposite directions of regulation (black asterisk, Fig. 3a, b). Moreover, in a longitudinal study of *N. furzeri*, Baumgart et al. [52] reported that a higher expression of genes belonging to the oxidative phosphorylation, phagosome, lysosome, ribosome biogenesis, and RNA transport pathways is negatively correlated with lifespan. On the other hand, higher expression of extracellular matrix-receptor interaction genes is positively correlated with lifespan [52]. Therefore, our results indicate that miR-29 antagonism induces an up-regulation of those pathways negatively correlated with lifespan (marked with a grey minus in Fig. 3a, b), with the exception of RNA transport and spliceosome pathways and a down-regulation of the only pathway positively correlated with lifespan (marked with a plus in Fig. 3a, b). Among up regulated DEGs, we found 25 genes negatively correlated and 3 positively correlated with lifespan (Fig. 3c); among down-regulated DEGs, we found 9 genes negatively correlated and 15 positively correlated with lifespan (Fig. 3c). We interpret these data as a signature of accelerated aging. To corroborate our hypothesis, we compared the effects of aging and miR-29 antagonism at the individual gene level. We intersected the sets of DEGs of sponge-29 transgenic fish and aging (obtained by comparing gene expression of 5- vs. 39-week-old fish; [46]). The intersection contains 525 genes (Fig. 3d) and 456 (~87%) show the same direction of regulation in aging and miR-29 sponge (*P* < 10^–16^, χ^2^ test). Of those, 225 are up-regulated (Type 1 genes, Fig. 3e, Additional file 5) and 231 are down-regulated (type 3 genes, Fig. 3e, Additional file 5) in both conditions (aging and miR-29 antagonism). Only 68 genes (~13%) showed opposite regulation: 34 down-regulated in sponge-29 and up-regulated during aging (type 2, Fig. 3e, Additional file 5) and 34 with the opposite behavior (type 4, Fig. 3e, Additional file 5). Furthermore, we assessed the expression profile of genes with a conserved predicted binding site for miR-29. To this end, we retrieved a list of *D. rerio* 548 predicted targets (score ≤ –0.30) from TargetScanFish 6.2 [53]. Out of those, 38 genes were DEGs in kif5a: sponge-29 *N. furzeri* fish; 28 up-regulated and 10 down-regulated (Additional file 6a). A total of 22/28 *N. furzeri* ortholog up-regulated genes exhibited a conserved binding site (Additional file 6b). For 10/28, the miR-29 predicted binding site was conserved also in the mouse and human orthologs and for 5 of these an interaction with miR-29 was observed also by cross-linking immunoprecipitation (CLIP)-seq (Additional file 6b). Among these, we found genes involved in epigenetic reprogramming (Lysine-Specific Demethylase 6B (KDM6BB), DOT1-like, histone H3 methyltransferase (DOT1L), Enhancer of polycomb homolog 1 (EPC1)), glutamate metabolism (glutamate receptor-interacting protein 1 (GRIP1)), and IGF1 signaling (insulin-like growth factor binding protein 2a (IGFBP2A)), consistent with the known biological actions of miR-29 reported in the literature. Curiously, among the down-regulated DEGs, we found well-known mammalian miR-29 targets such as Ten-eleven translocation methylcytosine dioxygenase 3 (TET3), DNA (cytosine-5)-methyltransferase 3ab (DNMT3AB), and collagen type IV alpha 1 (COL4A1) (Additional file 6c). Thus, we examined the effect of aging on conserved miR-29 predicted targets. Of 22 up-regulated targets, 6/22 are type 4 genes (Fig. 3e) and 12/22 were not regulated by aging, indicating that those miR-29 could directly influence their expression by regulating transcript stability. On the other hand 4/22 up-regulated targets and 9/10 down-regulated targets were type 1 and type 3 genes (Fig. 3e, Additional file 6b, c), i.e., showed the same direction of regulation during aging and in response to miR-29 antagonism, indicating that, for these genes, the global effect of miR-29 depletion on aging-dependent gene expression is prevalent over the target-specific effect of miR-29.

### Mir-29 affects iron homeostasis via regulation of IRP2

**Fig. 4:**
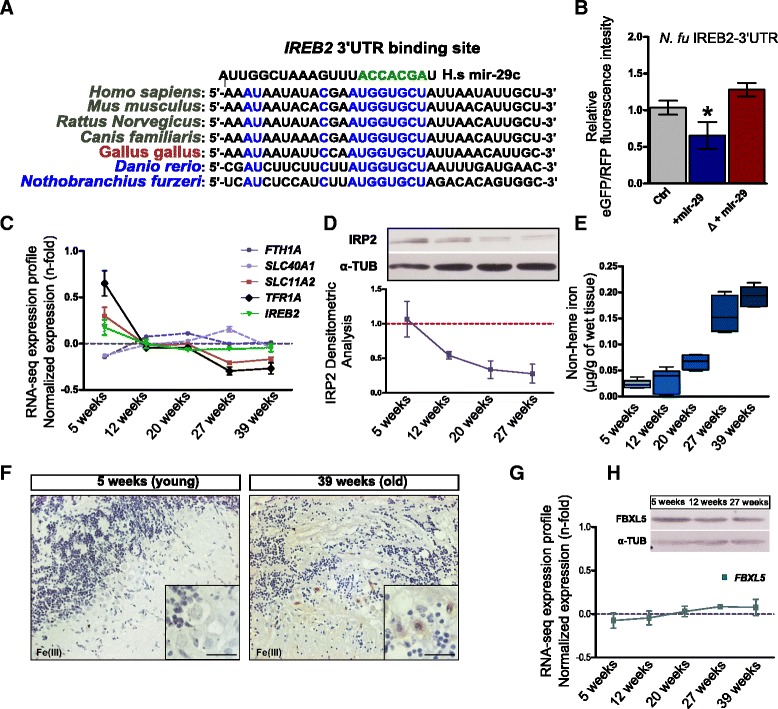
MiR-29 family targets *Ireb2* mRNA. **a** Presence of a putative binding site for miR-29 family in the *Ireb2* mRNA 3′-UTR sequence in several vertebrate species (*H. sapiens*: ENSG00000136381; *M. musculus*: ENSMUSG00000032293; *R. norvegicus*: ENSRNOG00000013271; *C. familiaris*: ENSCAFG00000001766; *G. gallus*: ENSGALG00000003171; *D. rerio*: ENSDARG00000021466; *N. furzeri*: Nofu_GRZ_cDNA_3_0193494), mammalian sequences are in black, birds in purple, and teleost fish in blue. Perfect match to positions 2–8 of the miR-29 seed sequence is highlighted in green and is present in all vertebrate sequences shown. **b** Expression of GFP assessed by cytofluorometric analysis. Fusion with *Ireb2* 3′-UTR of *N. furzeri*. The gray column reports the baseline fluorescence intensity of the construct without miR-29 mimic. The middle blue column reports the fluorescence with miR-29 mimic and the red column the fluorescence of a construct (Δ) where the putative binding site for miR-29 in the *Ireb2* 3′-UTR was mutated to destroy complementarity. Statistical significance of fluorescence difference between baseline and co-injection with miR-29 mimics was evaluated by Student’s t-test (* *P* < 0.05). **c** Age-dependent regulation of transcripts coding for key genes of iron metabolism, from Baumgart et al. [46]. One-way ANOVA for linear trend is reported for each gene: *Tfr1a* (R = 0.5009; *P* < 0.0001), *Scl11a2* (R = 0.3892; *P* < 0.01), *Fth1a* (R = 0.067; *P* = 0.11), *Slc40a1* (R = 0.078; *P* = 0.11), *Ireb2* (R = 0.141; *P* = 0.069). Expression values (in RPMKs) for each age were centered and scaled to the mean, n = 5 animals for age point. **d** Representative Western blot of IRP2 in brain extracts and densitometric analysis relative to Additional file 8a (Kruskal–Wallis test, *P* = 0.0216, *n* = 3 animal for each age point). α-TUBULIN was used as loading control. **e** Brain non-heme iron content (μg/g wet tissue) in fish of different ages. (One-way ANOVA: *P* < 0.0001). **f** DAB-enhanced Perls staining of the cerebellum of young and old fish. The black arrows point to labelled Purkinje cells. The inset in the bottom right corner of each picture shows a magnification of the Purkinje cell bodies. **g**, **h** Age-dependent expression of *Fbxl5* mRNA (ANOVA for linear trend: R = 0.1922, *P* = 0.0688) and western blot of FBXL5 in brain lysates at three age points (5, 12, and 27 weeks). α-TUB was used as loading control. For **b**, **c**, **d**, **e**, **g** mean ± standard errors of means is reported

**Fig. 5:**
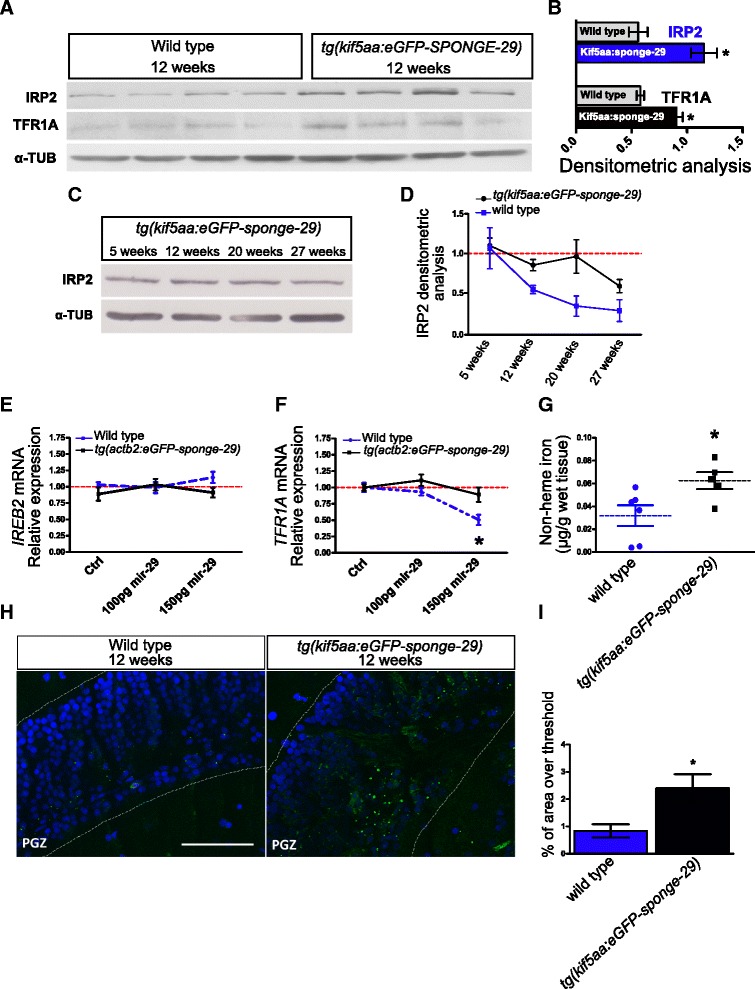
Genetic repression of miR-29 chronically affects iron homeostasis. **a**, **b** Western blot of IRP2 and TFR1A in 12-week-old *tg(kif5aa:eGFP-sponge-29)* (*n* = 4) and wild-type (*n* = 4) fish brain extracts and (**b**) relative densitometric analysis (*P < 0.05; **P < 0.01, Mann–Whitney U-test). **c** Representative Western blot of IRP2 in brain extracts of kif5a:sponge-29 at different ages (5, 12, 20, 27). In a–c α-TUBULIN was used as loading control. **d** Comparison of densitometric analysis calculated on Additional file 8a and b. The blue line represents IRP2 expression in *tg(kif5aa:eGFP-sponge-29)* animals, gray line represents expression in wild-type fish. Values were normalized to the mean of 5 weeks value (Kruskal–Wallis test, *P* = 0.0939, *n* = 3 animals for each age point). **e, f** Expression level at 24 hpf of *Ireb2* and *Tfr1a* upon miR-29 mimics injection. The expression level was determined by RT-qPCR. Statistical significance was assessed by one-way ANOVA with post-hoc Tukey’s test (*P < 0.05), the analysis was performed on total RNA extraction from 30–40 embryos for each condition. Error bars indicate standard errors of means. **g** Brain non-heme iron content (μg/g wet tissue) in kif5a:eGFP-sponge-29 (*n* = 5) compared to wild-type (*n* = 6) at age 12 weeks (*P < 0.05, Mann–Whitney U-test). **h** Representative images of lipofuscin accumulation in the optic tectum of 12-week-old kif5a:eGFP-sponge-29 and wild-type fish brains. Lipofuscin auto-fluorescent granules (green) were detected with ApoTome microscope, counterstained with DAPI (blue). Scale bar: 50 μm. **i** Quantification of lipofuscin density based on percentage of area over threshold, n = 6 (*P < 0.05; Mann–Whitney U-test)

The observed transcriptomic signature of accelerated brain ageing induced by miR-29 antagonism is likely due to dysregulation of multiple pathways. However, KEGG pathway analysis retrieved a consistent overrepresentation of terms related to energy production processes, in particular mitochondria processes such as oxidative phosphorylation and TCA cycle. Further, among type-4 genes, i.e., genes normally downregulated during aging but up-regulated in sponge-29 transgenic fishes, we noticed genes that have a key-role in the regulation of iron homeostasis or heme synthesis such as transferrin (*TF*), transferrin receptor (*TFR1A*), the ferroxidase ceruloplasmin (*CP*), and the heme transporter *SCL22A4*. Since iron is a key-molecule for mitochondria activity and energy production and iron imbalances have profound implications in the aging-induced dysfunctions and lifespan extension [31, 54, 55], we supposed that this specific process may contribute to the accelerated aging phenotype observed upon miR-29 loss of function. The dysregulation of *TFR1A* is particularly interesting for at least three different reasons: (1) TRF1A is the primary carrier responsible for iron import, (2) its expression is much higher in neurons than in glial cells in vivo [16], and (3) a meta-analysis of microarray studies identified *TFRC* (the mammalian orthologous of *TFR1A*) as the gene with the most robust age-dependent downregulation across multiple tissues and mammalian species and it is considered as part of a common transcriptional signature of aging [37]. We therefore investigated the presence of possible miR-29 binding sites in mRNAs coding for genes involved in iron homeostasis and noticed the presence of an 8 nt seed sequence in the 3′-UTR of the Iron-Responsive Element Binding Protein 2 (*Ireb2*) mRNA that is conserved across vertebrates (Fig. 4a). Accordingly, both miRanda [56] and TargetScan [57] retrieved *Ireb2* mRNA as a target for miR-29 in both fishes and mammals. In addition, StarBase supports a physical interaction of miR-29 and *Ireb2* 3′-UTR in human and mouse [58]. The *Ireb2* gene encodes for the protein IRP2 that, along with IRP1, is considered a master regulator of intracellular iron homeostasis. To demonstrate a direct regulation of *Ireb2* mRNA by miR-29 in fishes, we isolated the 3′-UTRs of *Ireb2* from zebrafish and *N. furzeri* cDNA and fused either of them with the eGFP coding sequence to generate a reporter construct; 100 pg of each reporter mRNA were co-injected in fertilized zebrafish embryos in combination with the same amount of control mRNA encoding for the red fluorescence protein (RFP) and 100 pg of dre-miR-29a or dre-miR-29b mimic. Control embryos were injected with the same mixture omitting the miRNA mimics. At 24 hpf, 30–40 injected embryos were digested and cell fluorescence was analyzed by cytofluorimetry to assess the relative eGFP/RFP fluorescence ratio. For both 3′-UTRs, the normalized eGFP fluorescence was significantly reduced in the presence of miR-29 mimics (Fig. 4b, Additional file 7). For both constructs, a mutation of three nucleotides in the putative target sequence of the 3′-UTRs abolished repression (Fig. 4b, Additional file 7). In addition, we performed a luciferase assay in the human HEK293T cell line to demonstrate direct targeting of *Ireb2* 3′-UTR by miR-29 in mammals. MiR-29 decreased luciferase activity of a reporter containing mouse *Ireb2* 3′-UTR and repression was abrogated by mutation of three nucleotides in the putative binding site (Additional file 7). Given the direct interaction between miR-29 and *Ireb2*, we tested whether *Ireb2* expression is reduced during aging. The variations in *Ireb2* expression are not significant at the level of mRNA, but are significant at the protein level (ANOVA for trend, Fig. 4c, d, see Additional file 8a for the individual blots). To investigate the impact of IPR2 downregulation, we analyzed the age-dependent expression of mRNAs coding for its direct targets: *Tfr1a*, *Scl11a2*, *Fth1a*, and *Slc40a1* (Fig. 4b). Consistently with IRP2 downregulation, expression of transferrin receptor (*Tfr1a*) and the solute carrier transporter 11a2 (*Slc11a2*) gene, directly involved in intracellular iron delivery, decreased with age (Fig. 4c). On the other hand, transcripts coding for ferritin heavy chain (*Fth1a*) and Ferroportin (*Slc40a1*), genes required for iron storage and discharge, were not significantly regulated (*P* > 0.05, ANOVA for trend and quadratic fitting Fig. 4c). Specific age-dependent downregulation of intracellular iron delivery genes is likely a direct result of iron accumulation, as the same specific regulation was observed in mammals [59]. To directly demonstrate iron accumulation during normal aging, we quantified brain non-heme iron concentration using a colorimetric assay [60] in fish of five age groups: at 5, 12, 20, 27 and 39 weeks. These age steps correspond to sexual maturity (5 weeks), young adult stage (12, 20 weeks), median lifespan (27 weeks), and exceptional survivors (39 weeks). From 5 to 39 weeks of age, non-heme iron amount increases almost 10-fold in fish brains (Fig. 4e). This datum was corroborated by histochemical Pearl’s staining with DAB intensification on brain sections. Increased labelling was apparent both in gray and white matter of 39-week-old fish (Fig. 4f). In mammals, iron loading negatively regulates IRP2 by inducing cytosolic FBXL5 accumulation that in turn induces IRP2 proteasomal degradation. Since iron accumulates during aging, we would expect to observe an age-dependent FBXL5 up-regulation along with IRP2 reduction but neither *Fbxl5* mRNA nor its protein were significantly modulated by age (Fig. 4g, h). Thus, the observed age-dependent downregulation of IRP2 cannot be mediated by FBXL5, but could be mediated by miR-29. We therefore quantified the expression of IRP2 and TFR1A in brains of wild-type and *tg(kif5aa:eGFPsponge-29)* fish at young adult (12-week-old) stage. Both proteins were found up-regulated in sponge-29 lines compared to control animals (Fig. 5a, b). In addition, we analyzed the regulation of IRP2 over time in fish overexpressing mir-29 sponge and found no significant reduction over time as opposed to the clear age-dependent regulation observed in wild-type fish (compare Fig. 4d with Fig. 5c, d, original blots are reproduced in Additional file 8b). Finally, we tested in zebrafish embryos whether miR-29 overexpression represses *Tfr1a* expression by microinjecting100 and 150 pg of miR-29 mimic in wild-type and *tg(actb2:eGFP-sponge-29)* embryos. No significant *Ireb2* mRNA downregulation was observed (Fig. 5e), but a significant reduction of *Tfr1a* mRNA was observed in wild-type embryos but not in *tg(actb2:eGFP-sponge-29)* embryos (Fig. 5f).

Overall, these data strongly indicate that miR-29 is responsible for age-dependent downregulation of IRP2 in neurons that, in turn, reduces TFR1A expression. As a proof of principle that miR-29 could exhibit a conserved regulation of iron homeostasis also in mammals, we evaluated the expression of miR-29 and iron management genes during post-natal development in the mouse brain. A dramatic up-regulation of mature miR-29 members during the first 2 months of postnatal life was already described [16, 23]. We tested whether miR-29 increase correlates with *Ireb2* and *Tfr1a* downregulation. Using RT-qPCR, we quantified the expression of both miR-29 primary transcripts in postnatal day 0 (P0) and P60 mouse cerebral cortex. Both increased their expression more than 30 times (Additional file 9a) while *Ireb2*, *Tfr1a*, and *Scl11a2/DMT1* were significantly downregulated (Additional file 9b). In addition, during the preparation of this manuscript, Papadopulous et al. [18] reported that mice lacking one locus for miR-29 show up-regulation of *Ireb2*, lending further support to a direct regulation of IRP2 expression by miR-29 in vivo.

Neuronal-specific overexpression of IRP2 leads to mitochondria damage and pervasive oxidative stress [61]. Therefore, we assessed iron content and histochemical markers of damage in the sponge-29 transgenic line. Non-heme iron content was significantly increased as compared to wild-types at age 12 weeks (Fig. 5g). To assess whether increased iron induced oxidative stress we analyzed lipofuscin, an autofluorescent pigment that is primarily composed of cross-linked protein residues, generated by iron-catalyzed oxidative processes [62]. Lipofuscin deposition was quantified in the periventricular grey zone of the optic tectum, a brain region composed by tightly packed neuronal somata and that shows a strong expression of miR-29 (Fig. 1d) and was significantly increased in the sponge-29 line as compared to wild-type (Fig. 5h). Finally, gliosis was assessed as a nonspecific reactive change of glial cells in response to neuronal damage. Increased immunoreactivity for the glial markers GFAP and S100β was observed (Additional file 10).

Our data indicate that miR-29 upregulation with age reduces IRP2-TFR1A axis in neurons thereby reducing intracellular iron delivery and limiting iron-mediated neuronal damage.

### Iron loading induces miR-29 in neurons

**Fig. 6:**
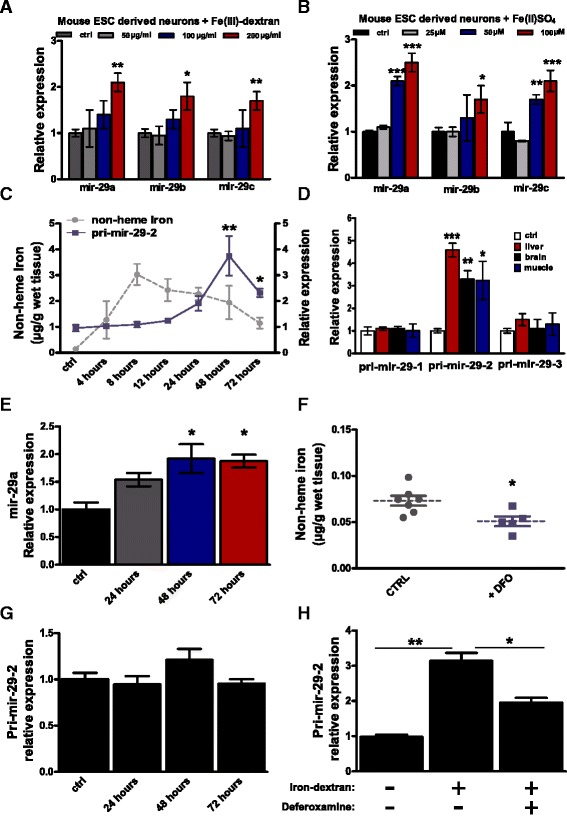
Iron overload induces miR-29 up-regulation in neurons and brain. **a**, **b** Modulation of miR-29 family members in murine neurons derived from mESCs incubated respectively with **a** 0, 50, 100, or 200 μg/mL of Fe(III)-dextran or with **b** 0, 25, 50, or 100 μM of Fe(II)SO_4_ for 72 hours (* *P* < 0.05; **P < 0.01; ***P < 0.001, one-way ANOVA with post-hoc Tukey’s test), *n* = 3 independent experiments performed in duplicates for each condition. **c** Time course of non-heme iron amount (quantified by colorimetric analysis) and pri-miR-29-2 expression level in fish brain (quantified by RT-qPCR) following intraperitoneal (i.p.) injection of 350 μg/g of iron dextran, control animals were injected with saline solution. Grey line represents iron amount, blue line represents pri-miR-29-2 relative expression level (setting the baseline to 1, *P < 0.05; **P < 0.01; one-way ANOVA with post-hoc Tukey’s test), *n* = 4 biological replicates for each point. **d** Relative expression of miR-29 primary transcripts (pri-mir-29-1, 2, 3) in liver, brain and muscle 48 hours following i.p. injection of 350 μg/g of iron dextran. Data were normalized on TBP expression and control animals relative expression was considered as baseline (***P < 0.001; **P < 0.01; *P < 0.05; Mann–Whitney U-test), *n* = 5 biological replicates. **e** Expression of mature N.fu-miR-29a at 48 hours after iron injection quantified by RT-qPCR (* *P* < 0.05; Mann–Whitney U-test), *n* = 4 biological replicates for each time point, U6 was used as normalization control. **f** Non-heme iron quantification 24 hours after deferoxamine (DFO) i.p. injection, control animals were injected with saline solution (*P < 0.05; Mann–Whitney U-test), *n* = 7 control animals and *n* = 5 DFO injected animals. **g** Time course of pri-miR-29-2 expression level (quantified by RT-qPCR) following i.p. injection of 30 μg/g of DFO, one-way ANOVA *P* = 0.1663, *n* = 4 biological replicates for each time point. **h** Modulation of pri-miR-29-2 expression in fish brain (quantified by RT-qPCR) after iron overload and in combination with administration of 30 μg/g of DFO, control animals were injected with saline solution (*P < 0.05; Mann–Whitney U-test), *n* = 5 biological replicates. For all the graphs, mean ± standard errors of means are reported

To study the effects of iron on expression of miR-29 in neurons we employed murine stem-cell derived telencephalic neurons in vitro [63]. Incubation of murine neurons with either Fe(II) or Fe(III)-dextran induced an up-regulation of miR-29 (Fig. 6a, b). We then induced acute iron overload in adult fish by parental injection of 350 μg per gram of weight of Fe-dextran and monitored brain iron concentration and miR-29 expression up to 3 days post-injection (pi). Iron significantly accumulated in the brain 4 hpi (the first time point investigated) reaching a maximum at 8–12 hpi to slowly decrease (Fig. 6c, Additional file 11). We observed a delayed and significant increase of both the prevalent miR-29 primary transcript and miR-29a mature form, following the injection, starting at 24 hpi and reaching its maximum at 48 hpi (Fig. 6c, e; Additional file 11). In addition, iron overload caused up-regulation of miR-29 also in liver and muscle (Fig. 6d), suggesting a systemic effect. We then asked whether physiological steady-state levels of iron influence miR-29 expression by injecting i.p. 30 μg/g of the iron chelant deferoxamine (DFO). At 24 hpi, brain iron amount was reduced by 25% (Fig. 6f), we did not aim for stronger reduction of iron since this can have serious negative consequences on physiology. We monitored pri-miR-29-2 expression at 24, 48 and 72 hours after DFO injection and did not detect any differences despite reduced iron levels (Fig. 6g, Additional file 11), suggesting that lowering iron below physiological levels does not regulate miR-29. Finally, we replicated the iron-overload experiment and, after 4 hours, administered DFO. Delayed chelation of iron significantly reduced pri-miR-29 up-regulation (Fig. 6h; Additional file 11).

Our results suggest that iron amount affects mir-29 expression; however, since the delay between mir-29 increases and iron-overload, we consider that intracellular events triggered by iron increase and not iron directly are responsible for up-regulation of miR-29.

## Discussion

Here, we report the first case of modulation of neuronal aging by a miRNA in a vertebrate species. Neuronal-specific miR-29 loss of function induces accelerated expression of aging phenotypes both at the global gene expression level and the histological level. Age-dependent up-regulation of miR-29 is conserved among different vertebrate species and different tissues. With our work, we show that, in fish, (1) miR-29 is expressed in mature neurons as previously shown in mice, (2) a robust negative correlation between expression of miR-29 and its targets is observed during adult life, as previously shown in primates [5], and (3) miR-29 loss of function has detrimental effects, as previously observed in mammals both in physiological [17, 18] and pathological contexts such as ischemia [20], cancer [64], thymus involution [65], and inflammatory response [66, 67]. Therefore, miR-29 upregulation appears to be an evolutionary conserved aging signature that provides an advantage in opposing aging-induced dysfunctions. This hypothesis is further supported by the observation that miR-29 is upregulated in a mouse model of progeria [14]. In addition, during killifish aging, miR-29 is upregulated also in liver, skin [11], and heart (own published data). Consistently, miR-29 is reported to be upregulated in several tissues in aging mammals as well [5, 12–15]. Thus, miR-29 upregulation appears to be a common aging mechanism across different tissues.

These data also lead to the general concept that part of age-dependent gene regulation is adaptive and protective. Indeed, a recent study showed that out of 29 conserved genes that are down-regulated with age in nematode worms, fish, and mice, 15 cause life extension when experimentally down-regulated in the worm [68] and it was previously shown that miR-34 is upregulated with age in fruit fly and its overexpression is life-extending while the knock-out is life-shortening [9].

Although we did not investigate the molecular mechanism underlying age-dependent miR-29 upregulation, here we observed that miR-29 loss of function leads to increased iron deposition in the adult killifish brain and that, in turn, acute iron loading induces miR-29 upregulation in brain, liver, and muscle. In this respect, it should be noticed that age-dependent iron accumulation was observed in several organisms such us *C. elegans*, *D. melanogaster* [69], mice [27], primates [70, 71], and humans [28]. In mammals, iron increase is observed in several tissues, such as brain, heart, muscle, liver, skin, kidney, and spleen, and it appears to be a robust aging hallmark. However, our data also demonstrate that miR-29 does not respond immediately to an acute increase iron concentrations, but with a delay of 2 days. We therefore speculate that age-dependent accumulation of iron-mediated damage might represent the stimulus that triggers miR-29 upregulation during aging.

Further, we report that miR-29 regulates IRP2 expression directly and antagonism of miR-29 abrogates age-dependent repression of IRP2 in neurons, which is very likely the cause of increased iron accumulation in the brain of the sponge-29 line. In fact, IRP2 is an RNA-binding protein that acts as master regulator of intracellular iron availability in the brain, influencing both the energy production and the redox status of the cell. Similarly to miR-29, IRP2 in enriched in the CNS. Within the CNS, different cell types (neurons, astrocytes, oligodendrocytes, and microglia) express iron management genes at different levels due to differences in their iron requirement and utilization. For instance, neurons express high levels of TFR1A and low levels of ferritin which, on the other hand, is strongly expressed in astrocytes and microglia [72]. Indeed, the neuronal-specific expression of TFR1A reflects their high-energy metabolic rate, while the relative low ferritin expression suggests that, as opposed to astrocytes, iron storage is negligible as compared to iron usage by mitochondria and other metabolic processes. Moreover, neuronal restricted expression of mitochondrial ferritin in the mammalian brain further supports this consideration [73]. In addition, in rodents, the highest neuronal iron consumption seems to occur during perinatal development and coincides with neuronal maturation and myelin synthesis [74, 75]; subsequently, neuronal iron demand decreases along with *Tfr1a* mRNA expression. Due to the low cytosolic iron storage capacity of neurons, a small increase in iron loading may result in mitochondrial iron accumulation and significant changes in the mitochondrial redox status. Thus, modulation of TFR1A expression is crucial for redox status homeostasis in neurons. In fact, our work revealed that miR-29 increases in aging reduce IRP2 and TFR1A levels, therefore limiting excessive intracellular iron delivery and protecting neurons from iron-mediated toxicity. Although IRP2 simultaneously regulates several iron-management genes, neuron-specific loss-of-function of miR-29 results in a significant modulation of *Tfr1a,* but not of cytosolic *Fth1a* nor of *Scl40a2*, suggesting that neurons might regulate iron homeostasis primarily through modulation of TFR1A. Since expressions of *Fth1a*, *Scl40a2*, and FBXL5 – all linked to cytosolic iron – were stable during aging, we speculate that iron might accumulate primarily in the mitochondria causing mitochondrial dysfunction and that this effect is exacerbated by mR-29 knockdown. Our hypothesis is supported by the up-regulation of energy production pathways such as oxidative phosphorylation and TCA cycle in the sponge-29 line and by the direct demonstration that IRP2 upregulation induces mitochondrial iron accumulation in human lung cells [76]. Moreover, La Vaute et al. [36] showed that IRP2-deficient mice exhibit neurodegeneration linked to cytosolic iron accumulation in gray and white matter. Deficiency of IRP2 increases the expression of the iron storage protein ferritin and reduces the expression of the transferrin receptor leading to functional iron deficiency and therefore neuronal death. The same team reported a markedly reduced activity of mitochondria complex I and II as a consequence of iron starvation [77], and Galy et al. [78] reported that correct mitochondria iron supply requires IRP2 in mouse liver. All these data indicate that an important function of IRP2 is to control mitochondrial iron homoeostasis, thereby preventing iron starvation. Our work integrates this view, identifying a novel mechanism of IRP2 regulation, mediated by miR-29 that contributes to the prevention of iron-induced mitochondrial dysfunction.

A direct relation between aging and iron homeostasis was reported also by Klang et al. [31], showing that dietary iron supplementation significantly accelerates the onset of aging-related phenotypes, reduces the lifespan expectancy and increases the age-dependent protein aggregation in *﻿C. elegans﻿*. Strikingly, TCA cycle and oxidative phosphorylation proteins are the most enriched proteins in the iron-induced aggregates. Conversely, the sponge-29 lines exhibited up-regulation of the same categories at the transcript level. Overall, these data suggest two important considerations: (1) the transcriptional up-regulation of TCA cycle and oxidative phosphorylation proteins in response to miR-29 loss-of-function represents a compensatory response to iron-induced damage of mitochondrial protein. Up-regulation of these categories is therefore a marker of compromised mitochondrial functionality and of accelerated aging. Consistently, Baumgart et al. [52] showed that increased expression of oxidative phosphorylation genes (in particular those of complex I) negatively correlates with killifish lifespan. (2) Iron accumulation compromises mitochondrial activity, corroborating the observation that age-dependent iron accumulation occurs primarily in the mitochondria and that miR-29 might promote mitochondrial integrity through regulation of iron homeostasis.

## Conclusion

**Fig. 7:**
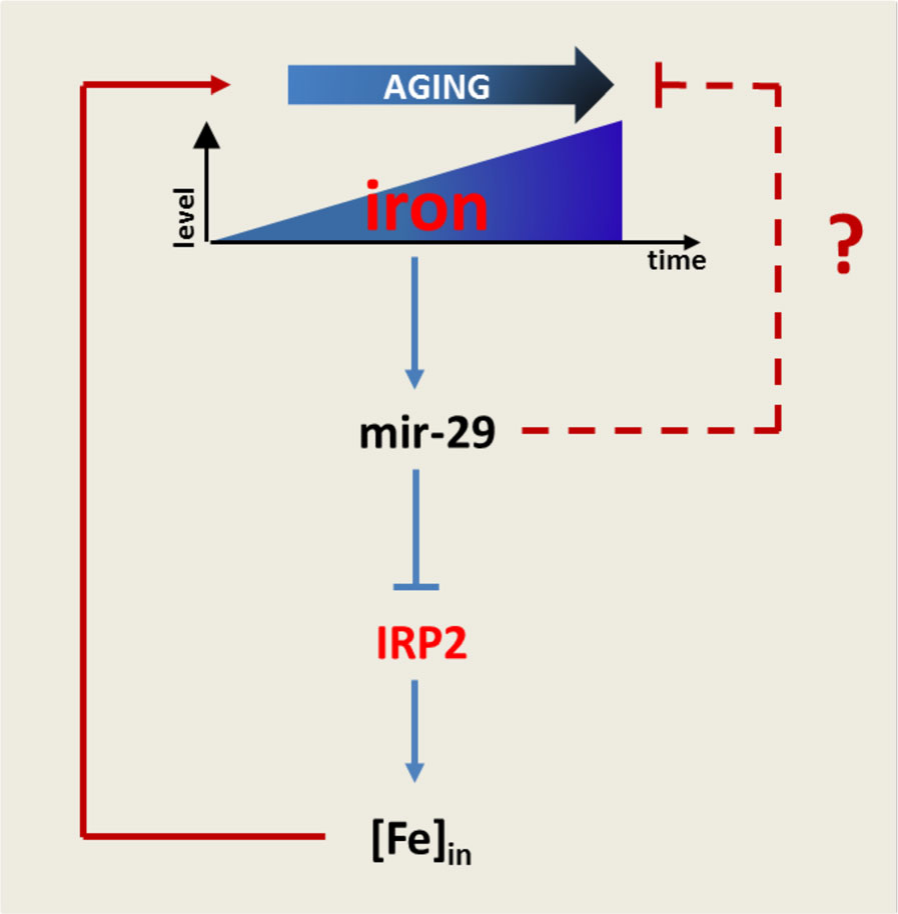
Schematic model of miR-29 action in brain aging. During aging an accumulation of iron in neurons occurs. This induces expression of miR-29 that in turn represses expression of IRP-2 thereby limiting iron uptake. This mechanism counteracts aging-related damages. MiR-29 may also counteract effects aging-related phenotypes by additional mechanisms, for example modulation of pro-apoptotic BCL-2 family members [21, 22]

Age-related damage accumulation is an inescapable condition that tends to change cellular homeostasis; on the other hand, cells tend to maintain their homeostasis inducing a progressive and adaptive response in order to counteract this inevitable process and preserve their physiological functions. Age-dependent up-regulation of miR-29 is part of this adaptive response and its deficiency leads to exacerbation of aging-induced damage (Fig. 7), partly due to impaired iron homeostasis.

## Methods

### Fish maintenance

#### N. furzeri

All experiments were performed on group-house *N. furzeri* of the MZM-04/10 strain. The fish used were raised in 35-L tanks at 25 °C and were fed two to three times a day with frozen *Chironomus* larvae or living nauplii of *Artemia salina*, depending on size. Eggs were collected by sieving the sand with a plastic net and kept in wet peat moss during developmental processes and diapauses. Embryos were hatched by flushing the peat with tap water at 16–18 °C. Embryos were scooped with a cut plastic pipette and transferred to a clean vessel. Fry were fed with newly hatched Artemia *nauplii* for the first 2 weeks and then weaned with finely chopped Chironomus larvae.

#### Danio rerio

All experiments were performed on the Ab strain. Fish were maintained at 28 °C under continuous flow in zebrafish facility with automatic control for a 14-hour light and 10-hour dark cycle (Zebtech system). To generate embryos for injection, male and female fish were placed the night before injection in a 1-L fish tank with the inner mesh and divider. Zebrafish embryos were obtained from natural spawning by removing the divider and light stimulation.

### Vector design and generation of transgenic lines

For the generation of all transgenesis vectors, we used multisite gateway technology. Design of vector *kif5aa:eGFP-sponge-29*: mir-29 sponge consisted of seven repetitions of mir-29 complementary sequences with a mismatch of four nucleotides immediately after the seed sequence. The sponge sequence was previously chemically synthetized by Eurofin (Milan) then cloned in the 3′ entry p3E-polyA using the restriction site BamHI. A 3,094 kb zebrafish kif5aa promoter region (Source: ZFIN; Acc:ZDB-GENE-070912-141) was amplified by PCR from genomic DNA (from –2969 to +125 bp including a small portion of the first exon, before the ATG), using primers Kif5aa-SalI f: (5′-gtcgacGTTGTCCAGCACGGATGTATAGGTA-3′) and Kif5aa-XmaI r:(5′-cccggg-ATAGCGGGGCAGAGGACGGCAG-3′) and cloned into gateway 5′-entry p5E-MCS. Then, the multisite gateway recombination reaction was performed as described in the Invitrogen Multi-Site Gateway Manual, an equimolar amount of entry vectors (20 fg of each; p5E-kif5aa, pEM-EGFPCAAX, and p3E-sponge-20-SV40pa) and destination vector (pDESTol2CG2) were combined with LR Clonase II Plus enzyme mix. For the generation of *actb2:eGFP-sponge-29* vector was used as 5′entry p5-bactin2 (from Tol2kit v1.2) together with the other plasmids following the protocol mentioned above. All these vectors also contain the eGFP reporter gene driven by the zebrafish cardiac myosin light chain (cmlc2) promoter. All vectors generated were extracted and purified using Qiagen plasmid midi kit (toxin free). Eggs were harvested and injected at zygote stage as described in the following protocols ([79] and [80] for *N. furzeri* and *D. rerio* microinjections, respectively). For microinjection borosilicate microcapillaries were used. Capillaries were pulled with a micropipette puller (P97, Sutter Instrument). The needles were filled with 2 μL of water solution containing 20–30 ng/μL of plasmid DNA, 20–30 ng/μL of Tol2 transposase mRNA, 0.4 M KCl, and 1% phenol red as visual control of successful injections. Embryos were injected with approximately 2 nL of mix solution and the drop volume was estimated under a microscope using a calibrated slide. Injections were performed under the Nikon C-PS stereoscope. F0 fishes were screened under fluorescence microscope at 3–4 days after hatching (*N. furzeri*) and at 24 hpf for zebrafish larvae. Three different founders were selected for line establishment.

### *Ireb2* 3′-UTR vectors assembly and mir-29 targeting validation

In order to experimentally validate *Ireb2* as direct mir-29 target in fishes, 671 and 633 bp from *Ireb2* 3′-UTR of *N. furzeri* and *D. rerio*, respectively, were amplified by PCR from cDNA of both species (*N. furzeri* reference: Nofu_GRZ_cDNA_3_0193494, *D. rerio* reference: ZFIN; Acc:ZDB-GENE-051205-1) using the primers nf*Ireb2*-3′UTR-BamH-F: (5′-ttcgggggatccCATGTTTGACTCTGAGAAGGAC-3′) and nf*Ireb2*-3′UTR-BamH-R: (5′-ttcgggggatccGTTCTGTGCCCAGTTTGCCC-3′) for *N. furzeri* and Dr*Ireb2*-3′UTR-BamH-F: (5′-gtcacgC-GGATCCTCACATGGACCTCTGAACACC-3′) and Dr*Ireb2*-3′UTR-BamH-R: (5′-gatcggc-GGATCCCACAGCGAAAGTATCACAGCC-3′) for *D. rerio* and cloned in the 3′ entry p3E-polyA. Then p5E-CMV/SP6, pME-EGFPCAAX, p3E-*Ireb2*-3′UTR-polyA, and pDESTol2CG2 were combined using multisite gateway (see above). Finally, the plasmid producing fluorescence standard control was assembled combining p5E-CMV/SP6, pME-RFP, p3E-polyA and pDESTol2CG2. We renamed these plasmids as N.f CMV/SP6: eGFP-*Ireb2*-pA, D.r CMV/SP6: eGFP-*Ireb2*-pA and CMV/SP6: RFP-pA. For all of these reporters, in vitro transcription was carried out as described below. About 2 nL of solution containing 100 pg of egfp-*Ireb2* sensor mRNA, 100 pg of RFP standard, 100 pg of Dre-mir-29a mimic or 100 pg of Dre-mir-29b mimic (Qiagen) was microinjected in the zygote stage embryos (for the control embryos the same mix was assembled without microRNA mimic). At 24 hpf, 30–40 embryos were collected from each experimental condition, dechorionated and dissociated according to Gallardo et al. [48]. Dissociated cells were diluted in PBS 1×, loaded in the flow cytometer machine (FacsCalibur, Becton-Dickinson) and the eGFP/RFP fluorescence ratio was analyzed and quantified. For mmu-*Ireb2* 3′-UTR validation we used dual luciferase reporter assay system (Promega). Mmu-*Ireb2* 3′utr (transcript ID: ENSMUST00000034843) containing the putative mir-29 binding site was cloned in pMIR-REPORT™ luciferase reporter system (Termo Fisher) using the restriction enzymes SpeI and MluI. Mmu-mir-29b1/a precursor was cloned in CMV/SP6: RFP-Pa vector, downstream of RFP using the restriction enzyme MluI. Transfection on Hek293 was performed using lipofectamine 2000 (Invitrogen). CMV:RFP-mir-29b/c precursor-polyA-tail was co-transfected with pMIR-report and Renilla control vector following the manufacturer’s instructions. In the control experiment the same vector was co-transfected without the mir-29a/b1 precursor sequence. At 24 hours post transfection both Renilla and Firefly luciferase activity were measured using the luminometer (GloMax 96 microplate Luminometer w/dual injectors). Mutant vectors were generated using the QuikChange II XL Site-Directed Mutagenesis kit (Stratagene) according to the manufacturer’s protocol.

### In vitro RNA and DIG-labeled probes synthesis

The Tol2 mRNA was transcribed from the pCS-TP plasmid whereas eGFP-*Ireb2*-3′utr and standard fluorescence control mRNAs were transcribed from plasmids: N.f CMV/SP6: eGFP-*Ireb2*-pA, D.r CMV/SP6: eGFP-*Ireb2*-pA and CMV/SP6: RFP-pA, respectively. All were previously linearized using NotI and purified with Wizard® SV Gel and PCR Clean-Up System (Promega). Then, 1 μg of each linearized plasmid was transcribed using the mMESSAGE mMACHINE SP6 kit (Ambion) according to the manufacturer’s protocol. For DIG-labeled probe synthesis, initially sequences were amplified by PCR from cDNA using a reverse primer carrying a T7 promoter sequence on its 5′ end; 200 ng of PCR product, previously purified with Wizard® SV Gel and PCR Clean-Up System (Promega), were directly transcribed using T7 enzyme (Fermentas) and digoxygenated RNTP mix (Roche) for 2 hours at 37 °C. The in vitro transcription mix was precipitated with 1/10 of volume of LiCl (5 M) and 2.5 volumes of isopropanol, than washed with 70% ethanol and finally resuspended in nuclease-free water and stored at –80 °C.

### Total RNA extraction and RT-qPCR

Dissected tissues were immediately put in 500 μL of Qiazol lysis reagent and manually homogenized with pounder. Total RNA was extracted using miRneasy mini kit (Qiagen) according to the manufacturer’s protocol; 200 ng of each RNA extraction was retrotranscribed for cDNA synthesis using miScript II RT kit (Qiagen). qPCR was performed using Rotorgene 6000 (Corbet). PCR mix solutions were prepared using SsoAdvanced™ Universal SYBR® Green Supermix (Biorad) and 4 ng of cDNA for each sample as template. The relative gene quantification was calculated using the ΔΔCt method, as reference genes were used *TBP*, *ACTB2*, and *GAPDH* for *N. furzeri*, *D. rerio*, and mouse, respectively.

### Histology and histochemistry

All the immunohistochemical procedures were performed on frozen tissue sections. Animals were killed with overdose of MS-222, brains were dissected and fixed in PFA 4%, washed in PBS 1× twice then equilibrated in sucrose 30% and embedded in Tissue-Tek OCT (Leica). Frozen tissues were cut with cryostat (Leica), 12- to 14-μm thick sections were immediately put on superfrost plus slides (Thermo Scientific) and dried in the oven at 55 °C for 1 hour. Sections were rehydrated in PBS 1× permeabilized with tritonX 0.3% and blocked in BSA 5%, goat serum 1%. Primary antibodies were all incubated overnight at 4 °C according to the following dilutions: Anti-HuC/D (1:50, Thermo Fisher Scientific Cat# A-21271 RRID:AB_221448), Anti-GFP (1:1000, Abcam Cat# ab32146 RRID:AB_732717), Anti-GFAP (1:400, Millipore Cat# MAB5324 RRID:AB_95211), Anti-S100 (1:400, Dako, zo311). Secondary antibodies coupled to Alexa Fluor dye (488, Molecular Probes Cat# A-11008 also A11008 RRID:AB_143165, 633, Molecular Probes Cat# A-21052 also A21052 RRID:AB_141459) were incubated for 2 hours at room temperature (RT) (1:500).

In situ hybridizations were performed according to [81] with minor modifications. Slides were incubated with proteinase K for 10 minutes at RT (1:80000; Fermentas, 20 mg/mL), post-fixed with PFA 4% for 20 minutes at RT, and then incubated with a digoxigenin (DIG)-labeled probes (60 °C, ON). Immediately before incubation probes were put in hybridization solution denaturated at 98 °C for 3 min. Sections were washed with SSC-2× twice at 60 °C for 15 min each, then with SSC-0.5× three times for 10 min each time at RT and incubated with anti-Dig AP Fab (Roche; 1/2000 4 °C ON) in blocking solution (Roche). The day after, slides were placed for 20 min RT in TMN solution (Tris-MgCl2-NaCl buffer) with the addition of levamisole 1 mM (Sigma) in order to inhibit endogenous alkaline phosphatase and transferred in Fast red solution (Roche tablets). The staining was constantly monitored under epiflourescence microscope and blocked by washing in PBS 1×. Images were acquired using epifluorescence microscope (Nikon, Eclipse600) or confocal microscope (Leica DMIRE2).

For lipofuscin detection, unstained sections were deparaffinized and mounted using a water-based medium within DAPI (Invitrogen). An important property of lipofuscin is its broad autofluorescence, which was acquired using a Zeiss apotome(2) at an excitation wavelength of 488 and 550 nm as well as under UV excitation following DAPI staining. Images were analyzed using ImageJ, thresholds were determined by operator and applied to images to discriminate lipofuscin granules from background signal. The area occupied by granules was expressed as a percentage of total image area analyzed.

### Iron staining (Perls staining)

Iron staining was performed according to [82] with minor modifications. Briefly, brains were dissected and fixed in PFA 4% and embedded in paraffin. After this, 5- to 7-μm thick sections were deparaffinized and immersed in 4% ferrocyanide and 2% HCl for 1 hour at 37 °C, then immersed in methanol containing 0.5% H_2_O_2_ and 0.01 NaN_3_ for 30 min at RT, and finally immersed in 0.1 M phosphate buffer containing DAB 0.05% and 0.005% H_2_O_2_ for 30 min. Sections were counterstained with hematoxylin, dehydrated and mounted.

### Non-heme iron quantification

Iron quantification was performed according to the protocol published by [60]. Extracted tissues were placed in an empty 1.5 mL tube, previously weighed, and were weighed again in order to precisely determine wet tissue weight. Homogenates were prepared in high-purity water. Briefly, 100 μL of homogenate tissues were combined in a new 1.5 mL tube with an equal volume of protein precipitation solution (1 N HCl, 10% trichloroacetic acid) and placed in thermoblock at 95 °C for 1 hour. Tubes were cooled to RT for 10 min and were then centrifuged for 20 min at 4 °C; 100 μL of the supernatant was collected from each tube sample and combined with an equal volume of chromogenic solution (Ferrozine 0.5 mM, ammonium acetate 1.5 M and Tioglicolic acid 0.1%). After 30 min, absorbance was measured at 562 nm using a spectrophotometer. Standard curves were prepared using 0, 0.5, 1, 2, 4, 8, 10, and 20 μg/mL of iron standard solution (Sigma).

### Iron and drug delivery

Fish were previously anesthetized with Trichaine methanesulfonate and weighed. A single dose respectively of 350 μg/g body weight iron dextran (Sigma, 100 mg/mL), 30 μg/g body weight deferoxamine (DFO, Novartis), and 50 μ/g body weight 4-OH-Tempol (Sigma) was injected intraperitonealy. Injections were performed under a stereomicroscope (Leica) using Amilton 10-μL syringes, control animals were sham-injected with saline solution. At 4, 8, 12, 24, 48, and 72 hours after injection brains were removed from anesthetized animals and put in a previously weighed tube for iron measurement or immediately frozen in liquid nitrogen for the following RNA extraction.

### Western blot

Samples were lysed in RIPA-buffer (50 mM Tris-HCl, pH 7.5, 150 mM NaCl, 1% Triton X-100, 0.1% SDS, 0.5% deoxycolic acid) containing protease inhibitor (Complete Protease Inhibitor Cocktail Tablets, Roche Diagnostics) and centrifuged at 12,300 rpm for 10 min at 4 °C and supernatants were collected. Total protein concentration was determined by BCA protein assay (Pierce). Aliquots of homogenate with equal protein concentrations were separated in 10% acrylamide gel and transferred to nitrocellulose membranes by mini trans-blot (Bio-Rad). The membranes were blocked with milk (5% w/v) and probed with appropriate primary and secondary IgG-HRP conjugated antibodies (Millipore). Enhanced chemiluminescence detection system (GE Healthcare) was used for developing on autoradiography-films (GE Healthcare). Densitometric quantification was performed using ImageJ and normalized to the relative amount of α-Tubulin and expressed as n-fold of control samples. In this study the following antibodies were used: Anti-IRP2 (1:1000, Abcam, ab181153), Anti-TFRC (1:500, Abcam Cat# ab84036 RRID:AB_10673794), Anti-FBXL5 (1:500, Abcam Cat# ab68069 RRID:AB_1140312), α-TUB (1:20000, Sigma-Aldrich Cat# T5168 RRID:AB_477579); secondary HRP antibodies: goat-anti rabbit (1:2000, Santa Cruz Biotechnology Cat# sc-2004 RRID:AB_631746), goat-anti mouse (1:2000, Santa Cruz Biotechnology Cat# sc-2005 RRID:AB_631736).

### Cell culture and iron exposure

Following a previously published protocol [63], murine ES cell line E14Tg2A was differentiated into cortical neurons. In brief, mouse embryonic stem cells (mESC) were cultured in a chemically defined medium on laminin-coated culture dishes for 20 days. mESC-derived neurons were then treated with iron dextran (50, 100, and 200 μg/mL) and Iron(II) sulfate (25, 50 and 100 μM) in Neurobasal Medium (Thermo Fisher Scientific) supplemented with B27 (Thermo Fisher Scientific) for 72 h, medium was changed daily. Subsequently, two wells of a six-well plate were pooled for each thesis; RNA extraction and PCR analysis were carried out from three biological replicates.

### RNA-sequencing and analysis

RNA was extracted using Quaziol (Qiagen). Library preparation using Illumina’s TruSeq RNA sample prep kit v2 and sequenced on Illumina HiSeq2500, 50 bp single-read mode in multiplexing obtaining approximately 50–40 mio reads per sample. Read mapping was performed using Tophat 2.0.6 [83] and featureCounts v1.4.3-p1 [84] using the *N. furzeri* genome [47] or Zv9.73 as references. Differential gene expression analysis was performed using R software, for DEGs identification was used the statistical test of edgeR package [85]. For multiple testing correction a FDR of < 0.05 was chosen. KEGG pathway analysis was performed using the software Web-based gene set analysis tool kit (WebGestalt) [86] using an FDR < 0.05.

### Annotation of small RNAs

The processing and annotation of small RNA-Seq raw data was performed using an R 3.0.2 and ShortRead Bioconductor package [87].

First, raw data were preprocessed with the following parameters:

- Quality filtering: eliminated all the reads containing an “N”;
- Adapter trimming: used function trimLRPatterns(), allowing up to two mismatches and using as adapter sequence “TGGAATTCTCGGGTGCCAAGGAACTCCAGTCAC”;
- Size filtering: removed all the reads with length < 18 and > 33 nucleotides.

Second, reads were aligned, resulting in a direct annotation and quantification. The alignment was divided in two steps to allow the recognition and the annotation of the reads exceeding reference length. In fact, the algorithm of Bowtie 1.0.0 does not allow aligning longer reads to shorter references. Specifically,

- Alignment against the reference (miRBase 20; [88]) with up to two mismatches. In this step the reference used was the mature sequence of microRNAs for the analyzed organism (except for *N. furzeri*, for which *D. rerio* reference was used). Each read was aligned using these criteria with Bowtie 1.0.0 (settings:-q --best –norc);
- The remaining reads, which could not align in the previous step, were used as reference for a second alignment step. In this case, the annotated mature microRNAs were aligned against the reads;
- The information obtained in the two alignment phases was conveyed in one single table, containing a list of all the retrieved sequences and their relative counts.

### Enrichment analysis of miR-29 binding sites

Target predictions were obtained using MiRanda (version: august 2010) with default parameters [89].

Enrichment analysis was performed to test the overrepresentation of specific microRNA targets in the identified transcript clusters of genes with different expression profiles with age [46]. For this purpose, three different tests were used: Binomial test, Fisher’s test and Hypergeometric test. Only microRNAs having a Hochberg’s corrected *P* value < 0.05 in the three tests were considered as having their targets overrepresented in one cluster.

## Additional files

Additional file 1: Prediction of miR-29 binding site in *D. rerio Elna1* and *col11a1a* mRNAs. Representative 3′-UTR of *Elna1* (ENSDARG00000069994.1) and *Col11a1a* (ENSDARG00000026165.1), respectively, with indication of the predicted binding sites (from TargetScanFish 6.2). Black circles shows miR-29 binding site and blue table shows the score of all predicted microRNA binding sites, miR-29 has the highest score in both the 3′-UTR genes according to TargetScanFish 6.2. (PDF 531 kb)
Additional file 2: Neuronal-specific kif5a promoter expression. Representative double-labelling immunohistochemistry for eGFP (green) and neuronal marker HuC/D (pink) in optic tectum of 20-week-old kif5a:sponge-29 fish. Periventricular gray zone (PGZ), Tectum opticum (TeO). Scale bar: 100 μm. (PDF 1014 kb)
Additional file 3: RNA-seq data analysis (DEG list). (XLSX 156 kb)
Additional file 4: Complete list of KEG pathway analysis. (PDF 839 kb)
Additional file 5: Complete list of genes coherently or incoherently regulated by aging and sponge-29. (XLSX 43 kb)
Additional file 6: Mir-29 predicted target DEGs identification in *N. furzeri* kif5a: sponge-29. **A** Intersection of *D. rerio* miR-29 predicted target genes (from TargetScanFish 6.2, score ≤ –0.30) with *N. furzeri* sponge-29 DEGs. A total of 38 predicted target genes were found deregulated in kif5a:sponge-29 fish, 28/38 upregulated (**B**) and 10/38 downregulated (**C**), respectively. Gray, red and green box represent a conserved binding site in the 3′-UTR of *N. furzeri*, *M. musculus*, and *H. sapiens*, respectively. Yellow colored genes lack a conserved binding site in *N. furzeri*. Light blue box indicate a physical interaction between target gene e and miR-29 observed by cross-linking immunoprecipitation (CLIP-seq). 1-3-4 numbers are relative to gene dataset type in Fig. 3e. (PDF 78 kb)
Additional file 7: Mir-29 target *D. rerio and M. musculus Ireb2* 3′-UTR. **A** Expression of GFP assessed by cytofluorometric analysis. Fusion with *Ireb2* 3′-UTR of *D. rerio*. The gray column indicates the baseline fluorescence intensity of the construct without miR-29 mimic. The middle blue column indicates the fluorescence with miR-29 mimic and the red column the fluorescence of a construct (Δ) where the putative binding site for miR-29 in the *Ireb2* 3′-UTR was mutated to destroy complementarity. Statistical significance of fluorescence difference between baseline and co-injection with miR-29 mimics was evaluated by Student’s t-test (** *P* < 0.01). **B** 293 T cells co-transfected with renilla and firefly luciferase construct containing the wild-type or the mutant target sites of mmu-*Ireb2* 3′-UTR along with the miRNA expression plasmid (pcs2+: CMV:RFP-miR-29b/c precursor-polyA-tail) or the empty vector (pcs2+: CMV:RFP-polyA-tail). Histograms show normalized (to renilla) sensor luciferase activity of empty vector transfected cells (gray column) respect to transfected cells with miRNA expressing vector (blue column) or to mutated sensor with miRNA expressing vector. Bars represent mean ± standard deviation (Student’s t-test, ***P* < 0.01) derived from two independent experiment performed in triplicates for each condition. (PDF 41 kb)
Additional file 8: Age dependent regulation of IRP2 in wild-type and *tg(kif5aa:eGFP-sponge-29).* **A**, **B** IRP2 western blot analysis at different age point of wild-type (A) and *tg(kif5aa:eGFP-sponge-29)* brains. Three biological replicates were used for age point. α-tubulin was used as loading control. (PDF 163 kb)
Additional file 9: Mir-29 upregulation coincides with *Ireb2* and *Tfrc* downregulation in mouse brain. **A** Quantification of pri-miR-29 cluster (pri-miR-29b-a; pri-miR-29b-c) in postnatal day 0 and 60 mouse brain. Both primary transcripts dramatically increase during postnatal development (*P* < 0.001 and *P* < 0.001, respectively). **B** Relative expression of iron management genes *Ireb2* (*P* < 0.01) and *Tfrc* (*P* < 0.05), in postnatal day 0 and 60 mouse brain. Both for A and B statistical significance was calculated by the Mann–Whitney U-test, *n* = 6 (P0 brain) and *n* = 7 (P60 brain) biological replicates. (PDF 39 kb)
Additional file 10: Mir-29 loss of function induces gliosis. Representative images of immunoreactivity for GFAP and S100β markers in the optic tectum of 12-week-old kif5a:eGFP-sponge-29 and wild-type fish brains and relative quantification expressed as GFAP/S100β intensity per area of section. The analysis was performed in n = 5 wild-type and n = 5 kif5a:sponge-29 fish brains (**P* < 0.05, Mann–Whitney U-test). Scale bar 100 μm. (PDF 13306 kb)
Additional file 11: Supporting Data: Raw quantitative PCR data relative to Figs. 1b and 6c–e, g, h. (XLSX 50 kb)
