# Supplementary material for: MicroRNA miR-29 controls a compensatory response to limit neuronal iron accumulation during adult life and aging
Source: BMC Biol. 2017 Feb 13;15:9. doi: 10.1186/s12915-017-0354-x (PMC5304403; doi:10.1186/s12915-017-0354-x)
Supplement: Additional file 1: — Prediction of miR-29 binding site in D. rerio Elna1 and col11a1a mRNAs. Representative 3′-UTR of Elna1 (ENSDARG00000069994.1) and Col11a1a (ENSDARG00000026165.1), respectively, with indication of the predicted binding sites (from TargetScanFish 6.2). Black circles shows miR-29 binding site and blue table shows the score of all predicted microRNA binding sites, miR-29 has the highest score in both the 3′-UTR genes according to TargetScanFish 6.2. (PDF 531 kb) [file 12915_2017_354_MOESM1_ESM.pdf]

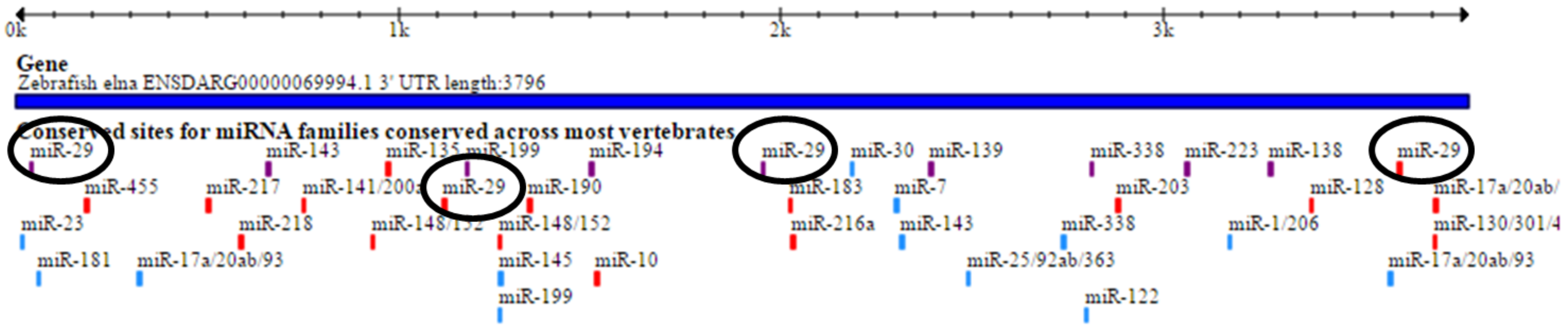

miRNA	Sites				Total Context score
	Total	8mer	7mer-m8	7mer-1A	
miR-29	4	2	2	0	-1.01
miR-138	1	1	0	0	-0.26
miR-143	2	1	0	1	-0.23
miR-17a/20ab/93	3	0	1	2	-0.21
miR-130/301/454	1	0	1	0	-0.21
miR-455	1	0	1	0	-0.20
miR-148/152	2	0	2	0	-0.17
miR-223	1	1	0	0	-0.13
miR-128	1	0	1	0	-0.13
miR-217	1	0	1	0	-0.13
miR-141/200a	1	0	1	0	-0.13
miR-139	1	1	0	0	-0.11
miR-135	1	0	1	0	-0.10
miR-199	2	1	0	1	-0.10

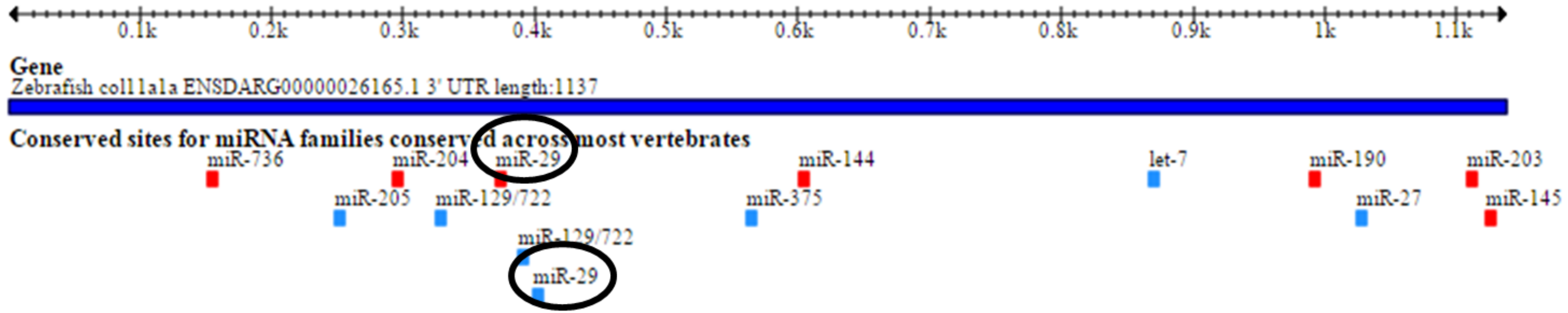

miRNA	Sites				Total Context score
	Total	8mer	7mer-m8	7mer-1A	
miR-29	2	0	1	1	-0.42
miR-145	1	0	1	0	-0.27
miR-190	1	0	1	0	-0.21
miR-203	1	0	1	0	-0.18
let-7	1	0	0	1	-0.15
miR-736	1	0	1	0	-0.13
miR-27	1	0	0	1	-0.10
miR-144	1	0	1	0	-0.10
