# Supplementary figures and images for: MicroRNA miR-29 controls a compensatory response to limit neuronal iron accumulation during adult life and aging
Source: BMC Biol. 2017 Feb 13;15:9. doi: 10.1186/s12915-017-0354-x (PMC5304403; doi:10.1186/s12915-017-0354-x)

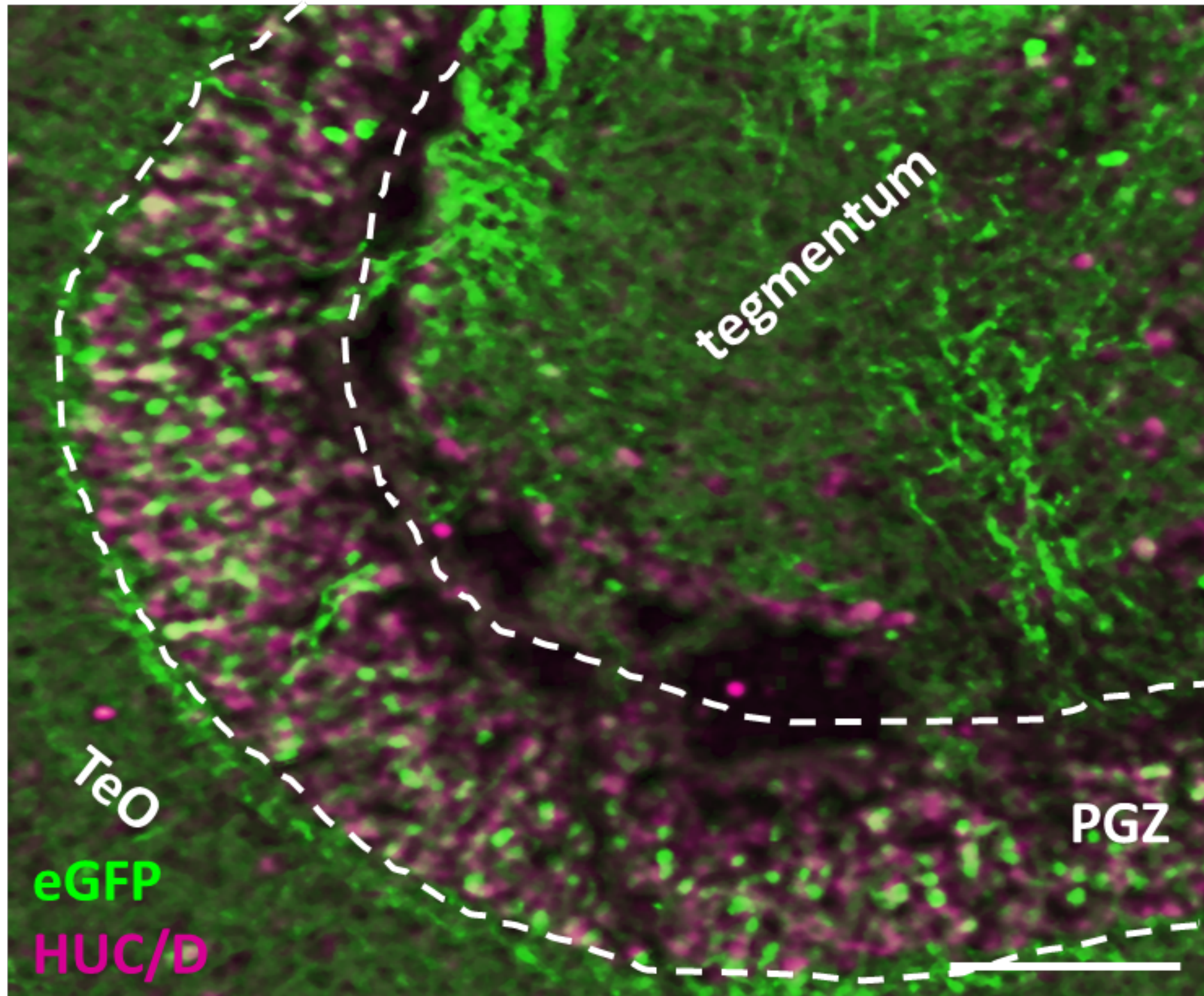

Supplement: Additional file 2: — Neuronal-specific kif5a promoter expression. Representative double-labelling immunohistochemistry for eGFP (green) and neuronal marker HuC/D (pink) in optic tectum of 20-week-old kif5a:sponge-29 fish. Periventricular gray zone (PGZ), Tectum opticum (TeO). Scale bar: 100 μm. (PDF 1014 kb) [file 12915_2017_354_MOESM2_ESM.pdf]

A

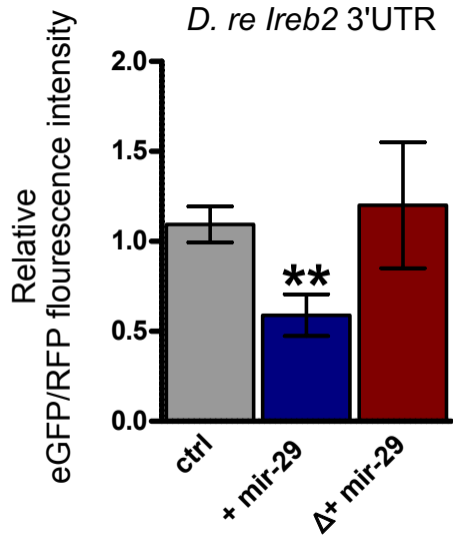

B

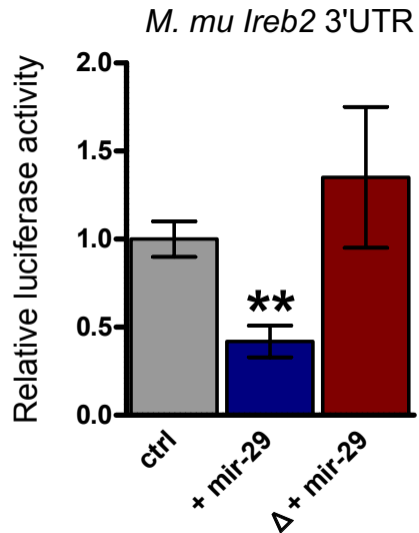

Supplement: Additional file 7: — Mir-29 target D. rerio and M. musculus Ireb2 3′-UTR. A Expression of GFP assessed by cytofluorometric analysis. Fusion with Ireb2 3′-UTR of D. rerio. The gray column indicates the baseline fluorescence intensity of the construct without miR-29 mimic. The middle blue column indicates the fluorescence with miR-29 mimic and the red column the fluorescence of a construct (Δ) where the putative binding site for miR-29 in the Ireb2 3′-UTR was mutated to destroy complementarity. Statistical significance of fluorescence difference between baseline and co-injection with miR-29 mimics was evaluated by Student’s t-test (** P < 0.01). B 293 T cells co-transfected with renilla and firefly luciferase construct containing the wild-type or the mutant target sites of mmu-Ireb2 3′-UTR along with the miRNA expression plasmid (pcs2+: CMV:RFP-miR-29b/c precursor-polyA-tail) or the empty vector (pcs2+: CMV:RFP-polyA-tail). Histograms show normalized (to renilla) sensor luciferase activity of empty vector transfected cells (gray column) respect to transfected cells with miRNA expressing vector (blue column) or to mutated sensor with miRNA expressing vector. Bars represent mean ± standard deviation (Student’s t-test, **P < 0.01) derived from two independent experiment performed in triplicates for each condition. (PDF 41 kb) [file 12915_2017_354_MOESM7_ESM.pdf]

A

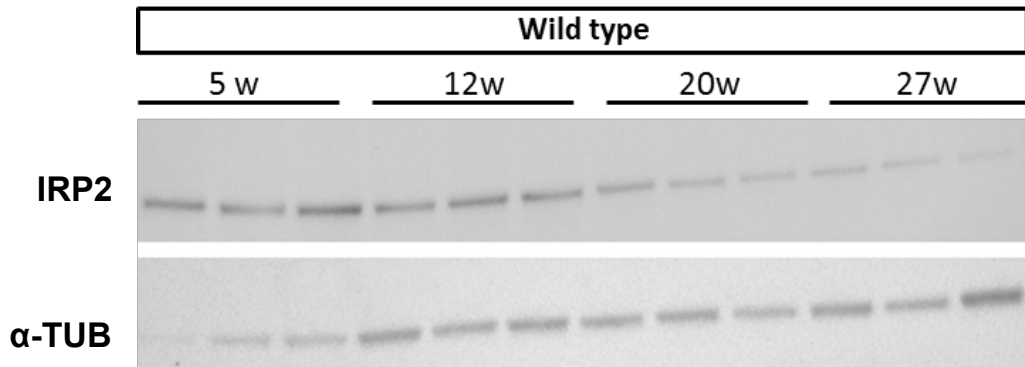

B

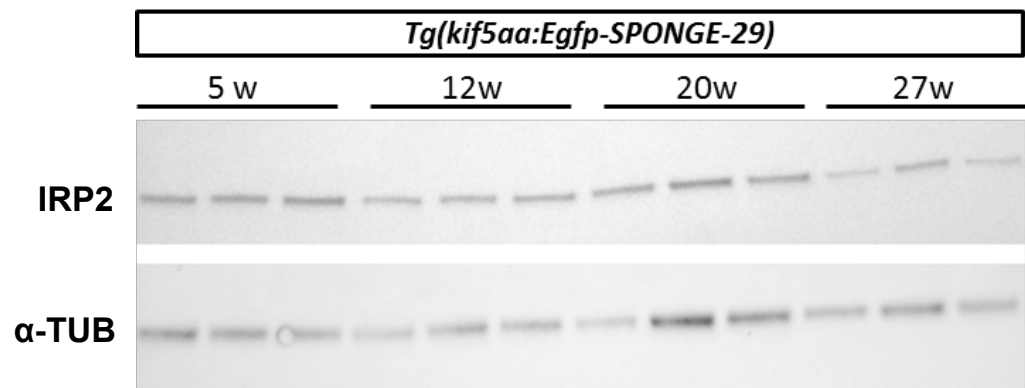

Supplement: Additional file 8: — Age dependent regulation of IRP2 in wild-type and tg(kif5aa:eGFP-sponge-29). A, B IRP2 western blot analysis at different age point of wild-type (A) and tg(kif5aa:eGFP-sponge-29) brains. Three biological replicates were used for age point. α-tubulin was used as loading control. (PDF 163 kb) [file 12915_2017_354_MOESM8_ESM.pdf]

A

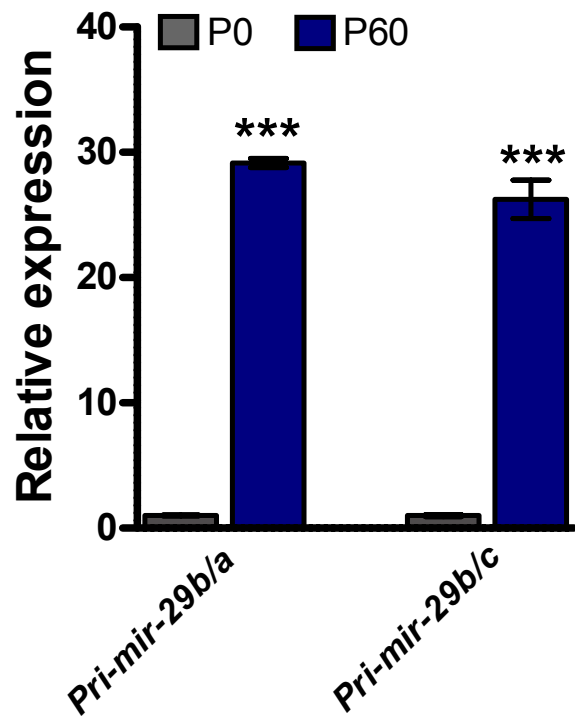

B

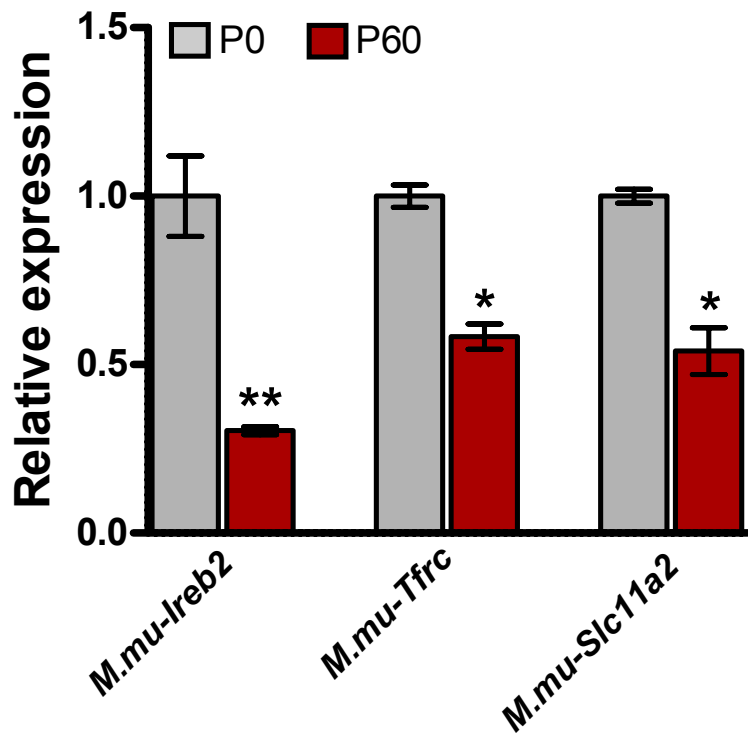

Supplement: Additional file 9: — Mir-29 upregulation coincides with Ireb2 and Tfrc downregulation in mouse brain. A Quantification of pri-miR-29 cluster (pri-miR-29b-a; pri-miR-29b-c) in postnatal day 0 and 60 mouse brain. Both primary transcripts dramatically increase during postnatal development (P < 0.001 and P < 0.001, respectively). B Relative expression of iron management genes Ireb2 (P < 0.01) and Tfrc (P < 0.05), in postnatal day 0 and 60 mouse brain. Both for A and B statistical significance was calculated by the Mann–Whitney U-test, n = 6 (P0 brain) and n = 7 (P60 brain) biological replicates. (PDF 39 kb) [file 12915_2017_354_MOESM9_ESM.pdf]

A

Wild type  
*tg(kif5aa:eGFP-sponge-29)*

DAPI

S100b

GFAP

MERGE

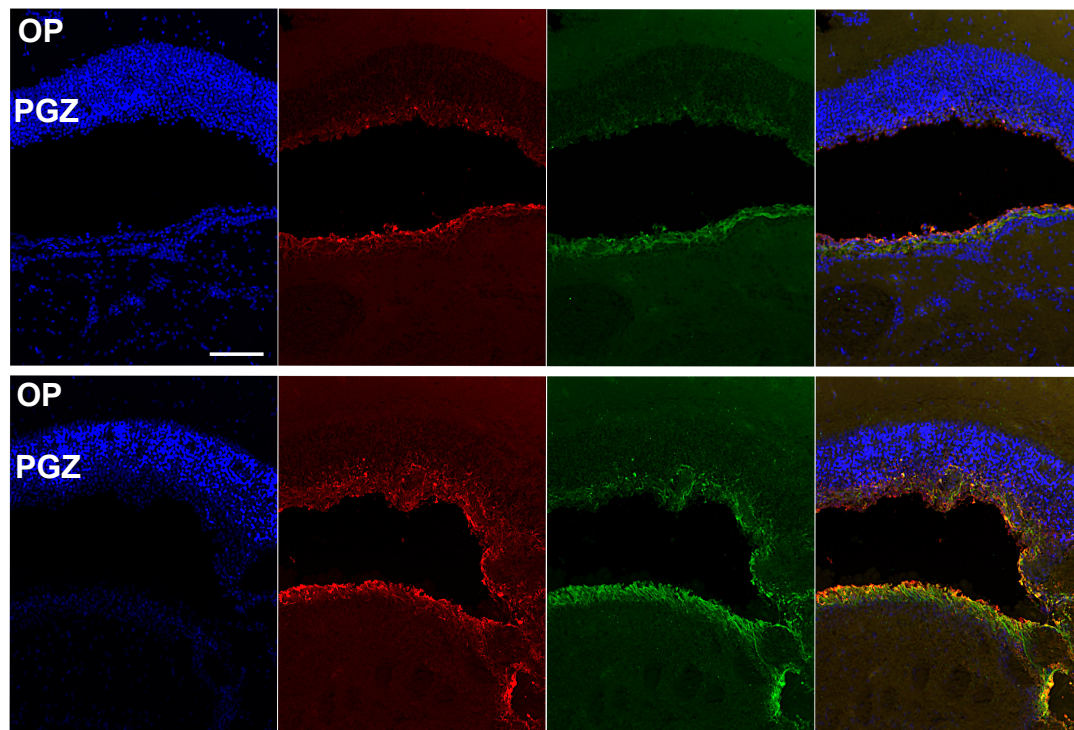

B

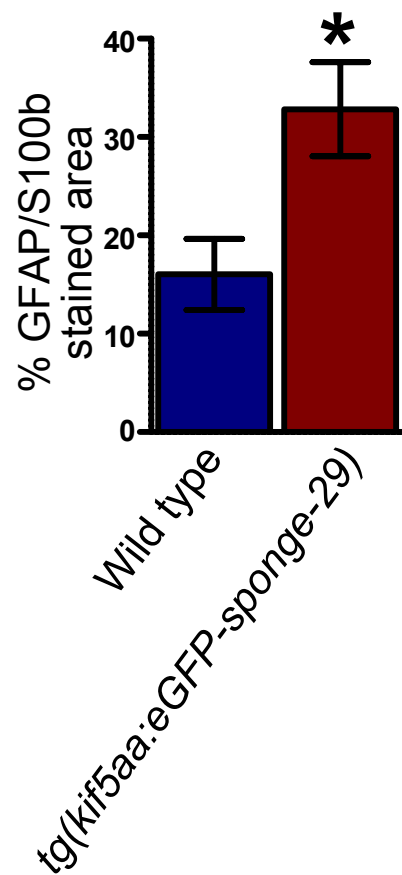

Supplement: Additional file 10: — Mir-29 loss of function induces gliosis. Representative images of immunoreactivity for GFAP and S100β markers in the optic tectum of 12-week-old kif5a:eGFP-sponge-29 and wild-type fish brains and relative quantification expressed as GFAP/S100β intensity per area of section. The analysis was performed in n = 5 wild-type and n = 5 kif5a:sponge-29 fish brains (*P < 0.05, Mann–Whitney U-test). Scale bar 100 μm. (PDF 13306 kb) [file 12915_2017_354_MOESM10_ESM.pdf]
