# Supplementary material for: MicroRNA miR-29 controls a compensatory response to limit neuronal iron accumulation during adult life and aging
Source: BMC Biol. 2017 Feb 13;15:9. doi: 10.1186/s12915-017-0354-x (PMC5304403; doi:10.1186/s12915-017-0354-x)
Supplement: Additional file 4: — Complete list of KEG pathway analysis. (PDF 839 kb) [file 12915_2017_354_MOESM4_ESM.pdf]

OXIDATIVE PHOSPHORYLATION

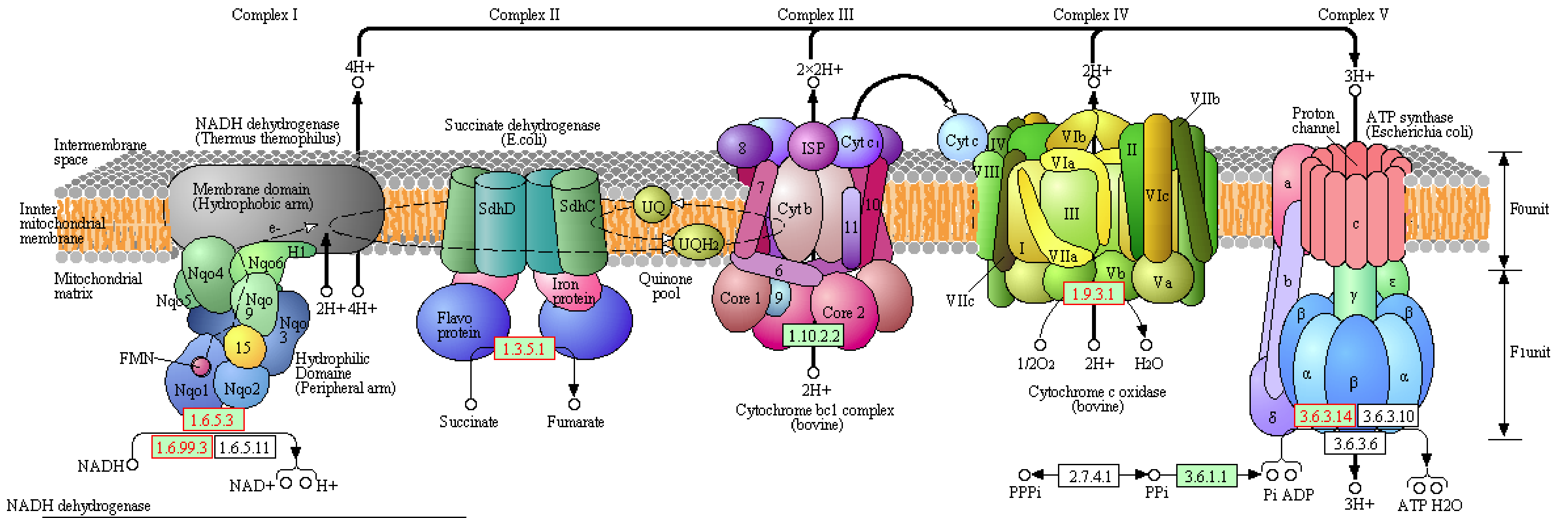

NADH dehydrogenase

E	ND1	ND2	ND3	ND4	ND4L	ND5	ND6									
E	Ndufs1	Ndufs2	Ndufs3	Ndufs4	Ndufs5	Ndufs6	Ndufs7	Ndufs8	Ndufv1	Ndufv2	Ndufv3					
B/A	NuoA	NuoB	NuoC	NuoD	NuoE	NuoF	NuoG	NuoH	NuoI	NuoJ	NuoK	NuoL	NuoM	NuoN		
B/A	NdhC	NdhK	NdhJ	NdhH	NdhA	NdhI	NdhG	NdhE	NdhF	NdhD	NdhB	NdhL	NdhM	NdhN	HoxE	HoxF	HoxU
E	Ndufa1	Ndufa2	Ndufa3	Ndufa4	Ndufa5	Ndufa6	Ndufa7	Ndufa8	Ndufa9	Ndufa10	Ndufab1	Ndufa11	Ndufa12	Ndufa13		
E	Ndufb1	Ndufb2	Ndufb3	Ndufb4	Ndufb5	Ndufb6	Ndufb7	Ndufb8	Ndufb9	Ndufb10	Ndufb11	Ndufc1	Ndufc2			

Succinate dehydrogenase / Fumarate reductase

E	SDHC	SDHD	SDHA	SDHB
B/A	SdhC	SdhD	SdhA	SdhB
		FrdA	FrdB	FrdC	FrdD

Cytochrome c reductase

E/B/A	ISP	Cyt b	Cyt 1				
E	COR1	QCR2	QCR6	QCR7	QCR8	QCR9	QCR10

Cytochrome c oxidase

E	COX10	COX3	COX1	COX2	COX4	COX5A	COX5B	COX6A	COX6B	COX6C	COX7A	COX7B	COX7C	COX8		COX17
E/B/A															COX11	COX15
B/A	CyoE	CyoD	CyoC	CyoB	CyoA											
		CoxD	CoxC	CoxA	CoxB											
		QoxD	QoxC	QoxB	QoxA											

Cytochrome c oxidase , cbb3-type

B	I	II	IV	III

Cytochrome bd complex

B/A	CydA	CydB

F-type ATPase (Bacteria)

alpha	beta	gamma	delta	epsilon
a	b	c		

F-type ATPase (Eukaryotes)

alpha	beta	gamma	delta	epsilon	
OSCP	a	b	c	d	e
f	g	f6/h	j	k	8

V/A-type ATPase (Bacteria, Archaeas)

A	B	C	D	E	F	G/H
I	K					

V-type ATPase (Eukaryotes)

A	B	C	D	E	F	G	H
a	c	d	e	S1			

00190 6/16/15

(c) Kanehisa Laboratories

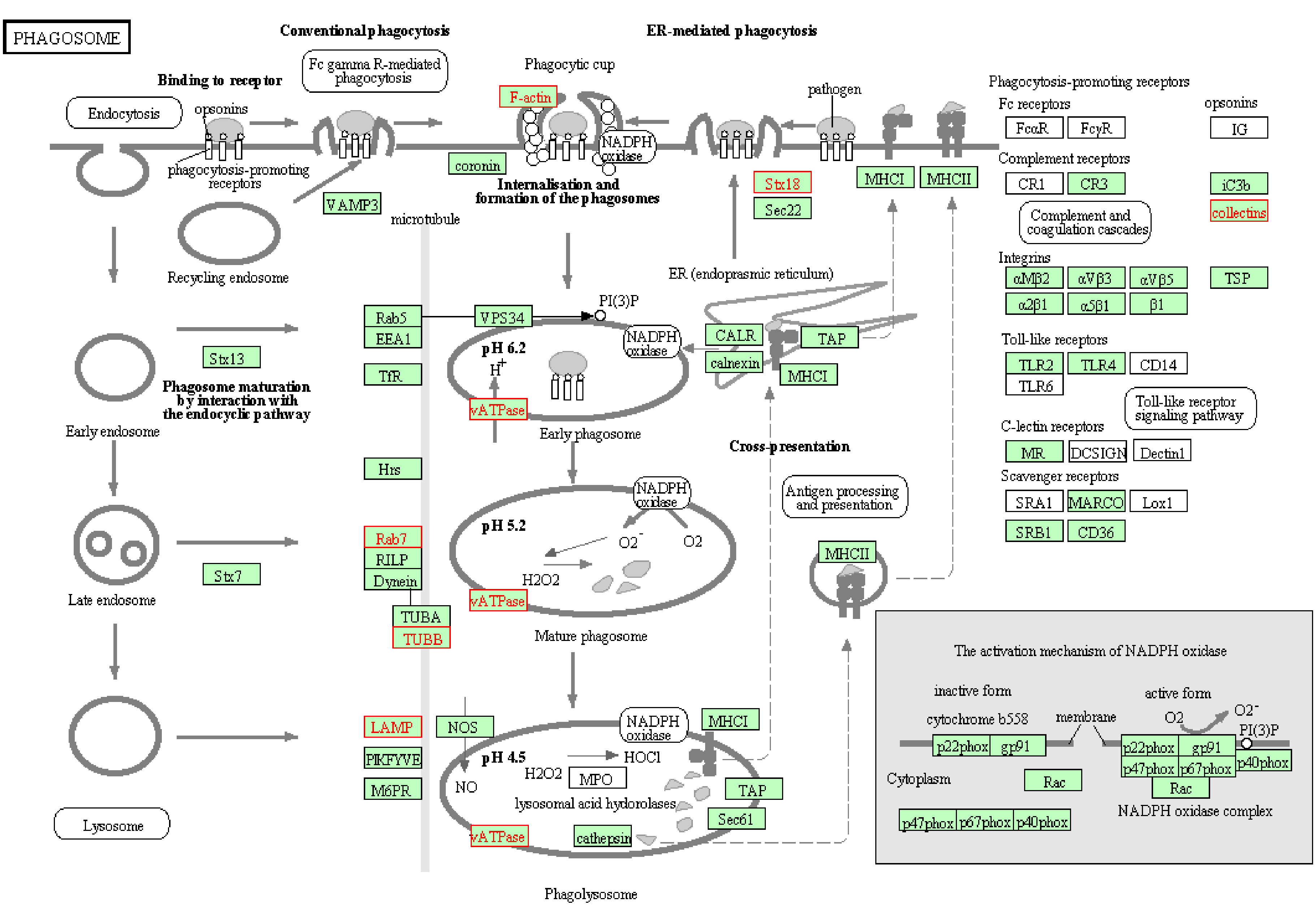

04145 5/14/14
(c) Kanehisa Laboratories

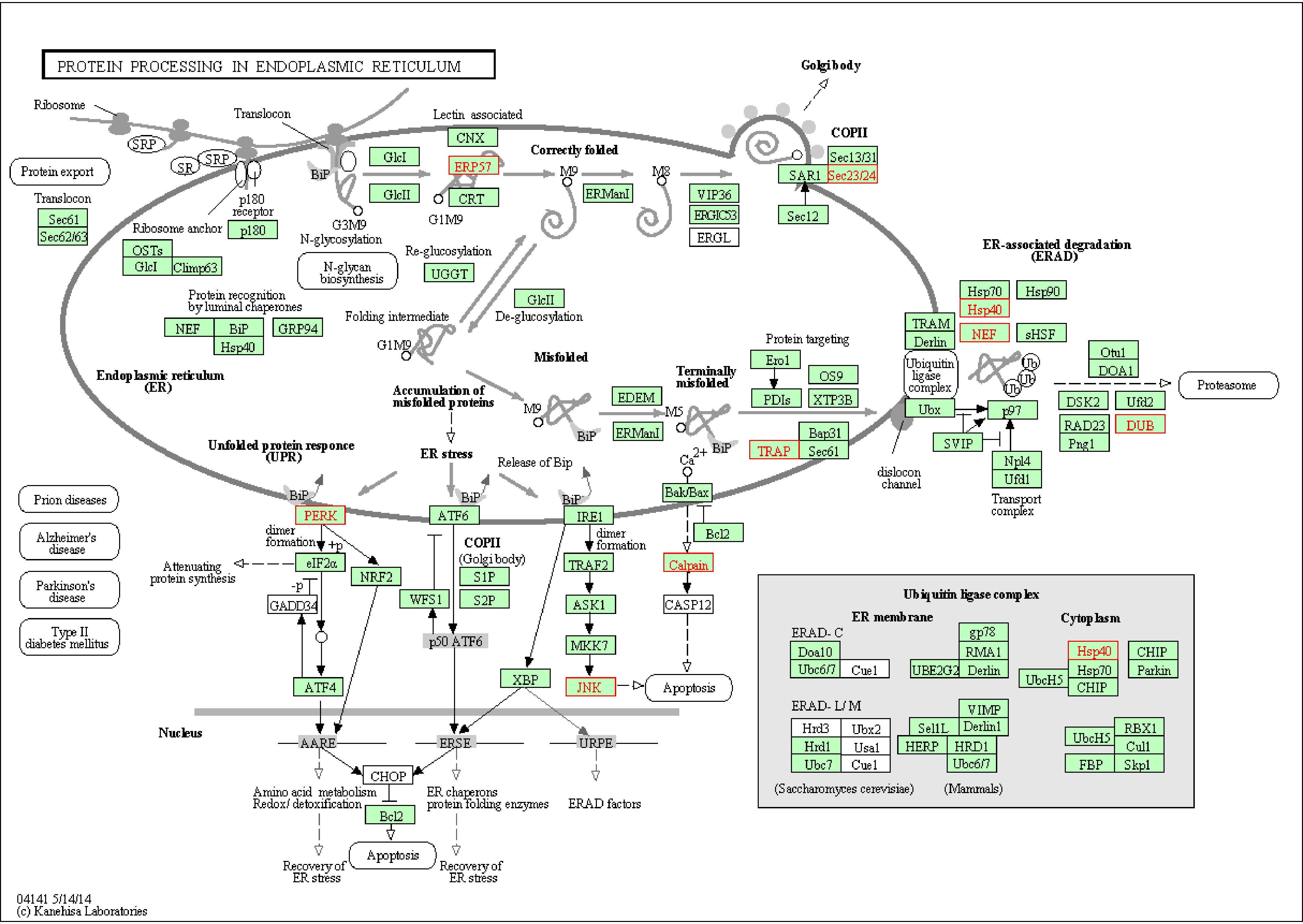
PROTEIN PROCESSING IN ENDOPLASMIC RETICULUM
Ribosome
SRP
SR
SRP
Translocon
Protein export
Translocon
Sec61
Sec62/63
p180 receptor
Ribosome anchor
p180
OSTs
GlcI
Climp63
BiP
G3M9
N-glycosylation
N-glycan biosynthesis
Lectin associated
CNX
GlcI
ERP57
GlcII
CRT
G1M9
Re-glucosylation
UGGT
Correctly folded
M9
ERManI
M8
VIP36
ERGIC53
ERGL
Golgi body
COPII
Sec13/31
SAR1
Sec23/24
Sec12
Protein recognition by luminal chaperones
NEF
BiP
GRP94
Hsp40
Folding intermediate
GlcII
De-glucosylation
G1M9
Endoplasmic reticulum (ER)
Misfolded
Accumulation of misfolded proteins
M9
EDEM
M5
ERManI
BiP
Terminally misfolded
Protein targeting
Ero1
PDIs
OS9
XTP3B
Bap31
TRAP
Sec61
BiP
Ca2+
ER-associated degradation (ERAD)
Hsp70
Hsp90
Hsp40
TRAM
Derlin
NEF
sHSF
Otu1
DOA1
Ubiquitin ligase complex
Ub
Ub
Ub
Proteasome
Ubx
p97
SVIP
DSK2
Ufd2
RAD23
DUB
Png1
Npl4
Ufd1
Transport complex
dislocon channel
Unfolded protein responce (UPR)
ER stress
Release of Bip
BiP
BiP
BiP
PERK
ATF6
IRE1
Bak/Bax
Bcl2
Prion diseases
Alzheimer's disease
Parkinson's disease
Type II diabetes mellitus
dimer formation
+p
eIF2α
NRF2
Attenuating protein synthesis
-p
GADD34
ATF4
COPII (Golgi body)
S1P
S2P
WFS1
p50 ATF6
dimer formation
TRAF2
ASK1
MKK7
JNK
XBP
Calpain
CASP12
Apoptosis
Nucleus
AARE
ERSE
URPE
CHOP
Amino acid metabolism
Redox/ detoxification
Bcl2
ER chaperons
protein folding enzymes
ERAD factors
Apoptosis
Recovery of ER stress
Recovery of ER stress
Ubiquitin ligase complex
ER membrane
Cytoplasm
ERAD- C
Doa10
Ubc6/7
Cue1
gp78
RMA1
UBE2G2
Derlin
Hsp40
Hsp70
CHIP
UbcH5
CHIP
Parkin
ERAD- L/ M
Hrd3
Ubx2
Hrd1
Usa1
Ubc7
Cue1
VIMP
Sel1L
Derlin1
HERP
HRD1
Ubc6/7
UbcH5
RBX1
Cul1
FBP
Skp1
(Saccharomyces cerevisiae)
(Mammals)
04141 5/14/14
(c) Kanehisa Laboratories

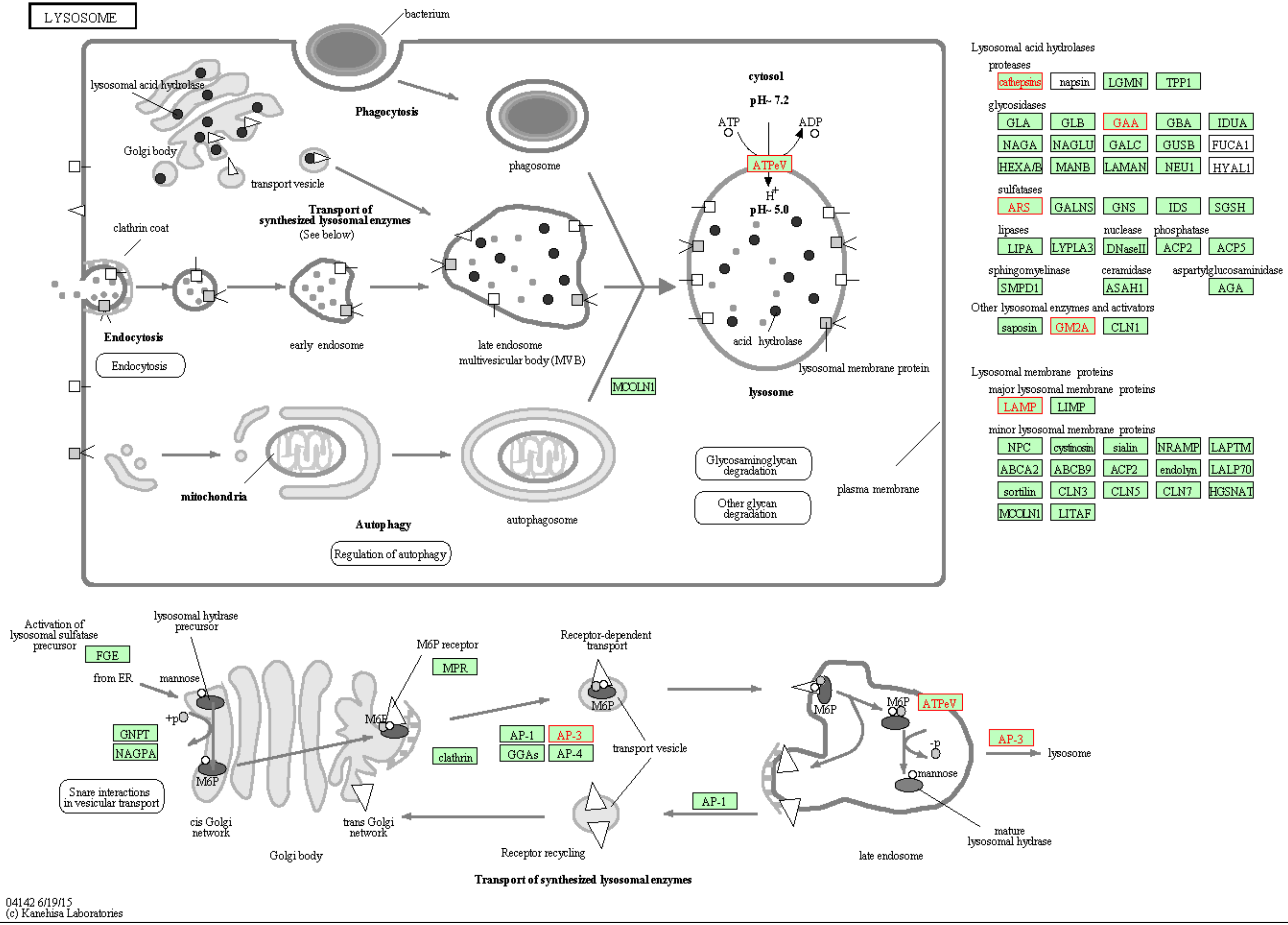
LYSOSOME
bacterium
lysosomal acid hydrolase
Golgi body
Phagocytosis
transport vesicle
phagosome
cytosol
pH~ 7.2
ATP
ADP
ATPeV
H+
pH~ 5.0
Transport of
synthesized lysosomal enzymes
(See below)
clathrin coat
early endosome
late endosome
multivesicular body (MVB)
acid hydrolase
lysosomal membrane protein
lysosome
Endocytosis
Endocytosis
MCOLN1
mitochondria
Autophagy
autophagosome
Regulation of autophagy
Glycosaminoglycan degradation
Other glycan degradation
plasma membrane
Lysosomal acid hydrolases
proteases
cathepsins
napsin
LGMN
TPP1
glycosidases
GLA
GLB
GAA
GBA
IDUA
NAGA
NAGLU
GALC
GUSB
FUCA1
HEXA/B
MANB
LAMAN
NEU1
HYAL1
sulfatases
ARS
GALNS
GNS
IDS
SGSH
lipases
nuclease
phosphatase
LIPA
LYPLA3
DNaseII
ACP2
ACP5
sphingomyelinase
ceramidase
aspartylglucosaminidase
SMPD1
ASAH1
AGA
Other lysosomal enzymes and activators
saposin
GM2A
CLN1
Lysosomal membrane proteins
major lysosomal membrane proteins
LAMP
LIMP
minor lysosomal membrane proteins
NPC
cystinosin
sialin
NRAMP
LAPTM
ABCA2
ABCB9
ACP2
endolyn
LALP70
sortilin
CLN3
CLN5
CLN7
HGSNAT
MCOLN1
LITAF
Activation of lysosomal sulfatase precursor
FGE
lysosomal hydrase precursor
from ER
mannose
+p
GNPT
NAGPA
M6P
Snare interactions in vesicular transport
cis Golgi network
M6P receptor
MPR
M6P
trans Golgi network
Golgi body
AP-1
AP-3
GGAs
AP-4
clathrin
Receptor-dependent transport
M6P
transport vesicle
Receptor recycling
AP-1
M6P
M6P
ATPeV
-p
mannose
AP-3
lysosome
late endosome
mature lysosomal hydrase
Transport of synthesized lysosomal enzymes
04142 6/19/15
(c) Kanehisa Laboratories

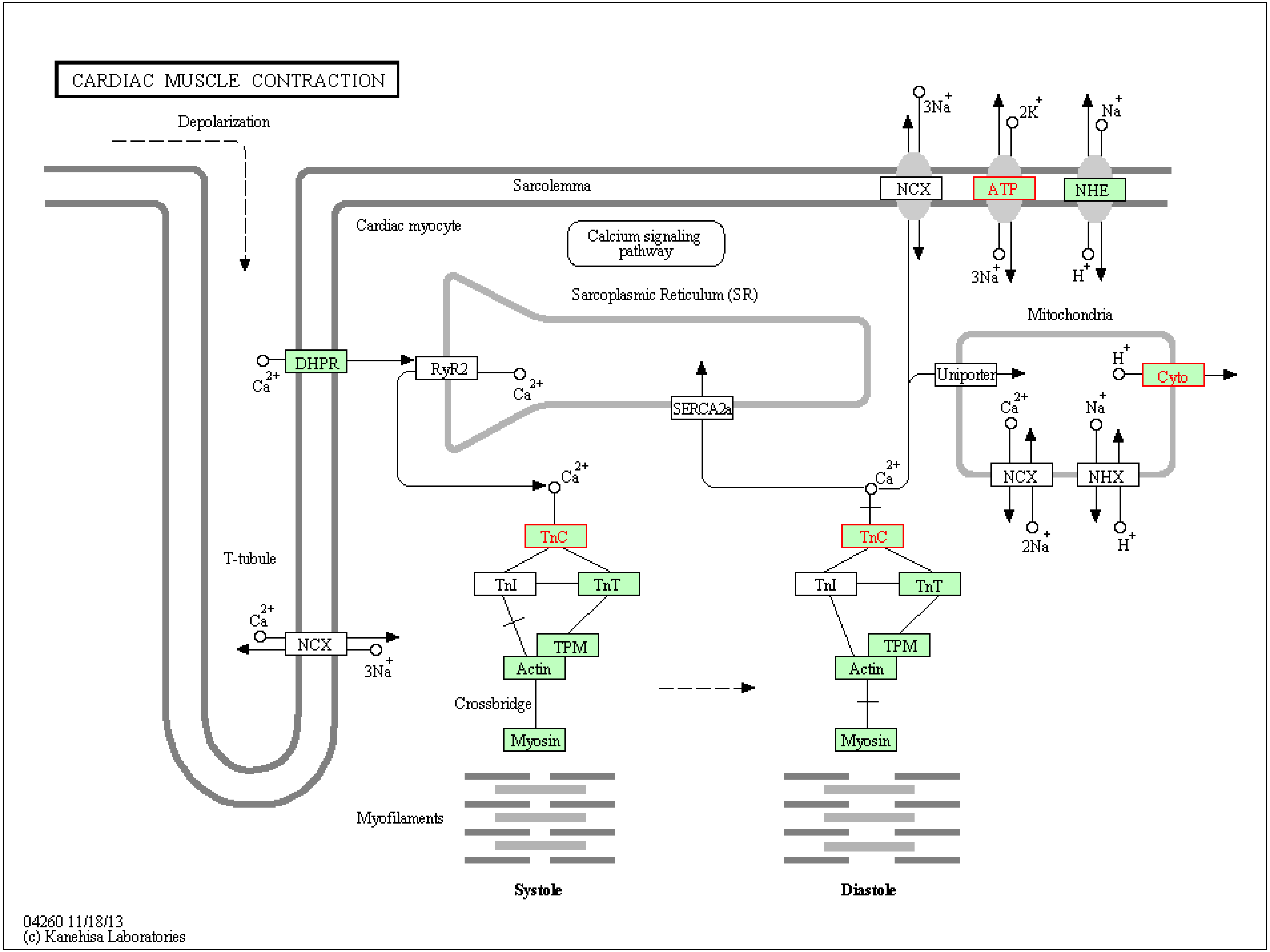
CARDIAC MUSCLE CONTRACTION
Depolarization
Sarcolemma
Cardiac myocyte
Calcium signaling pathway
Sarcoplasmic Reticulum (SR)
3Na+
2K+
Na+
NCX
ATP
NHE
3Na+
H+
Mitochondria
DHPR
Ca2+
RyR2
Ca2+
SERCA2a
Uniporter
H+
Cyto
Ca2+
Na+
NCX
NHX
2Na+
H+
Ca2+
Ca2+
TnC
TnC
T-tubule
TnI
TnT
TnI
TnT
Ca2+
NCX
3Na+
TPM
Actin
TPM
Actin
Crossbridge
Myosin
Myosin
Myofilaments
Systole
Diastole
04260 11/18/13
(c) Kanehisa Laboratories

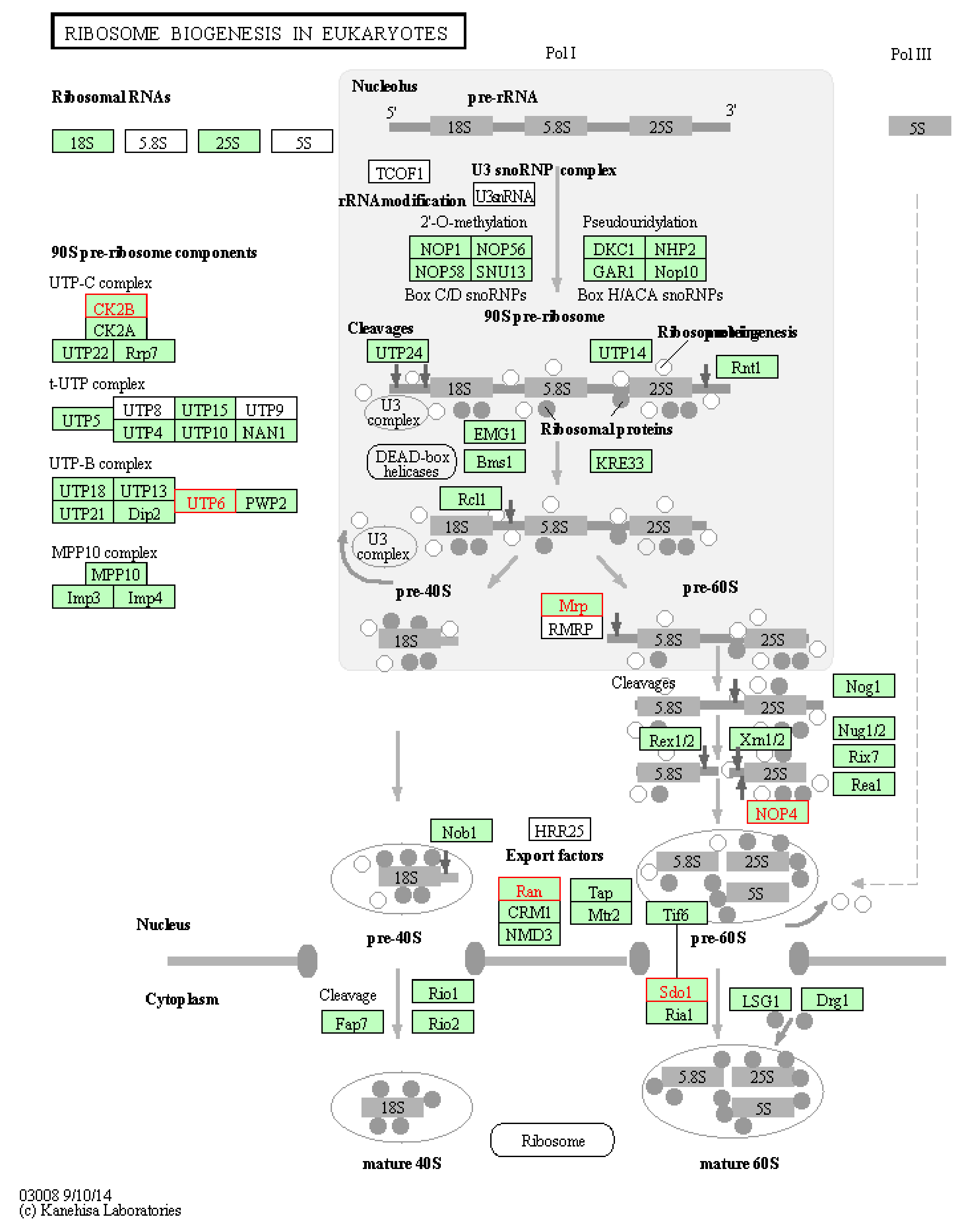

RIBOSOME BIOGENESIS IN EUKARYOTES
Ribosomal RNAs
18S
5.8S
25S
5S
90S pre-ribosome components
UTP-C complex
CK2B
CK2A
UTP22
Rrp7
t-UTP complex
UTP5
UTP8
UTP15
UTP9
UTP4
UTP10
NAN1
UTP-B complex
UTP18
UTP13
UTP21
Dip2
UTP6
PWP2
MPP10 complex
MPP10
Imp3
Imp4
Pol I
Pol III
Nucleolus
pre-rRNA
5'
3'
TCOF1
U3 snoRNP complex
U3snRNA
rRNA modification
2'-O-methylation
Pseudouridylation
NOP1
NOP56
NOP58
SNU13
DKC1
NHP2
GAR1
Nop10
Box C/D snoRNPs
Box H/ACA snoRNPs
90S pre-ribosome
Cleavages
UTP24
UTP14
Rnt1
U3 complex
EMG1
Bms1
KRE33
Ribosomal proteins
DEAD-box helicases
Rcl1
pre-40S
pre-60S
Mrp
RMRP
Nog1
Nug1/2
Rix7
Rea1
Rex1/2
Xrn1/2
NOP4
Nob1
HRR25
Export factors
Ran
CRM1
NMD3
Tap
Mtr2
Tif6
Nucleus
Cytoplasm
Cleavage
Rio1
Fap7
Rio2
Sdo1
Ria1
LSG1
Drg1
Ribosome
mature 40S
mature 60S
03008 9/10/14
(c) Kanehisa Laboratories

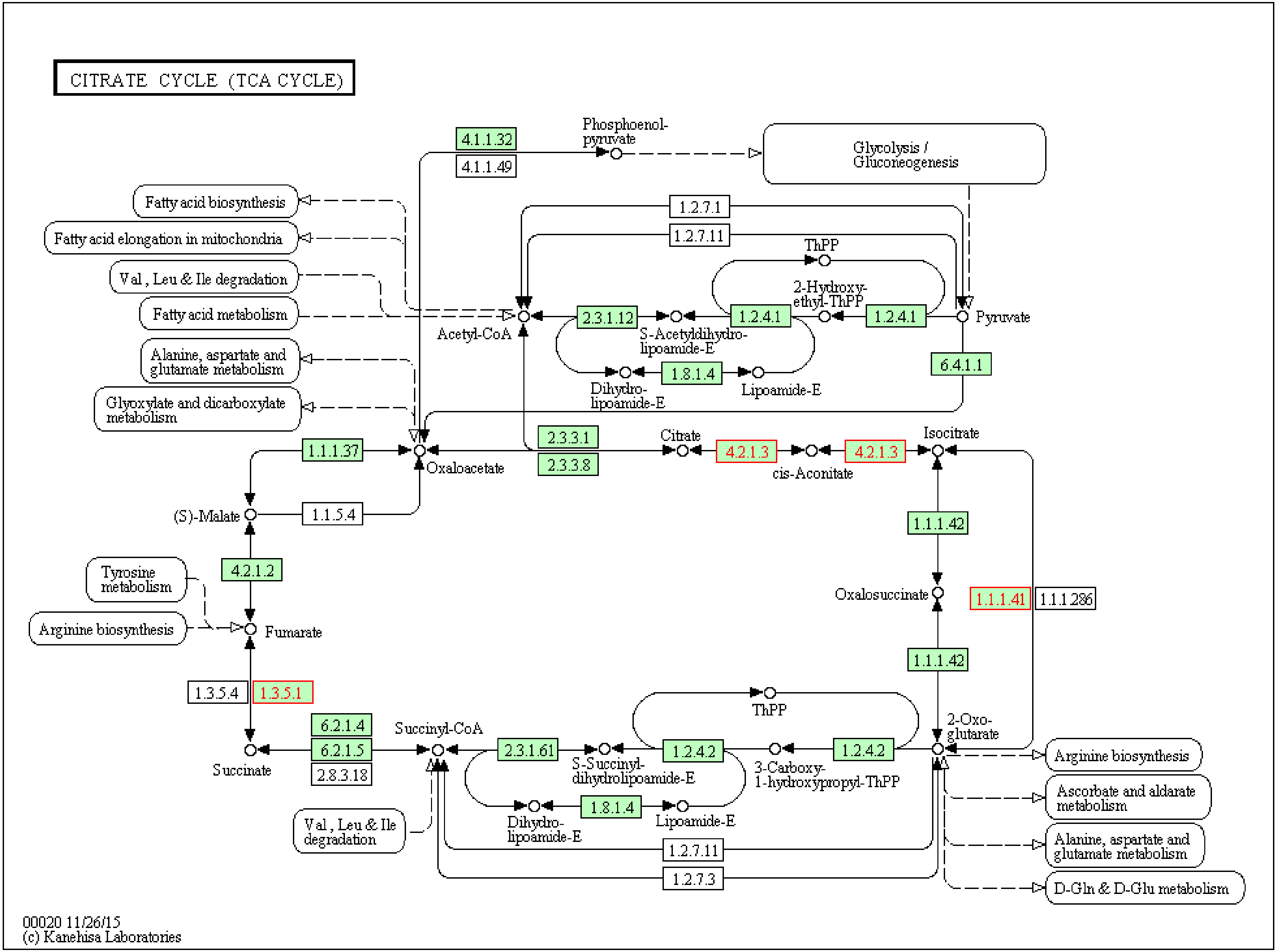
CITRATE CYCLE (TCA CYCLE)
Phosphoenol-pyruvate
Glycolysis / Gluconeogenesis
4.1.1.32
4.1.1.49
Fatty acid biosynthesis
Fatty acid elongation in mitochondria
Val, Leu & Ile degradation
Fatty acid metabolism
Alanine, aspartate and glutamate metabolism
Glyoxylate and dicarboxylate metabolism
1.2.7.1
1.2.7.11
ThPP
2-Hydroxy-ethyl-ThPP
2.3.1.12
1.2.4.1
1.2.4.1
Pyruvate
Acetyl-CoA
S-Acetyldihydro-lipoamide-E
Dihydro-lipoamide-E
1.8.1.4
Lipoamide-E
6.4.1.1
2.3.3.1
2.3.3.8
Citrate
4.2.1.3
4.2.1.3
Isocitrate
cis-Aconitate
1.1.1.37
Oxaloacetate
(S)-Malate
1.1.5.4
1.1.1.42
Oxalosuccinate
1.1.1.41
1.1.1.286
1.1.1.42
Tyrosine metabolism
Arginine biosynthesis
4.2.1.2
Fumarate
1.3.5.4
1.3.5.1
Succinate
6.2.1.4
6.2.1.5
2.8.3.18
Succinyl-CoA
2.3.1.61
S-Succinyl-dihydrolipoamide-E
1.2.4.2
3-Carboxy-1-hydroxypropyl-ThPP
1.2.4.2
ThPP
2-Oxo-glutarate
Dihydro-lipoamide-E
1.8.1.4
Lipoamide-E
Val, Leu & Ile degradation
1.2.7.11
1.2.7.3
Arginine biosynthesis
Ascorbate and aldarate metabolism
Alanine, aspartate and glutamate metabolism
D-Gln & D-Glu metabolism
00020 11/26/15
(c) Kanehisa Laboratories

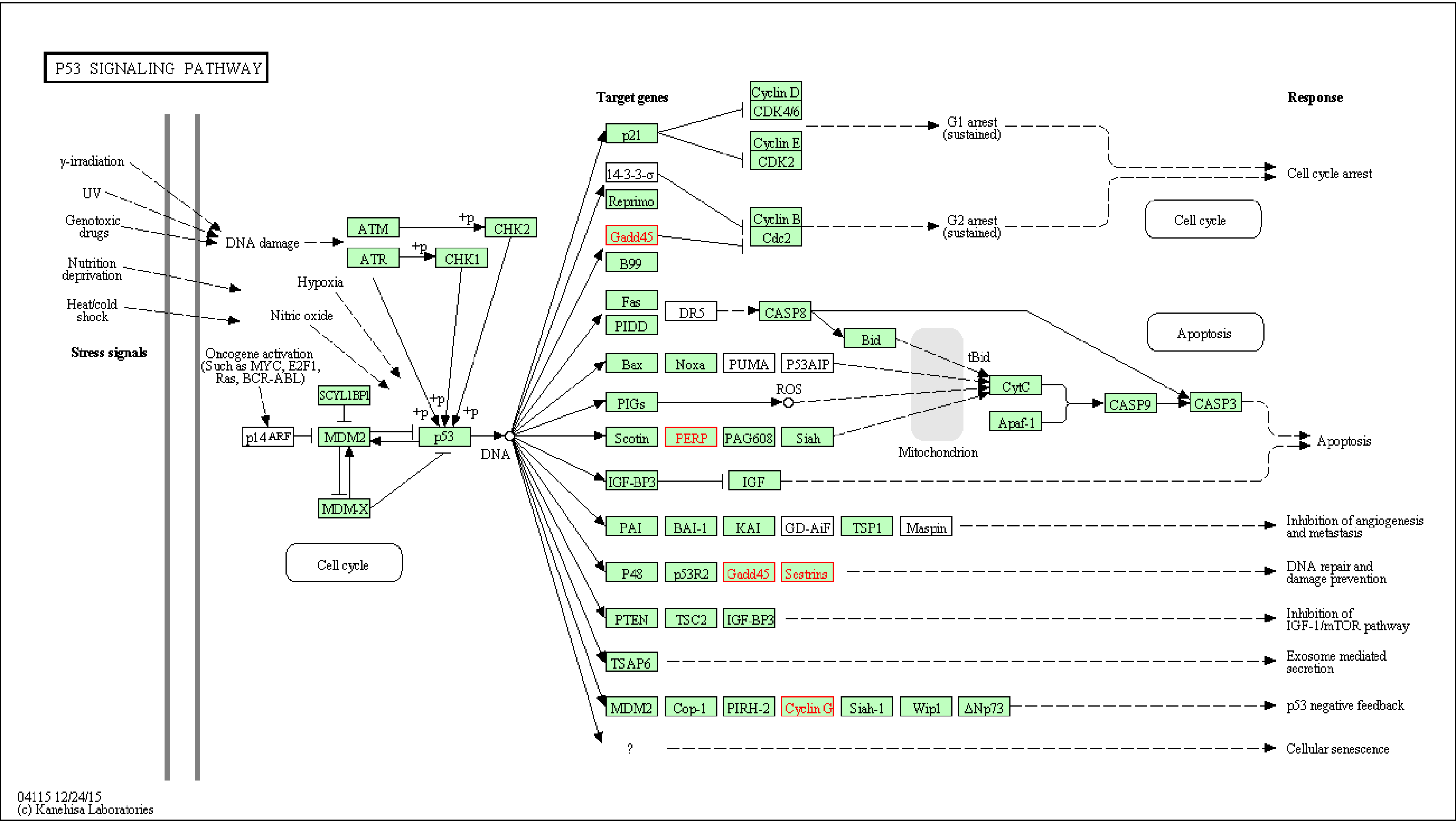
P53 SIGNALING PATHWAY
γ-irradiation
UV
Genotoxic drugs
Nutrition deprivation
Heat/cold shock
Stress signals
DNA damage
ATM
ATR
+p
CHK2
CHK1
Hypoxia
Nitric oxide
Oncogene activation (Such as MYC, E2F1, Ras, BCR-ABL)
SCYL1BP1
p14ARF
MDM2
p53
+p
DNA
MDM-X
Cell cycle
Target genes
p21
14-3-3-σ
Reprimo
Gadd45
B99
Cyclin D
CDK4/6
Cyclin E
CDK2
Cyclin B
Cdc2
G1 arrest (sustained)
G2 arrest (sustained)
Response
Cell cycle arrest
Cell cycle
Fas
PIDD
DR5
CASP8
Bid
tBid
Apoptosis
Bax
Noxa
PUMA
P53AIP
CytC
PIGs
ROS
Apaf-1
CASP9
CASP3
Scotin
PERP
PAG608
Siah
Mitochondrion
Apoptosis
IGF-BP3
IGF
PAI
BAI-1
KAI
GD-AiF
TSP1
Maspin
Inhibition of angiogenesis and metastasis
P48
p53R2
Gadd45
Sestrins
DNA repair and damage prevention
PTEN
TSC2
IGF-BP3
Inhibition of IGF-1/mTOR pathway
TSAP6
Exosome mediated secretion
MDM2
Cop-1
PIRH-2
Cyclin G
Siah-1
Wip1
ΔNp73
p53 negative feedback
?
Cellular senescence
04115 12/24/15
(c) Kanehisa Laboratories

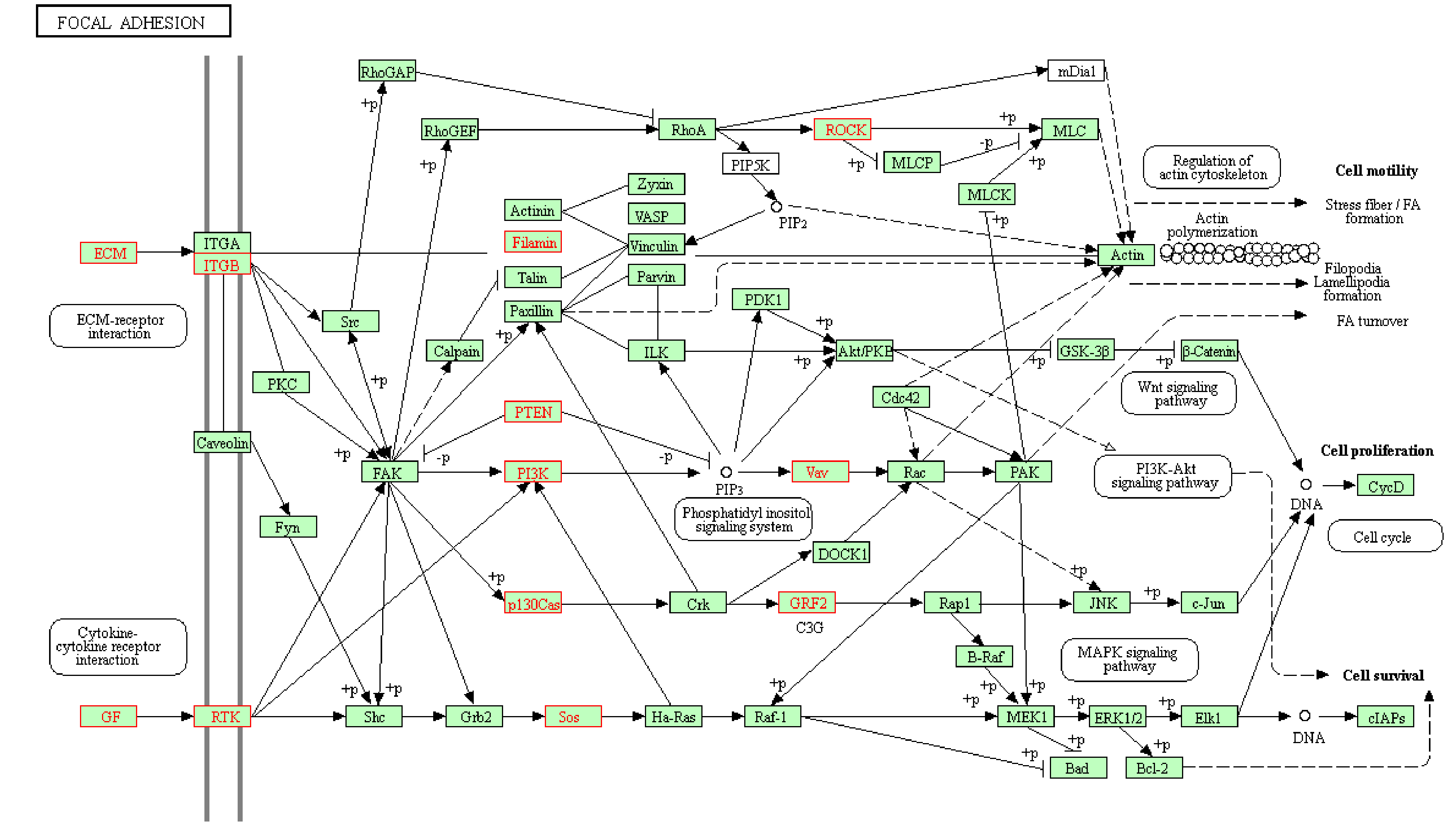

04510 11/25/15

(c) Kanehisa Laboratories

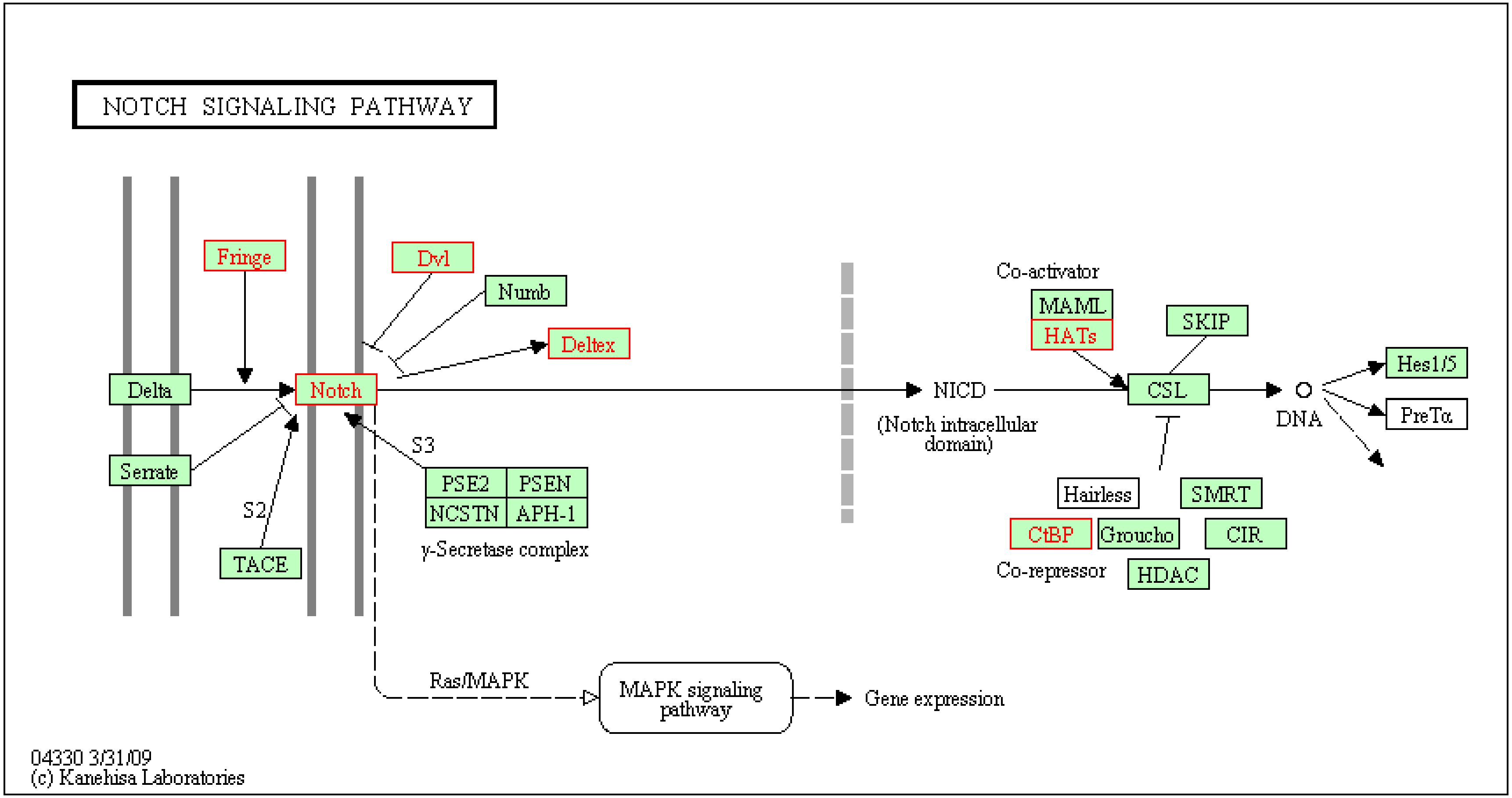
NOTCH SIGNALING PATHWAY
Fringe
Dvl
Numb
Deltex
Delta
Notch
Serrate
S2
TACE
S3
PSE2
PSEN
NCSTN
APH-1
γ-Secretase complex
NICD
(Notch intracellular domain)
Co-activator
MAML
HATs
SKIP
CSL
DNA
Hes1/5
PreTα
Hairless
SMRT
CtBP
Groucho
CIR
Co-repressor
HDAC
Ras/MAPK
MAPK signaling pathway
Gene expression
04330 3/31/09
(c) Kanehisa Laboratories

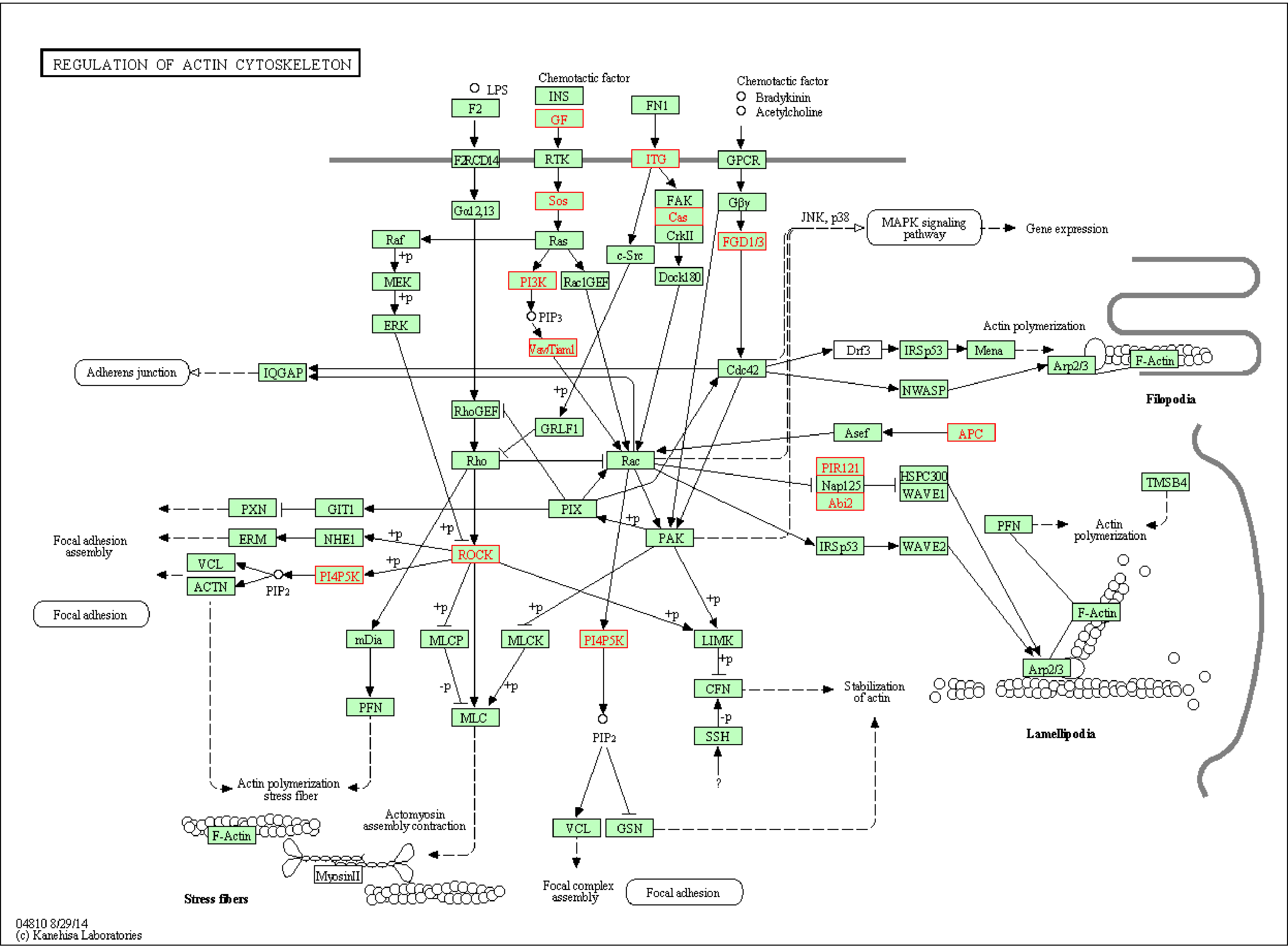
REGULATION OF ACTIN CYTOSKELETON
LPS
F2
Chemotactic factor
INS
GF
FN1
Chemotactic factor
Bradykinin
Acetylcholine
F2RCD14
RTK
ITG
GPCR
Gα12,13
Sos
FAK
Cas
CrkII
Gβγ
JNK, p38
MAPK signaling pathway
Gene expression
Raf
Ras
c-Src
FGD1/3
MEK
PI3K
Rac1GEF
Dock180
ERK
PIP3
Vav/Tiam1
Actin polymerization
Drf3
IRSp53
Mena
Arp2/3
F-Actin
Adherens junction
IQGAP
Cdc42
NWASP
Filopodia
RhoGEF
GRLF1
Asef
APC
Rho
Rac
PIR121
Nap125
Abi2
HSPC300
WAVE1
TMSB4
PXN
GIT1
PIX
PAK
PFN
Actin polymerization
Focal adhesion assembly
ERM
NHE1
ROCK
IRSp53
WAVE2
VCL
ACTN
PI4P5K
PIP2
Focal adhesion
mDia
MLCP
MLCK
PI4P5K
LIMK
F-Actin
Arp2/3
CFN
Stabilization of actin
PFN
MLC
SSH
PIP2
Lamellipodia
?
Actin polymerization stress fiber
Actomyosin assembly contraction
F-Actin
MyosinII
VCL
GSN
Stress fibers
Focal complex assembly
Focal adhesion
04810 8/29/14
(c) Kanehisa Laboratories

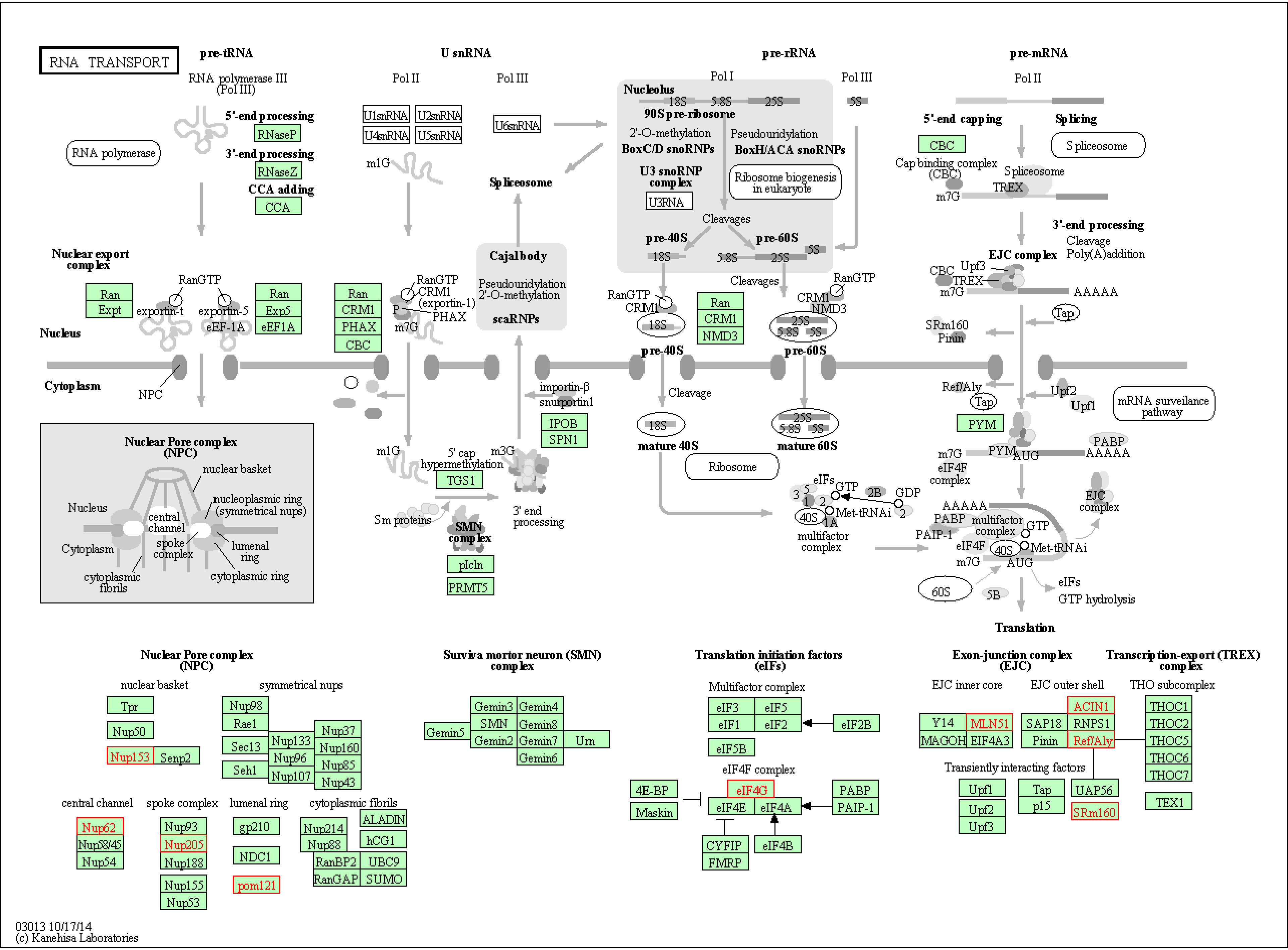
RNA TRANSPORT
pre-tRNA
RNA polymerase III (Pol III)
5'-end processing
RNaseP
3'-end processing
RNaseZ
CCA adding
CCA
RNA polymerase
U snRNA
Pol II
Pol III
U1snRNA
U2snRNA
U4snRNA
U5snRNA
U6snRNA
m1G
Spliceosome
pre-rRNA
Pol I
Pol III
Nucleolus
18S
5.8S
25S
5S
90S pre-ribosome
2'-O-methylation
BoxC/D snoRNPs
Pseudouridylation
BoxH/ACA snoRNPs
U3 snoRNP complex
U3RNA
Ribosome biogenesis in eukaryote
Cleavages
pre-40S
pre-60S
pre-mRNA
Pol II
5'-end capping
CBC
Splicing
Spliceosome
Cap binding complex (CBC)
Spliceosome
TREX
m7G
3'-end processing
Cleavage
Poly(A)addition
EJC complex
CBC
Upf3
TREX
m7G
AAAAA
Tap
SRm160
Pinin
Nuclear export complex
Ran
Expt
RanGTP
exportin-t
exportin-5
eEF-1A
Ran
Exp5
eEF1A
Nucleus
Ran
CRM1
PHAX
CBC
RanGTP
CRM1
(exportin-1)
P
PHAX
m7G
Cajal body
Pseudouridylation
2'-O-methylation
scaRNPs
RanGTP
CRM1
18S
pre-40S
Ran
CRM1
NMD3
Cleavages
RanGTP
CRM1
NMD3
25S
5.8S
5S
pre-60S
Cytoplasm
NPC
importin-β
snurportin1
IPOB
SPN1
Cleavage
18S
mature 40S
25S
5.8S
5S
mature 60S
Ribosome
Ref/Aly
Tap
PYM
Upf2
Upf1
mRNA surveilance pathway
m7G
PYM
AUG
PABP
AAAAA
eIF4F complex
EJC complex
Nuclear Pore complex (NPC)
nuclear basket
Nucleus
central channel
nucleoplasmic ring (symmetrical nups)
Cytoplasm
spoke complex
lumenal ring
cytoplasmic fibrils
cytoplasmic ring
m1G
5' cap hypermethylation
m3G
TGS1
3' end processing
Sm proteins
SMN complex
pICln
PRMT5
eIFs
GTP
2B
GDP
3
5
1
2
40S
1A
Met-tRNAi
2
multifactor complex
AAAAA
PABP
multifactor complex
GTP
PAIP-1
eIF4F
40S
Met-tRNAi
m7G
AUG
60S
5B
eIFs
GTP hydrolysis
Translation
Nuclear Pore complex (NPC)
nuclear basket
Tpr
Nup50
Nup153
Senp2
symmetrical nups
Nup98
Rae1
Sec13
Seh1
Nup133
Nup96
Nup107
Nup37
Nup160
Nup85
Nup43
central channel
Nup62
Nup58/45
Nup54
spoke complex
Nup93
Nup205
Nup188
Nup155
Nup53
lumenal ring
gp210
NDC1
pom121
cytoplasmic fibrils
Nup214
Nup88
ALADIN
hCG1
RanBP2
UBC9
RanGAP
SUMO
Surviva mortor neuron (SMN) complex
Gemin3
Gemin4
SMN
Gemin8
Gemin5
Gemin2
Gemin7
Urn
Gemin6
Translation initiation factors (eIFs)
Multifactor complex
eIF3
eIF5
eIF1
eIF2
eIF2B
eIF5B
eIF4F complex
4E-BP
Maskin
eIF4G
eIF4E
eIF4A
PABP
PAIP-1
CYFIP
FMRP
eIF4B
Exon-junction complex (EJC)
EJC inner core
Y14
MLN51
MAGOH
EIF4A3
EJC outer shell
ACIN1
SAP18
RNPS1
Pinin
Ref/Aly
Transiently interacting factors
Upf1
Upf2
Upf3
Tap
p15
UAP56
SRm160
Transcription-export (TREX) complex
THO subcomplex
THOC1
THOC2
THOC5
THOC6
THOC7
TEX1
03013 10/17/14
(c) Kanehisa Laboratories

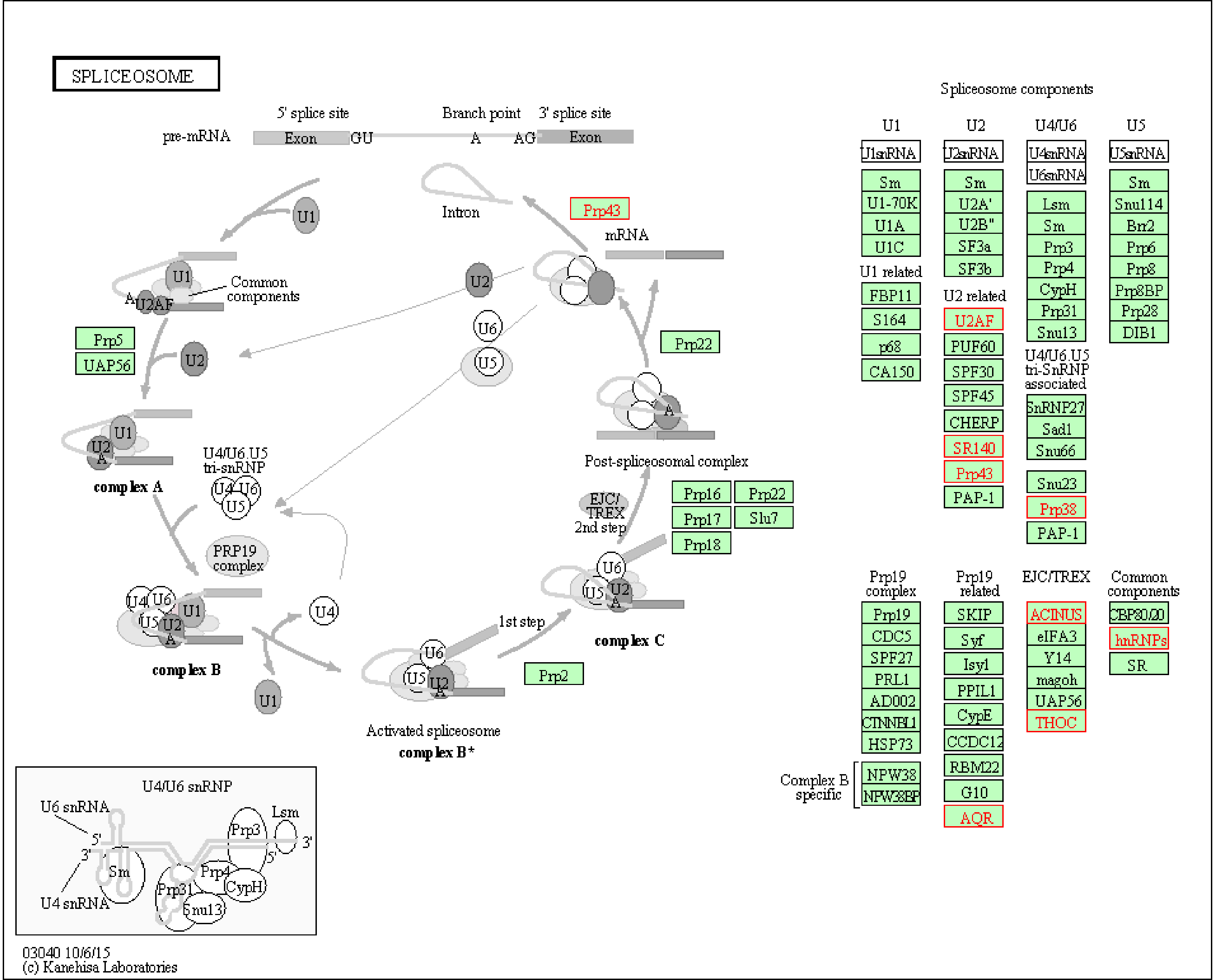

SPLICEOSOME
5' splice site
Branch point
3' splice site
pre-mRNA
Exon
GU
A
AG
Exon
U1
Intron
Prp43
mRNA
U1
Common components
A
U2AF
U2
U6
U5
Prp22
Prp5
UAP56
U2
U1
U2
A
complex A
U4/U6.U5 tri-snRNP
U4
U6
U5
Post-spliceosomal complex
EJC/TREX
2nd step
Prp16
Prp22
Prp17
Slu7
Prp18
PRP19 complex
U6
U5
U2
A
U4
U6
U1
U5
U2
A
complex B
U4
1st step
complex C
U6
U5
U2
A
Prp2
U1
Activated spliceosome
complex B*
U4/U6 snRNP
U6 snRNA
Lsm
Prp3
5'
3'
3'
5'
Sm
Prp4
CypH
Prp31
Snu13
U4 snRNA
Spliceosome components
U1
U1snRNA
Sm
U1-70K
U1A
U1C
U1 related
FBP11
S164
p68
CA150
U2
U2snRNA
Sm
U2A'
U2B"
SF3a
SF3b
U2 related
U2AF
PUF60
SPF30
SPF45
CHERP
SR140
Prp43
PAP-1
U4/U6
U4snRNA
U6snRNA
Lsm
Sm
Prp3
Prp4
CypH
Prp31
Snu13
U4/U6.U5 tri-SnRNP associated
SnRNP27
Sad1
Snu66
Snu23
Prp38
PAP-1
U5
U5snRNA
Sm
Snu114
Brr2
Prp6
Prp8
Prp8BP
Prp28
DIB1
Prp19 complex
Prp19
CDC5
SPF27
PRL1
AD002
CTNNBL1
HSP73
Complex B specific
NPW38
NPW38BP
Prp19 related
SKIP
Syf
Isy1
PPIL1
CypE
CCDC12
RBM22
G10
AQR
EJC/TREX
ACINUS
eIFA3
Y14
magoh
UAP56
THOC
Common components
CBP80/20
hnRNPs
SR
03040 10/6/15
(c) Kanehisa Laboratories

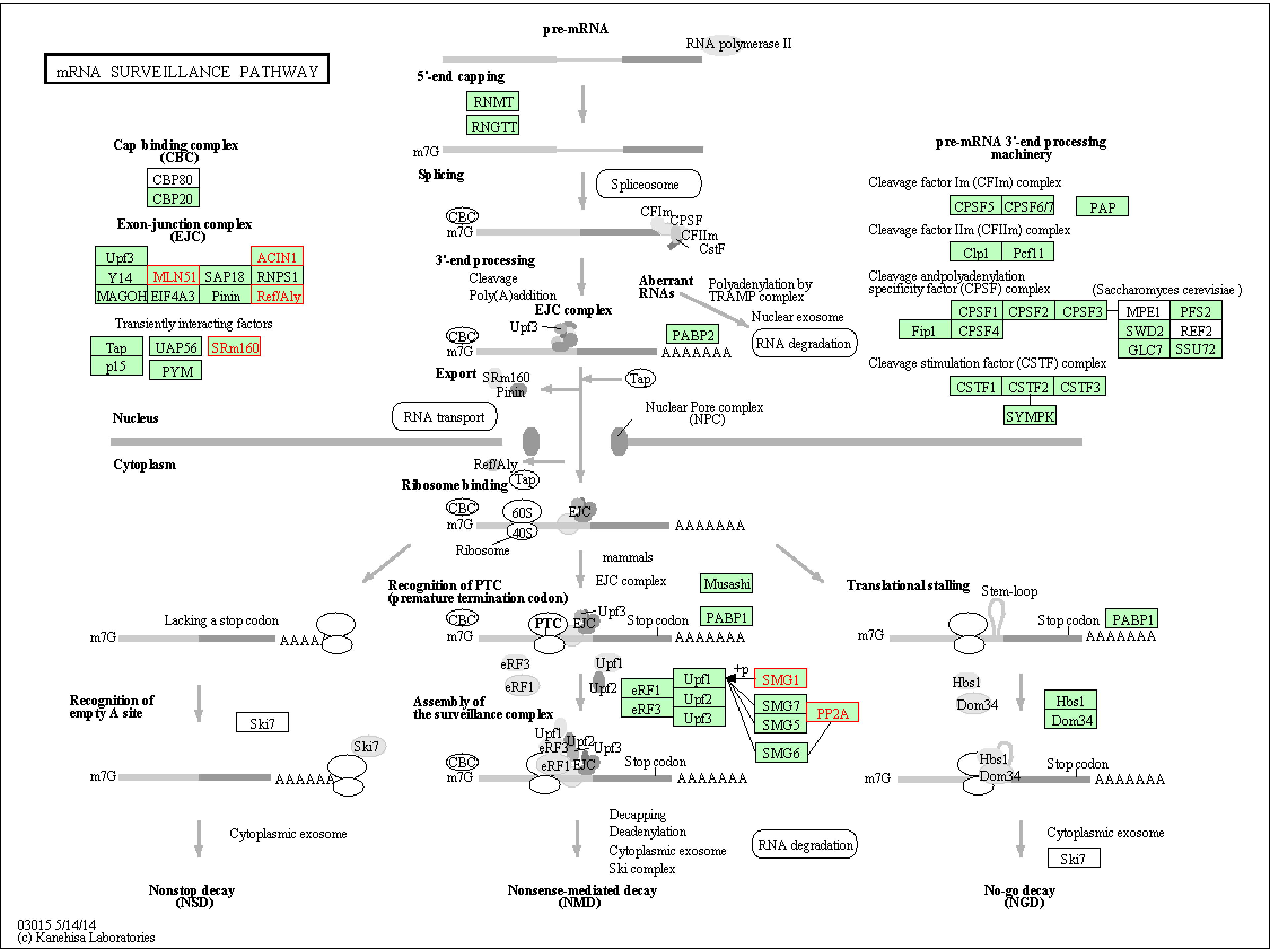
mRNA SURVEILLANCE PATHWAY
pre-mRNA
RNA polymerase II
5'-end capping
RNMT
RNGTT
m7G
Splicing
Spliceosome
Cap binding complex (CBC)
CBP80
CBP20
Exon-junction complex (EJC)
Upf3
ACIN1
Y14
MLN51
SAP18
RNPS1
MAGOH
EIF4A3
Pinin
Ref/Aly
Transiently interacting factors
Tap
p15
UAP56
SRm160
PYM
CBC
CFIm
CPSF
CFIIm
CstF
3'-end processing
Cleavage
Poly(A)addition
Aberrant RNAs
Polyadenylation by TRAMP complex
Nuclear exosome
RNA degradation
EJC complex
PABP2
AAAAAAA
Export
SRm160
Pinin
Tap
RNA transport
Nuclear Pore complex (NPC)
Nucleus
Cytoplasm
Ref/Aly
Ribosome binding
60S
40S
EJC
Ribosome
pre-mRNA 3'-end processing machinery
Cleavage factor Im (CFIm) complex
CPSF5
CPSF6/7
PAP
Cleavage factor IIm (CFIIm) complex
Clp1
Pcf11
Cleavage andpolyadenylation specificity factor (CPSF) complex
(Saccharomyces cerevisiae)
CPSF1
CPSF2
CPSF3
MPE1
PFS2
Fip1
CPSF4
SWD2
REF2
GLC7
SSU72
Cleavage stimulation factor (CSTF) complex
CSTF1
CSTF2
CSTF3
SYMPK
mammals
EJC complex
Musashi
PABP1
Recognition of PTC (premature termination codon)
PTC
Upf3
Stop codon
Lacking a stop codon
AAAA
Translational stalling
Stem-loop
Stop codon
PABP1
eRF3
eRF1
Upf1
Upf2
eRF1
eRF3
Upf1
Upf2
Upf3
+p
SMG1
SMG7
SMG5
PP2A
SMG6
Assembly of the surveillance complex
Recognition of empty A site
Ski7
Ski7
AAAAAA
Upf1
eRF3
Upf2
Upf3
eRF1
EJC
Stop codon
Hbs1
Dom34
Hbs1
Dom34
Hbs1
Dom34
Stop codon
Cytoplasmic exosome
Decapping
Deadenylation
Cytoplasmic exosome
Ski complex
RNA degradation
Cytoplasmic exosome
Ski7
Nonstop decay (NSD)
Nonsense-mediated decay (NMD)
No-go decay (NGD)
03015 5/14/14
(c) Kanehisa Laboratories

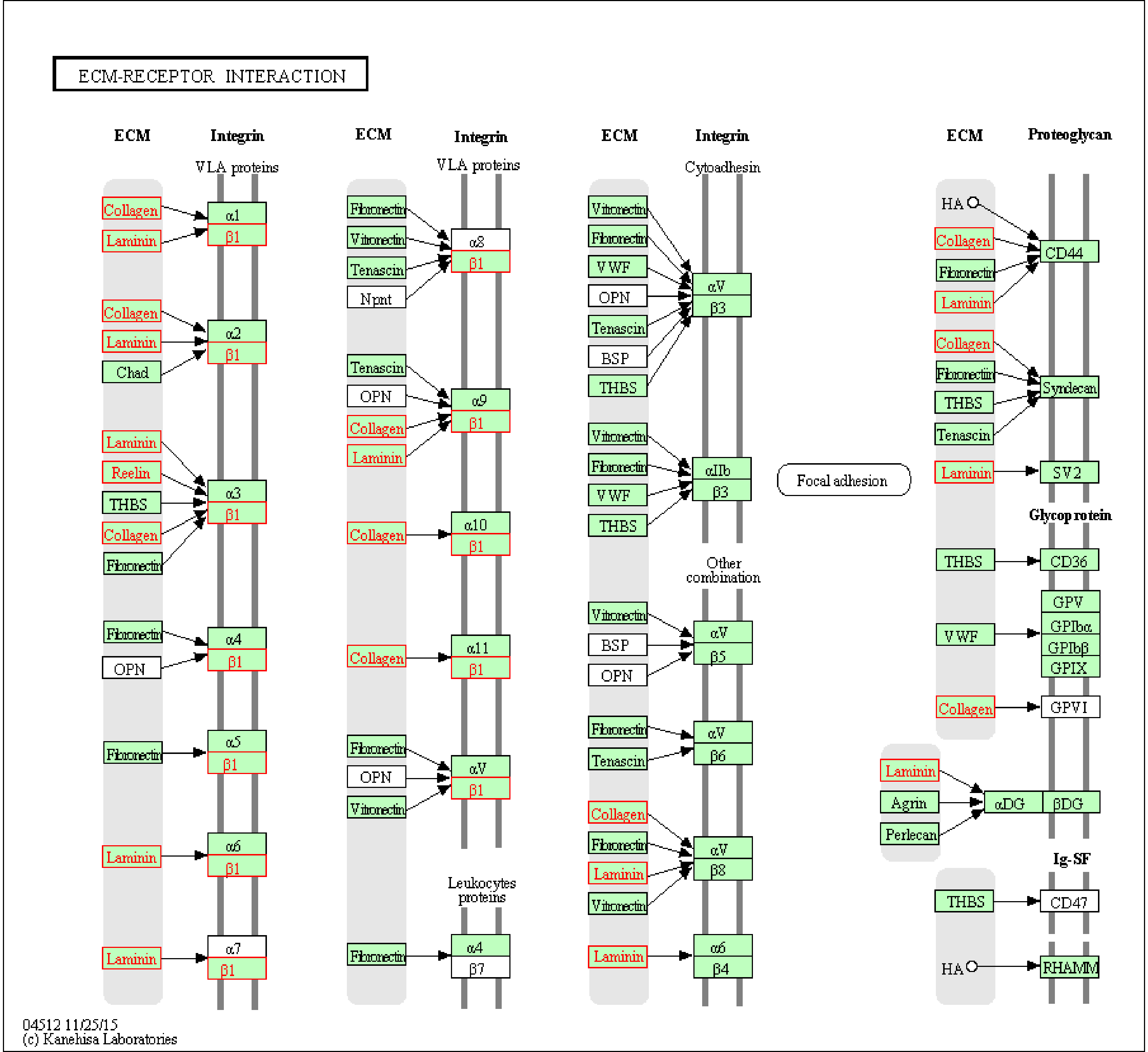
ECM-RECEPTOR INTERACTION
ECM
Integrin
VLA proteins
Collagen
Laminin
α1
β1
Collagen
Laminin
Chad
α2
β1
Laminin
Reelin
THBS
Collagen
Fibronectin
α3
β1
Fibronectin
OPN
α4
β1
Fibronectin
α5
β1
Laminin
α6
β1
Laminin
α7
β1
ECM
Integrin
VLA proteins
Fibronectin
Vitronectin
Tenascin
Npnt
α8
β1
Tenascin
OPN
Collagen
Laminin
α9
β1
Collagen
α10
β1
Collagen
α11
β1
Fibronectin
OPN
Vitronectin
αV
β1
Leukocytes proteins
Fibronectin
α4
β7
ECM
Integrin
Cytoadhesin
Vitronectin
Fibronectin
VWF
OPN
Tenascin
BSP
THBS
αV
β3
Vitronectin
Fibronectin
VWF
THBS
αIIb
β3
Other combination
Vitronectin
BSP
OPN
αV
β5
Fibronectin
Tenascin
αV
β6
Collagen
Fibronectin
Laminin
Vitronectin
αV
β8
Laminin
α6
β4
Focal adhesion
ECM
Proteoglycan
HA
Collagen
Fibronectin
Laminin
CD44
Collagen
Fibronectin
THBS
Tenascin
Syndecan
Laminin
SV2
Glycoprotein
THBS
CD36
VWF
GPV
GPIbα
GPIbβ
GPIX
Collagen
GPVI
Laminin
Agrin
Perlecan
αDG
βDG
Ig-SF
THBS
CD47
HA
RHAMM
04512 11/25/15
(c) Kanehisa Laboratories

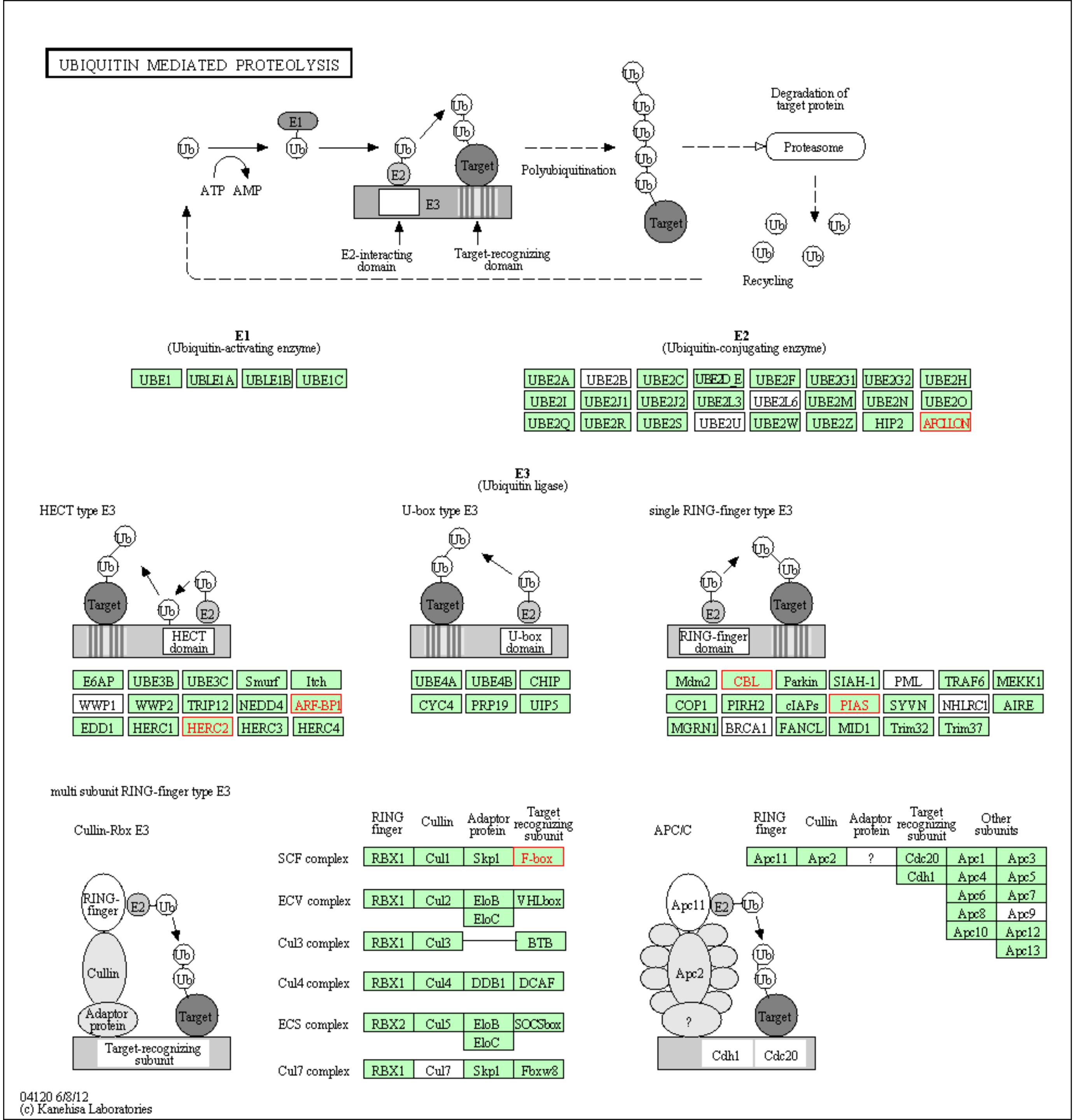
UBIQUITIN MEDIATED PROTEOLYSIS
Ub
E1
ATP AMP
E2
E3
Target
E2-interacting domain
Target-recognizing domain
Polyubiquitination
Degradation of target protein
Proteasome
Recycling
E1
(Ubiquitin-activating enzyme)
UBE1 UBLE1A UBLE1B UBE1C
E2
(Ubiquitin-conjugating enzyme)
UBE2A UBE2B UBE2C UBE2D E UBE2F UBE2G1 UBE2G2 UBE2H
UBE2I UBE2J1 UBE2J2 UBE2L3 UBE2L6 UBE2M UBE2N UBE2O
UBE2Q UBE2R UBE2S UBE2U UBE2W UBE2Z HIP2 APOLLON
E3
(Ubiquitin ligase)
HECT type E3
HECT domain
E6AP UBE3B UBE3C Smurf Itch
WWP1 WWP2 TRIP12 NEDD4 ARF-BP1
EDD1 HERC1 HERC2 HERC3 HERC4
U-box type E3
U-box domain
UBE4A UBE4B CHIP
CYC4 PRP19 UIP5
single RING-finger type E3
RING-finger domain
Mdm2 CBL Parkin SIAH-1 PML TRAF6 MEKK1
COP1 PIRH2 cIAPs PIAS SYVN NHLRC1 AIRE
MGRN1 BRCA1 FANCL MID1 Trim32 Trim37
multi subunit RING-finger type E3
Cullin-Rbx E3
RING-finger
Cullin
Adaptor protein
Target-recognizing subunit
RING finger | Cullin | Adaptor protein | Target recognizing subunit
SCF complex RBX1 Cul1 Skp1 F-box
ECV complex RBX1 Cul2 EloB EloC VHLbox
Cul3 complex RBX1 Cul3 BTB
Cul4 complex RBX1 Cul4 DDB1 DCAF
ECS complex RBX2 Cul5 EloB EloC SOCSbox
Cul7 complex RBX1 Cul7 Skp1 Fbxw8
APC/C
RING finger | Cullin | Adaptor protein | Target recognizing subunit | Other subunits
Apc11 Apc2 ? Cdc20 Cdh1
Apc1 Apc3 Apc4 Apc5 Apc6 Apc7 Apc8 Apc9 Apc10 Apc12 Apc13
Apc11
Apc2
?
Cdh1 Cdc20
04120 6/8/12
(c) Kanehisa Laboratories

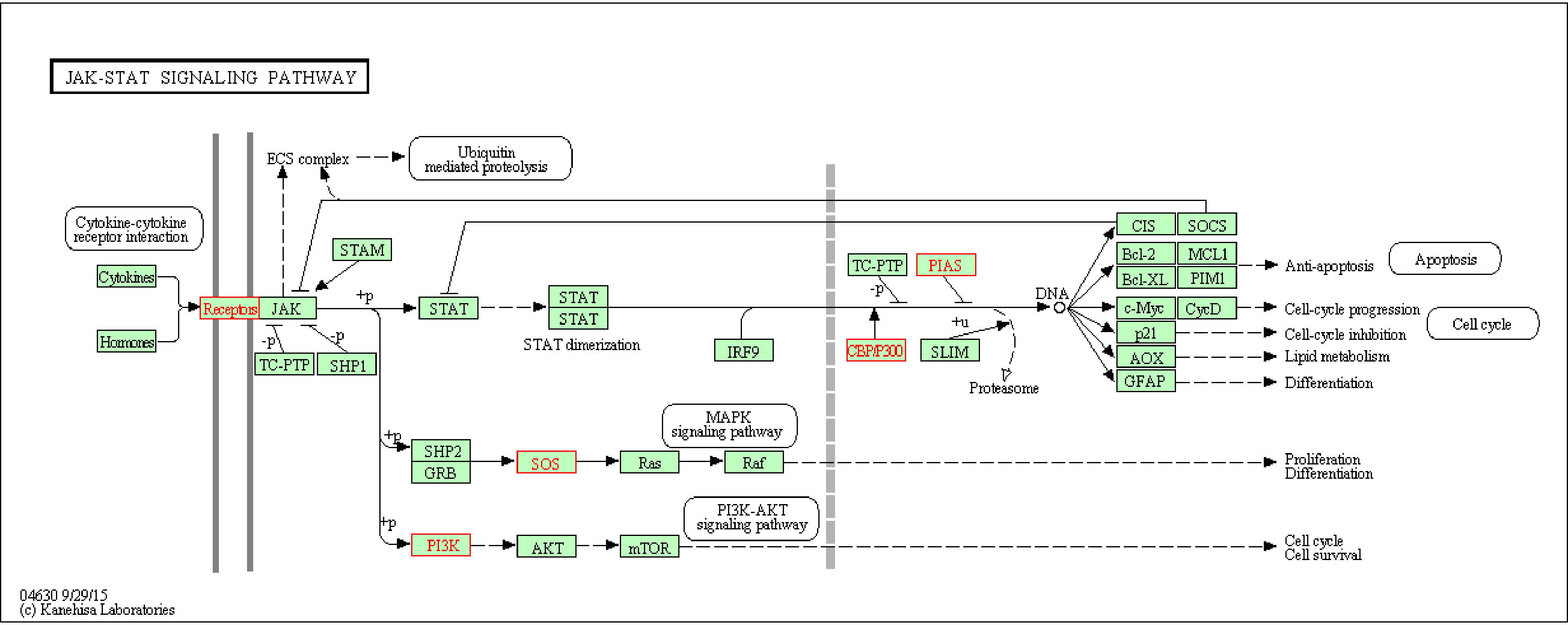
JAK-STAT SIGNALING PATHWAY
ECS complex
Ubiquitin mediated proteolysis
Cytokine-cytokine receptor interaction
Cytokines
Hormones
Receptors
JAK
STAM
+p
-p
TC-PTP
SHP1
STAT
STAT
STAT
STAT dimerization
IRF9
TC-PTP
PIAS
CBP/P300
SLIM
+u
Proteasome
DNA
CIS
SOCS
Bcl-2
MCL1
Bcl-XL
PIM1
c-Myc
CycD
p21
AOX
GFAP
Anti-apoptosis
Apoptosis
Cell-cycle progression
Cell cycle
Cell-cycle inhibition
Lipid metabolism
Differentiation
MAPK signaling pathway
SHP2
GRB
SOS
Ras
Raf
Proliferation
Differentiation
PI3K-AKT signaling pathway
PI3K
AKT
mTOR
Cell cycle
Cell survival
04630 9/29/15
(c) Kanehisa Laboratories

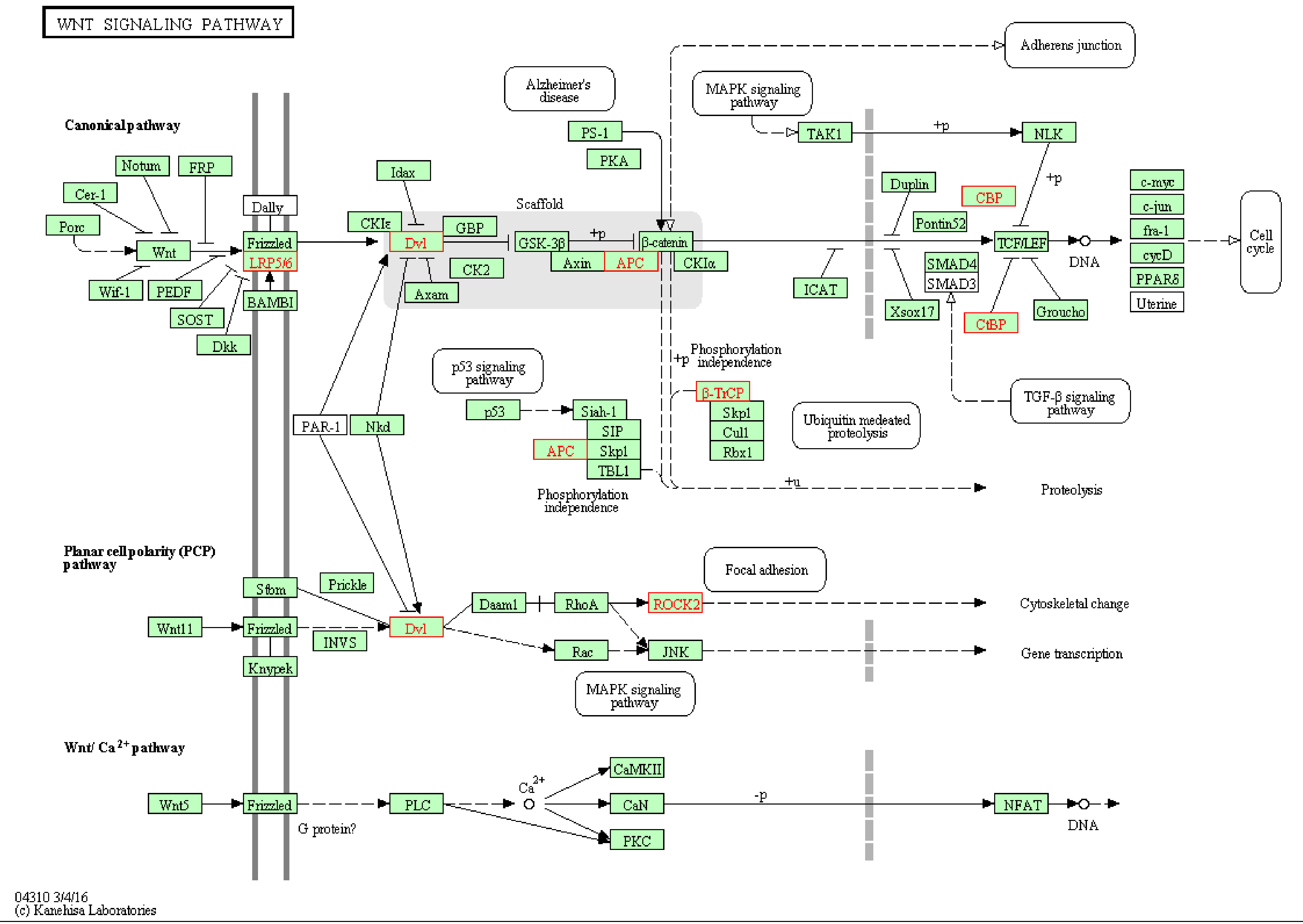

WNT SIGNALING PATHWAY
Canonical pathway
Notum
FRP
Cer-1
Dally
Porc
Wnt
Frizzled
LRP5/6
Wif-1
PEDF
BAMBI
SOST
Dkk
Idax
CKIε
GBP
Dvl
Axam
CK2
Scaffold
GSK-3β
+p
β-catenin
Axin
APC
CKIα
Alzheimer's disease
PS-1
PKA
MAPK signaling pathway
TAK1
+p
NLK
+p
Adherens junction
Duplin
CBP
Pontin52
TCF/LEF
DNA
ICAT
SMAD4
SMAD3
Xsox17
CtBP
Groucho
c-myc
c-jun
fra-1
cycD
PPARδ
Uterine
Cell cycle
p53 signaling pathway
p53
Siah-1
SIP
APC
Skp1
TBL1
Phosphorylation independence
+p
Phosphorylation independence
β-TrCP
Skp1
Cul1
Rbx1
Ubiquitin mediated proteolysis
TGF-β signaling pathway
+u
Proteolysis
PAR-1
Nkd
Planar cell polarity (PCP) pathway
Sfbm
Prickle
Wnt11
Frizzled
INVS
Dvl
Knypek
Daam1
RhoA
ROCK2
Focal adhesion
Cytoskeletal change
Rac
JNK
Gene transcription
MAPK signaling pathway
Wnt/ Ca2+ pathway
Wnt5
Frizzled
PLC
Ca2+
CaMKII
CaN
PKC
-p
NFAT
DNA
G protein?
04310 3/4/16
(c) Kanehisa Laboratories

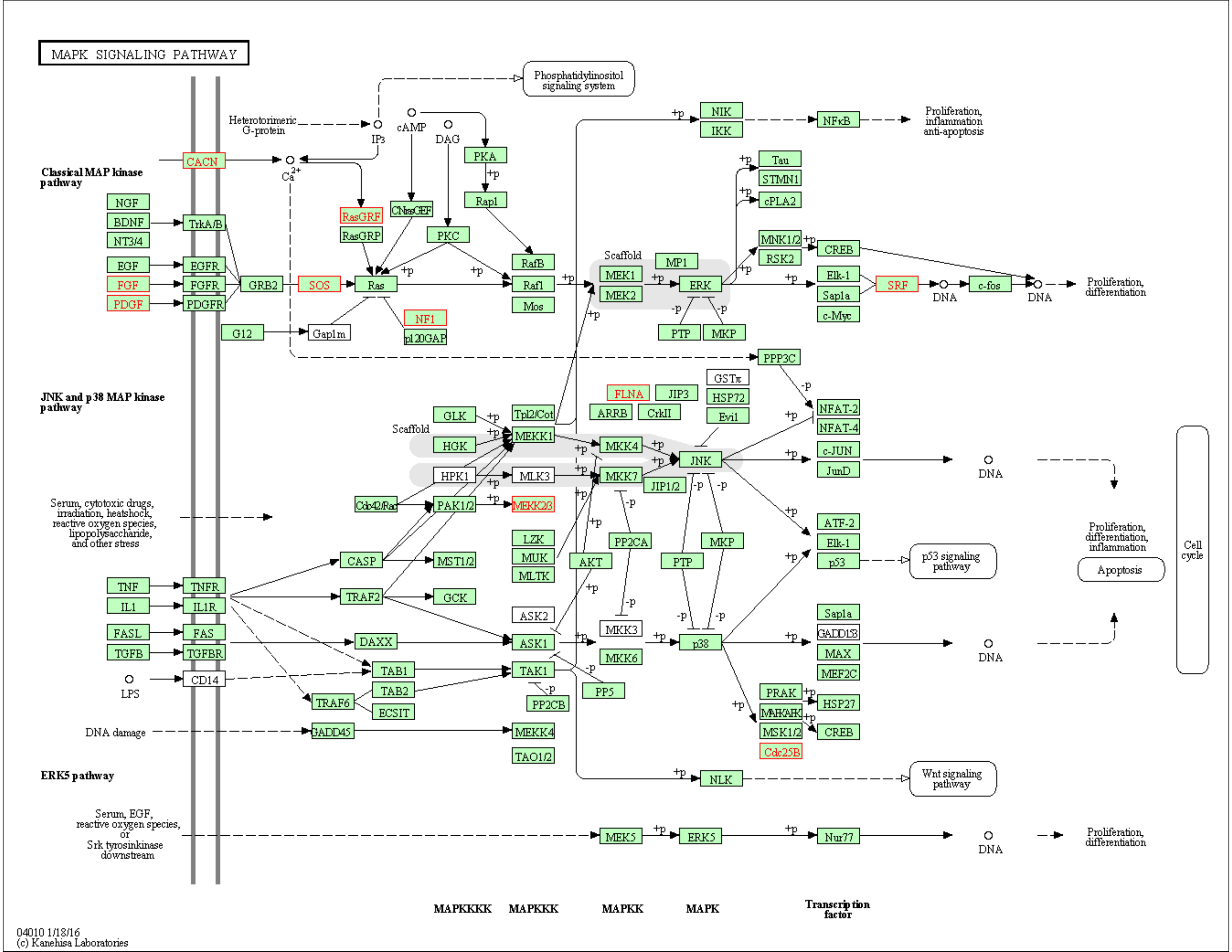
MAPK SIGNALING PATHWAY
Phosphatidylinositol signaling system
Heterotorimeric G-protein
IP3
cAMP
DAG
CACN
Ca2+
Classical MAP kinase pathway
NGF
BDNF
NT3/4
TrkA/B
EGF
EGFR
FGF
FGFR
PDGF
PDGFR
GRB2
SOS
Ras
RasGRF
RasGRP
CNrasGEF
PKC
PKA
Rap1
RafB
Raf1
Mos
NF1
p120GAP
G12
Gap1m
Scaffold
MEK1
MEK2
MP1
ERK
PTP
MKP
NIK
IKK
NFκB
Proliferation, inflammation anti-apoptosis
Tau
STMN1
cPLA2
MNK1/2
RSK2
CREB
Elk-1
Sap1a
c-Myc
SRF
DNA
c-fos
Proliferation, differentiation
PPP3C
NFAT-2
NFAT-4
JNK and p38 MAP kinase pathway
GLK
HGK
HPK1
Tpl2/Cot
MEKK1
MLK3
MEKK2/3
PAK1/2
Cdc42/Rac
FLNA
JIP3
GSTπ
HSP72
Evi1
ARRB
CrkII
MKK4
MKK7
JIP1/2
JNK
c-JUN
JunD
ATF-2
p53
p53 signaling pathway
Serum, cytotoxic drugs, irradiation, heatshock, reactive oxygen species, lipopolysaccharide, and other stress
CASP
MST1/2
TRAF2
GCK
LZK
MUK
MLTK
AKT
PP2CA
PTP
MKP
TNF
TNFR
IL1
IL1R
FASL
FAS
TGFB
TGFBR
LPS
CD14
DAXX
ASK2
ASK1
MKK3
MKK6
p38
TAB1
TAB2
TAK1
TRAF6
ECSIT
PP2CB
PP5
Sap1a
GADD153
MAX
MEF2C
PRAK
MAPKAPK
MSK1/2
Cdc25B
HSP27
CREB
DNA damage
GADD45
MEKK4
TAO1/2
NLK
Wnt signaling pathway
Proliferation, differentiation, inflammation
Apoptosis
Cell cycle
ERK5 pathway
Serum, EGF, reactive oxygen species, or Srk tyrosinkinase downstream
MEK5
ERK5
Nur77
+p
-p
MAPKKKK
MAPKKK
MAPKK
MAPK
Transcription factor
04010 1/18/16
(c) Kanehisa Laboratories

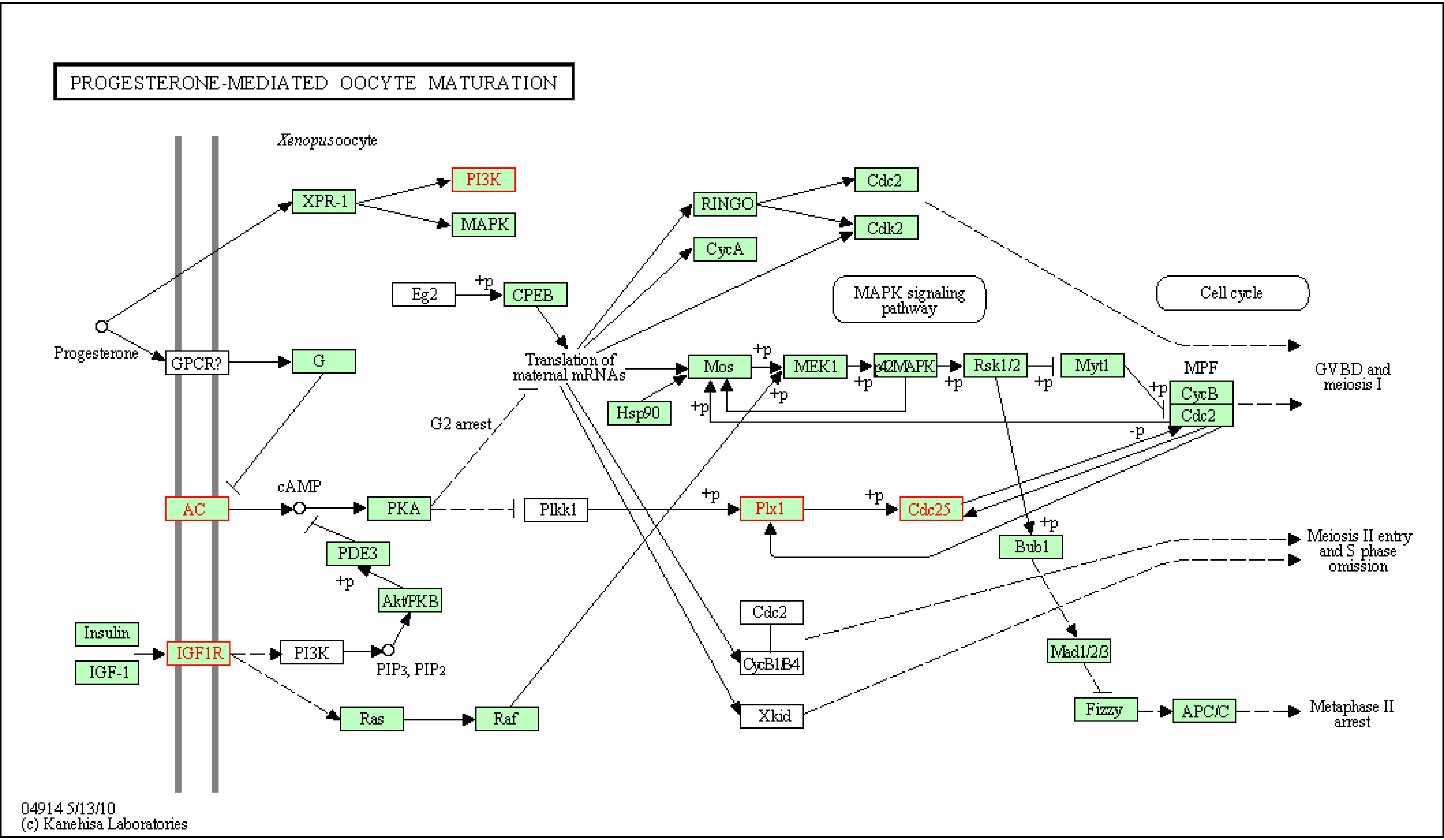

04914 5/13/10

(c) Kanehisa Laboratories

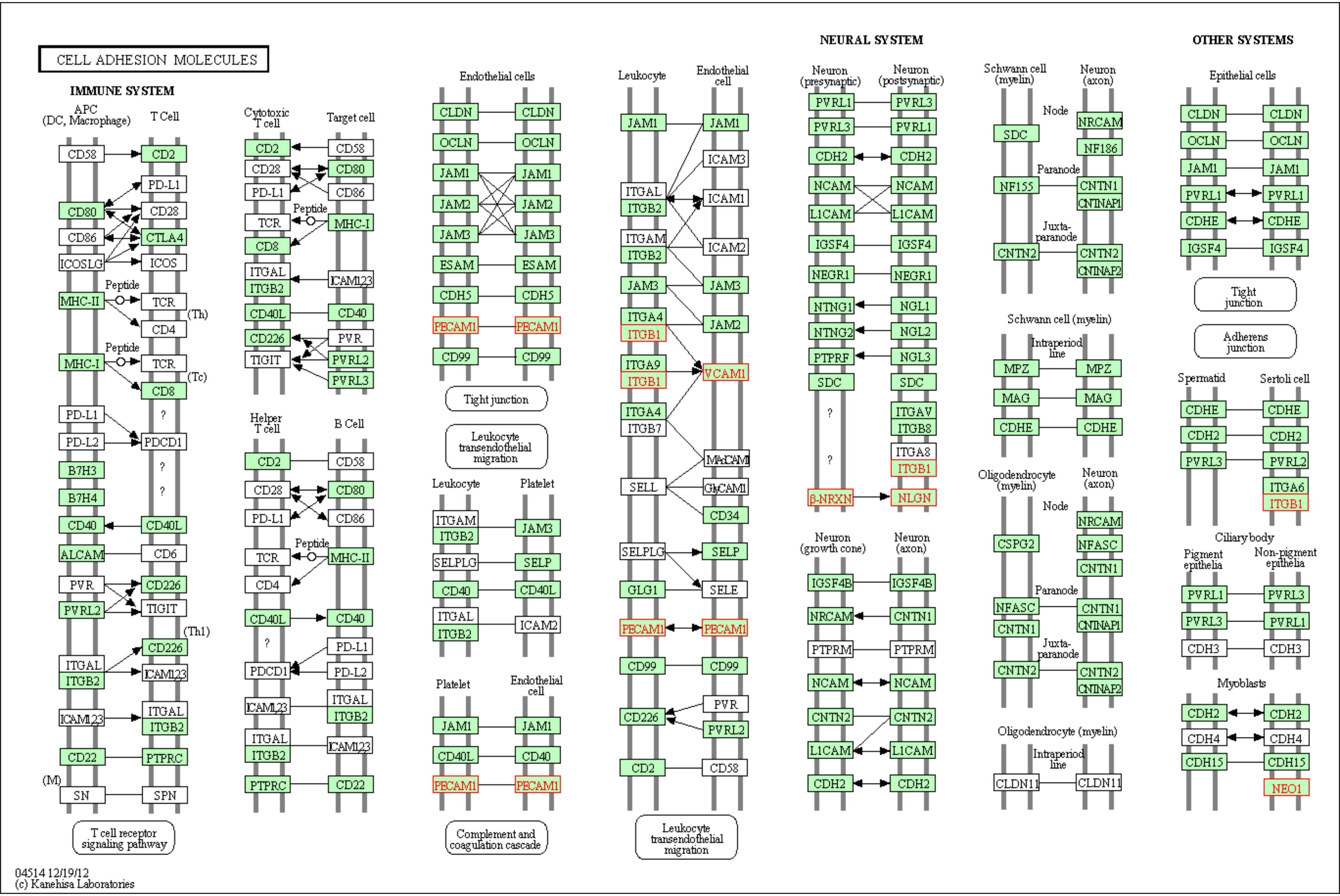
CELL ADHESION MOLECULES
IMMUNE SYSTEM
APC (DC, Macrophage)
T Cell
CD58
CD2
PD-L1
CD80
CD28
CD86
CTLA4
ICOSLG
ICOS
Peptide
MHC-II
TCR
(Th)
CD4
MHC-I
(Tc)
CD8
PD-L1
?
PD-L2
PDCD1
B7H3
B7H4
CD40
CD40L
ALCAM
CD6
PVR
CD226
PVRL2
TIGIT
(Th1)
ITGAL
ITGB2
ICAM123
ITGAL
ITGB2
CD22
PTPRC
(M)
SN
SPN
T cell receptor signaling pathway
Cytotoxic T cell
Target cell
CD2
CD58
CD28
CD80
PD-L1
CD86
TCR
Peptide
MHC-I
CD8
ITGAL
ITGB2
ICAM123
CD40L
CD40
CD226
PVR
TIGIT
PVRL2
PVRL3
Helper T cell
B Cell
CD2
CD58
CD28
CD80
PD-L1
CD86
TCR
Peptide
MHC-II
CD4
CD40L
CD40
?
PD-L1
PDCD1
PD-L2
ICAM123
ITGAL
ITGB2
ITGAL
ITGB2
ICAM123
PTPRC
CD22
Endothelial cells
CLDN
OCLN
JAM1
JAM2
JAM3
ESAM
CDH5
PECAM1
CD99
Tight junction
Leukocyte transendothelial migration
Leukocyte
Platelet
ITGAM
ITGB2
JAM3
SELPLG
SELP
CD40
CD40L
ITGAL
ITGB2
ICAM2
Platelet
Endothelial cell
JAM1
CD40L
CD40
PECAM1
Complement and coagulation cascade
Leukocyte
Endothelial cell
JAM1
ICAM3
ITGAL
ITGB2
ICAM1
ITGAM
ITGB2
ICAM2
JAM3
ITGA4
ITGB1
JAM2
ITGA9
ITGB1
VCAM1
ITGA4
ITGB7
MAdCAM1
SELL
GlyCAM1
CD34
SELPLG
SELP
GLG1
SELE
PECAM1
CD99
CD226
PVR
PVRL2
CD2
CD58
Leukocyte transendothelial migration
NEURAL SYSTEM
Neuron (presynaptic)
Neuron (postsynaptic)
PVRL1
PVRL3
PVRL3
PVRL1
CDH2
NCAM
L1CAM
IGSF4
NEGR1
NTNG1
NGL1
NTNG2
NGL2
PTPRF
NGL3
SDC
?
ITGAV
ITGB8
?
ITGA8
ITGB1
β-NRXN
NLGN
Neuron (growth cone)
Neuron (axon)
IGSF4B
NRCAM
CNTN1
PTPRM
NCAM
CNTN2
L1CAM
CDH2
Schwann cell (myelin)
Neuron (axon)
Node
SDC
NRCAM
NF186
Paranode
NF155
CNTN1
CNTNAP1
Juxta-paranode
CNTN2
CNTNAP2
Schwann cell (myelin)
Intraperiod line
MPZ
MAG
CDHE
Oligodendrocyte (myelin)
Neuron (axon)
Node
NRCAM
CSPG2
NFASC
CNTN1
Paranode
NFASC
CNTN1
CNTNAP1
Juxta-paranode
CNTN2
CNTNAP2
Oligodendrocyte (myelin)
Intraperiod line
CLDN11
OTHER SYSTEMS
Epithelial cells
CLDN
OCLN
JAM1
PVRL1
CDHE
IGSF4
Tight junction
Adherens junction
Spermatid
Sertoli cell
CDHE
CDH2
PVRL3
PVRL2
ITGA6
ITGB1
Ciliary body
Pigment epithelia
Non-pigment epithelia
PVRL1
PVRL3
PVRL3
PVRL1
CDH3
Myoblasts
CDH2
CDH4
CDH15
NEO1
04514 12/19/12
(c) Kanehisa Laboratories
