# Supplementary material for: MicroRNA miR-29 controls a compensatory response to limit neuronal iron accumulation during adult life and aging
Source: BMC Biol. 2017 Feb 13;15:9. doi: 10.1186/s12915-017-0354-x (PMC5304403; doi:10.1186/s12915-017-0354-x)
Supplement: Additional file 6: — Mir-29 predicted target DEGs identification in N. furzeri kif5a: sponge-29. A Intersection of D. rerio miR-29 predicted target genes (from TargetScanFish 6.2, score ≤ –0.30) with N. furzeri sponge-29 DEGs. A total of 38 predicted target genes were found deregulated in kif5a:sponge-29 fish, 28/38 upregulated (B) and 10/38 downregulated (C), respectively. Gray, red and green box represent a conserved binding site in the 3′-UTR of N. furzeri, M. musculus, and H. sapiens, respectively. Yellow colored genes lack a conserved binding site in N. furzeri. Light blue box indicate a physical interaction between target gene e and miR-29 observed by cross-linking immunoprecipitation (CLIP-seq). 1-3-4 numbers are relative to gene dataset type in Fig. 3e. (PDF 78 kb) [file 12915_2017_354_MOESM6_ESM.pdf]

A

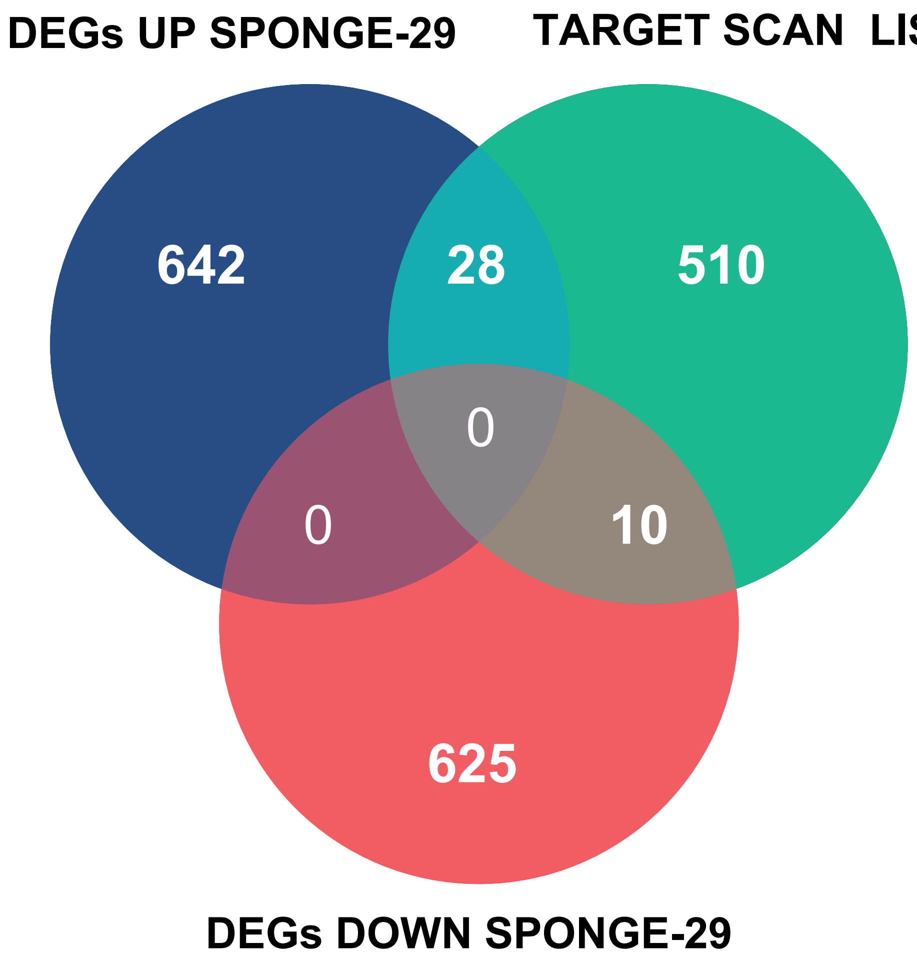

B

Predicted mir-29 targets **up-regulated**

KDM6BB					4
DOT1L					4
EPC1					
FRAS1					
GRIP1					4
LAMC1					4
PNPLA3					1
PER2					
HBP1					1
AHSG					
ANKRD50					
BGNB					
CU571169.4					
ACY1					4
WU:FB52C12					
HMCN2					
CDKL1					1
CU638738.1					
PHC2B					
HYAL4					1
NEFL					4
IGFBP2A					
TMEM38A					
TLX3B					
MARCH9					
FHOD3 (1 OF 2)					
AEBP1 (2 OF 2)					

C

Predicted mir-29 targets **down-regulated**

TET3					3
DNMT3AB					3
DNMT3B					3
MEX3B					3
COL4A1					3
NSD1A					3
BACH2B					3
XKR7(2 OF 2)					
RCC2					3
DIEXF					3

Binding site conserved in *N. furzeri*

Binding site conserved in *M. musculus*

Binding site conserved in *H. sapiens*

Interaction observed with CLIP-seq assay

Expression not influenced by aging

1-3-4 Referred to gene dataset type of fig. 3E
